# The genus *Amegilla* (Hymenoptera, Apidae, Anthophorini) in Australia: A revision of the subgenera *Notomegilla* and *Zonamegilla*

**DOI:** 10.3897/zookeys.653.11177

**Published:** 2017-02-08

**Authors:** Remko Leijs, Michael Batley, Katja Hogendoorn

**Affiliations:** 1 South Australian Museum, North Terrace, Adelaide, SA 5000, Australia; 2 Australian Museum, 6 College Street, Sydney NSW 2010, Australia; 3 School of Agriculture, Food and Wine, The University of Adelaide, SA 5005, Australia

**Keywords:** blue-banded bee, pollinator, taxonomic revision, mtDNA phylogeny

## Abstract

The Australian bees in the subgenera *Notomegilla* and *Zonamegilla* of the genus *Amegilla* are revised. Commonly in Australia the species in these subgenera are called blue-banded bees, although not all species have blue bands. A phylogeny based on mitochondrial cytochrome oxidase 1 sequence data was used to delineate the species and a set of morphological criteria was developed for species identification. Strong support was obtained for separating the Australian species into the three subgenera previously proposed on the basis of morphology. Two species, are recognised in the subgenus Notomegilla and eleven new synonymies are proposed. Twelve Australian species are recognised in the subgenus Zonamegilla including four new species: *indistincta*, *karlba*, *paeninsulae* and *viridicingulata*, and twenty new synonymies are proposed. Keys to the species of both sexes and descriptions or redescriptions of all species are provided. Distribution maps, data on flower visitation and phenology are given.

## Introduction

Many species in the genus *Amegilla* are conspicuous because of their colourful, iridescent hair bands, relatively large size and hovering flight patterns. Some species are known to nest in sand, loam or clay soils, and in soft sandstone, clay washouts and mud bricks. Nests are often found in large aggregations that are re-used for many generations (Michener 1960, Cardale 1968). Their bright colours, nearly ubiquitous presence in Australia, readiness to forage from exotic flowers in suburban gardens and nesting behaviour ensure that this genus is well-recognised by the general public. Several species belonging to the subgenera *Zonamegilla* and *Notomegilla* are referred to as ‘blue-banded bees’ (Rayment 1935, Dollin et al. 2000).

The life-cycles of the different species seem to have many similarities. With a life-span of approximately 6 weeks, adult *Amegilla* are relatively short-lived. Depending on the species and the suitability of the climate, one or several generations are produced during a flight season, while immatures survive the unfavourable season in the prepupal stage in their cells (Michener 1965, Cardale 1968). *Amegilla* species visit a large range of flowering plants, and belong to the group of buzz pollinating bees (Buchman 1983), which makes them suitable Australian native pollinators for solanaceous crops, such as tomato, eggplant and pepper (Bell et al. 2006, Hogendoorn et al. 2006, 2010). Members of the genus are among the main pollinators of Australian *Solanum* species in the arid zone (Anderson and Symon 1988) which include a number of small-range endemic species and culturally significant species such as *Solanum centrale* or bush tomato (Symon 1979).

The taxonomy of the genus *Amegilla* has had a long and colourful history. *Amegilla zonata* (Linnaeus, 1758) was among the first organisms to receive a Latin binomial. The first Australian bee species to be described, *Amegilla cingulata* (Fabricius, 1775) was collected by Sir Joseph Banks. Rayment (1944, 1947, 1951) recognised three groups of species, which were later formalised by Brooks (1988) as the subgenera *Asaropoda*, *Notomegilla*, which are endemic to Australia and Papua New Guinea, and *Zonamegilla*, which has a wide distribution throughout Eurasia, S.E. Asia, and Australia (Michener 2007). Because of the difficulty of separating females of different species, Michener (2007) chose not to recognise subgenera, but acknowledged that the names might be useful to define species-groups within this very large genus.

Prior to 1940, species confusion and name changes were frequent, due in no small part to morphological similarities between species. In addition, many morphological character traits of *Amegilla* seemed variable, both within and between species, which made it difficult to find reliable characters to distinguish the species. Conscious of this intraspecific variation, Rayment (1935) disagreed in print with his mentor, Cockerell and warned of the dangers of using hair colour as a character. Rayment later published two papers (Rayment 1944, 1947) in which he used new characters in an attempt to identify the Australian species. The diagnostics used by Rayment relied heavily on sculpture on the pygidial plate of females and on small differences in the extent of pale face marks of males. Unfortunately, these characters were unreliable: the sculpture of the pygidial plate is subject to wear with age, and the face marks of males vary within species. Rayment described 24 new species and some subspecies, several from the same localities (Cardale 1993). Although his descriptions are often well illustrated, the lack of identification keys and adequate diagnostic traits of the species make it virtually impossible to properly identify these species. Thus, Rayment’s (1944, 1947) descriptions contributed to a new level of confusion in a group that consists of morphologically similar species. Revising this group was further complicated by problems with the identity of type material. Instead of assigning single specimens as type specimen, Rayment often referred to series of specimens when defining new names. In addition, information on specimen labels often differed from that given in the published descriptions, and most males in Rayment’s ‘type series’ had their apical sternites and genitalia dissected. As many of these subsequently became lost, the morphological information of type series is incomplete.

Here we present a revision of the species in the subgenera *Notomegilla* and *Zonamegilla* based on examination of the majority of the type material and supported by molecular phylogenetics based on mitochondrial DNA of all currently recognised Australian species sampled throughout their geographical ranges.

## Material and methods

### Specimens examined

This study is based on examination of museum specimens. The following acronyms are used in the supplementary information associated with this paper:

ABTC
 Australian Biological Tissue Collection, South Australian Museum, Adelaide 


AMS
 Australian Museum, Sydney 


ANIC
 Australian National Insect Collection, Canberra 


AQIS
 Australian Quarantine and Inspection Service, Cairns 


ASCU
 Orange Agricultural Institute, Agricultural Scientific Collections Unit 


BMNH
 The Natural History Museum, London 


MAGNT
 Museum and Art Gallery Northern Territory, Darwin 


MVMA
 Museum of Victoria Entomology, Melbourne 


SAMA
 South Australian Museum, Adelaide 


TMAG
 Tasmanian Museum and Art Gallery, Hobart 


QM
 Queensland Museum (now including the former UQIC, University of Queensland Insect Collection), Brisbane 


WAM
 Western Australia Museum, Perth 


WINC
 Waite Insect and Nematode Collection, Adelaide University, Waite Campus, Adelaide 


Information from the labels of museum specimens was copied in a single database, which was subsequently used to generate distribution maps, analyse species phenology and flower records. The database is available as supporting information associated with this publication (Suppl. material 2).

Fresh specimens were collected throughout Australia for molecular analysis by the authors and by numerous other people acknowledged below. Special attention was given to type localities for named species and localities known from apparently undescribed species in museum collections. Some non-Australian Amegilla (Zonamegilla) species were included to enable inferences about the historical biogeography of this subgenus. Collected specimens were killed and preserved in absolute ethanol to allow DNA extraction at a later stage. Ethanol-preserved specimens, as well as extracted DNA, are kept in the Australian Biological Tissue Collection at the South Australian Museum. DNA voucher specimens are kept in the Entomology Collection of the South Australian Museum. Locality data, voucher numbers and GenBank Accession numbers of specimens used in the molecular analyses are available as supporting data associated with this publication (Suppl. material 1: Table S1).

### Taxonomic methods

**Figure 1.**
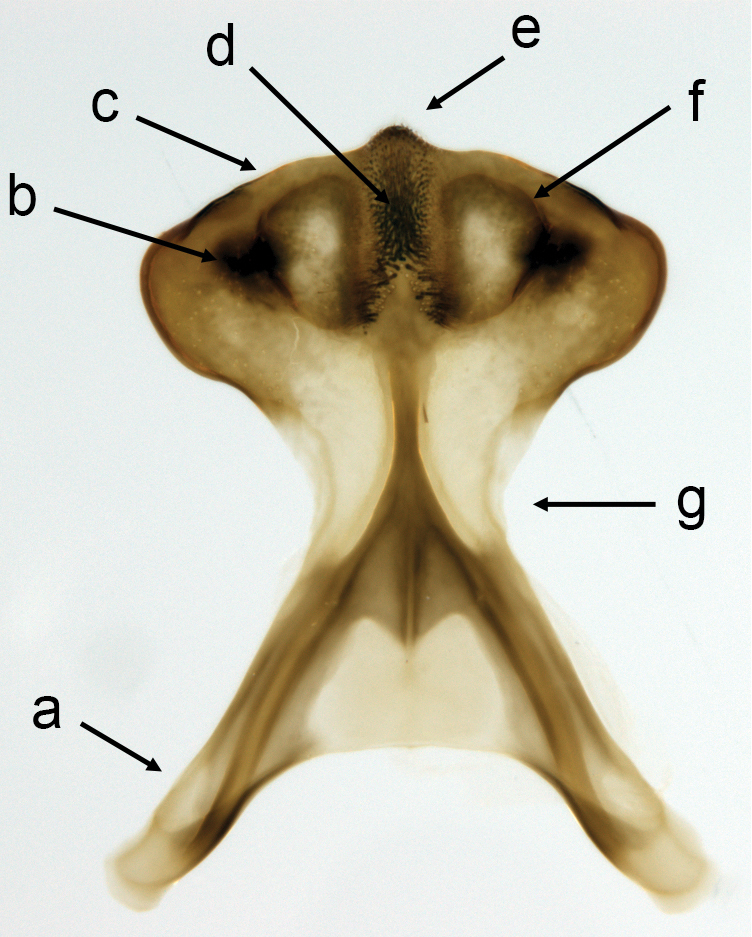
Characters of S7 of male *Zonamegilla* species, ventral view. The broadly oval *head* at the posterior end of S7 has a medial *excavation* in the dorsal surface laterally and a longitudinal elevation or *ridge* on the ventral surface. The excavation is carinate laterally and the sclerotisation between the carinae and the ventral ridge is usually thinner than the surrounding areas creating *windows* in the pigmentation. Most of the medial ridge is covered with short hairs which become longer and denser towards the middle of the head, creating a T- or Y-shaped *brush*. **a** apodeme **b** ventral ridge **c** posterior margin **d** medial ridge **e** apical projection **f** lateral carina of dorsal excavation **g** neck.

Genitalia were extracted from a number of male museum specimens, after relaxation for up to three days in a humid container with chromocresol or thymol added as a fungal inhibitor. Dissected genitalia were treated with 10% cold sodium hydroxide for 24 hours, glacial acetic acid for approximately one hour and stored in glycerol to facilitate the study of morphology. Digital photography using an automontage system was used to obtain images of relevant characters. Morphological terminology follows that of Brooks (1988) and Michener (1944, 2007). Terminology used for integumental sculpture is that of Houston (1975) or Harris (1979). Metasomal terga are referred to as T1, T2 etc., sterna as S1, S2 etc., individual flagellar segments as f1, f2 etc. The following descriptive abbreviations are used: IOD, interocellar distance; OOD, ocellocular distance; OS, shortest distance between medial and lateral ocelli. The seventh, hidden, sternum of males exhibits useful diagnostic characteristics and was extracted for examination. Terms used by Brooks (1988) to describe S7 were of limited use for *Zonamegilla* species and were supplemented by those illustrated in Fig. 1.

The description of, and differentiation between, intra- and interspecific colour variation has for a long time bedevilled the taxonomy of this group of bees. Colour variation in both the iridescent hairs of the tergal bands and the non-reflective hairs of the mesosoma are found in most species and can, in some cases, obscure genuine intraspecific colour differences. The metallic interference colours, which are produced by reflection from arrays of microtubules (Fung 2005), are easily dulled by damage to the scale-like setae that produce them. Species descriptions are, therefore, reported for selected fresh specimens, followed by discussion of intraspecific variation.

### Incorporating phylogenetics in a taxonomic revision

To overcome the difficulty of separating interspecific from intraspecific morphological variation, we used partial mitochondrial CO1 gene sequences, as in the ‘barcoding’ approach (Hebert et al. 2004), to delineate species boundaries. Voucher specimens from the molecular clades were then compared with type material in order to determine the corresponding species names. Morphological character sets for each species were then re-defined and geographical distributions were determined from the sequenced specimens as well as from specimens in the major Australian insect collections. All Australian species have been re-described, where possible based on DNA voucher specimens, lodged in the South Australian Museum, Adelaide.

### DNA methods

DNA extraction, PCR amplification and sequencing were performed as described in Cooper et al. (2002). Two regions of the mitochondrial genome were amplified. An 822 bp region of the 3’ end of the cytochrome oxidase subunit 1 (CO1) gene was amplified using primers M202 (forward, 5’-CAA CAT TTA TTT TGA TTT TTT GG-3’) and M70 (reverse, 5’-TCC ATT GCA CTA ATC TGC CAT ATT A-3’) (alias Jerry and Pat, Simon et al. 1994), as well as a 648 bp fragment just upstream of the previous fragment of CO1 using primers M414 (5’-GGT CAA CAA ATC ATA AAG ATA TTG G-3’) and M423 (5’-TAA ACT TCA GGG TGA CCA AAA AAT CA-3’) (LCO1490 and HCO2198: Folmer et al. 1994). ChromasPro version 1.34 (Technelysium Pty Ltd, Tewantin, QLD, Australia) was used to edit chromatogram files, to determine consensus sequences from both strands, and to align sequences across specimens. DNA sequences are available from GenBank: KY485622–KY485915.

### Phylogenetic analyses

Phylogenetic analyses of aligned sequence data were carried out using the program PAUP* version 4.0b8 (Swofford 2001), MRBAYES v.3.1 (Huelsenbeck and Ronquist 2001) and BEAST version 1.4.8 (Drummond and Rambaut 2007). PAUP* was used for generating and editing data matrices as well as error proofing using neighbour-joining runs, and for analyses of uncorrected sequence divergence. For each species, as recognized by neighbour-joining analyses and morphology, a small number of representatives across their geographic range were selected for Bayesian phylogenic analyses. Bayesian analyses were performed using MRBAYES and BEAST. The analyses were performed on combined CO1 sequence fragments, applying unlinked data partitions for each of the codons for the CO1 gene and using a general time reversible model of sequence evolution with invariable sites and gamma distributed rates across sites (GTR + i + g). Tracer v1.4 (Rambaut and Drummond 2007) was used to check that the effective sample sizes (ESS) of the parameters during the Baysian runs were larger than 100.

A BEAST analysis allowed the application of relaxed molecular clock methods in order to obtain estimates of node divergence times. Because fossils are not known for *Amegilla* bees a mean rate of 0.0115 substitutions per site per million years (Brower 1994) was used in analyses with an uncorrelated log-normal molecular clock and a Yule process of speciation. The analyses were performed with 40 million generations, sampling trees every 1000 generations. Parameter estimation and calculations of the >50% posterior probability consensus tree was done after discarding the first 4000 saved trees.

MRBAYES analysis was performed with four simultaneous chains, sampling tree topologies and parameters every 50 generations. After 2,029,200 generations all parameters had reached their ESS, and the potential scale reduction parameter was approximately one for all parameters, indicating that the Bayesian runs had converged and that a sufficient sample of the posterior distribution had been obtained. A burn in of 10,000 sampled trees was chosen for each independent run of MRBAYES. A >50% posterior probability consensus tree was constructed from the remaining 61,170 saved trees (30,585 trees per individual run).

## Results and discussion

### Species delineation and molecular phylogeny

**Figure 2.**
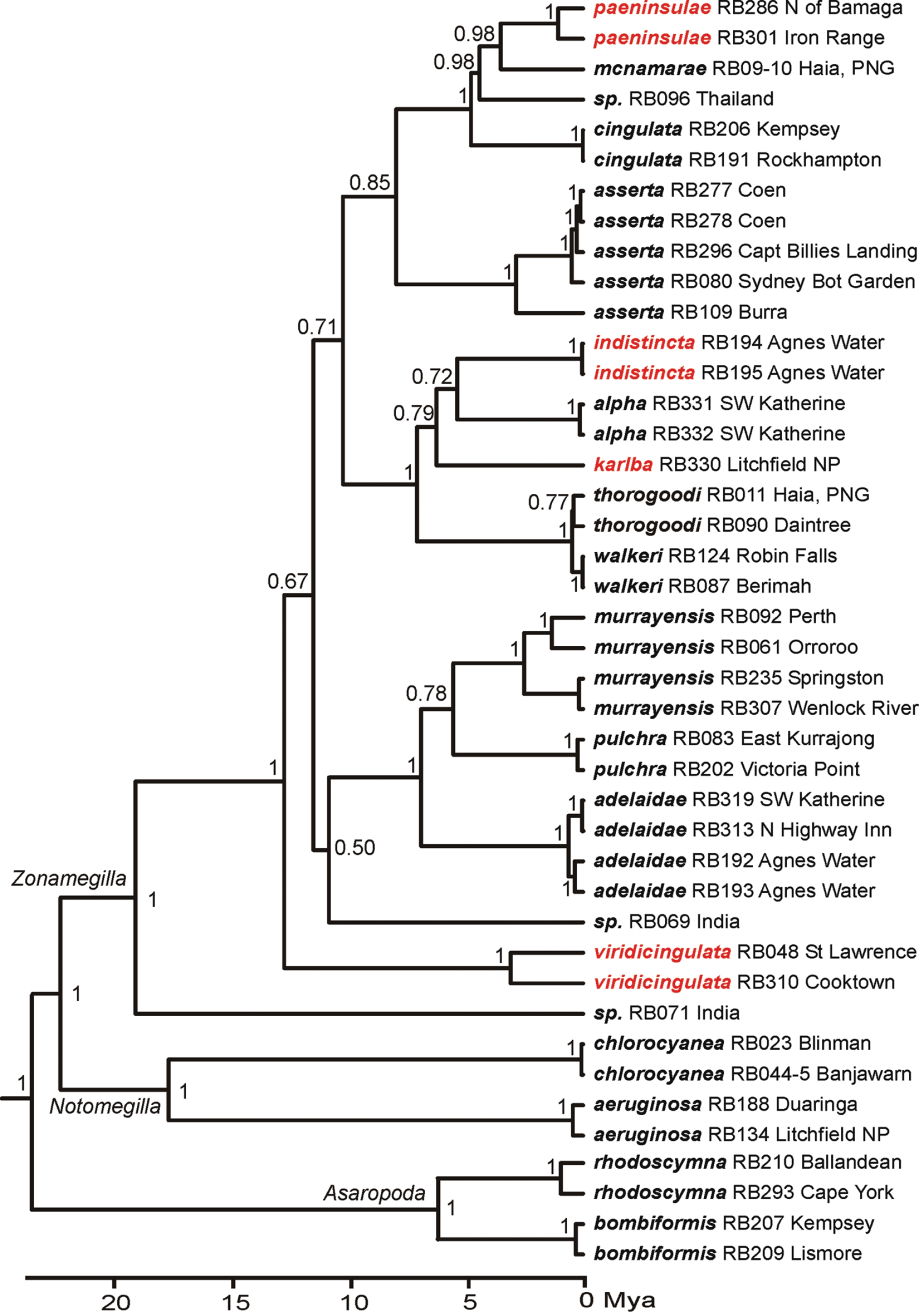
Baysian molecular phylogenetic consensus tree of combined CO1 sequences. Node support values (posterior probabilities) are shown near the nodes. The terminals are labelled with species names and RB-numbers, which refer to Table S1, and a locality indication.

Phylogenetic analyses of CO1 gene sequences, using neighbour-joining and parsimony in PAUP* and Bayesian methods in MRBAYES and BEAST resulted in very similar tree topologies with three monophyletic groups (Fig. 2, Suppl. material 1: Figs S1, S2) corresponding to the three subgenera proposed by Brooks (1988). The analyses in general resulted in well-resolved clades that provided the basis for identification of morphological characteristics for each of the proposed species. However the relationships between the subgenera and species-groups within the subgenus Zonamegilla were incompletely resolved (posterior probabilities 0.50–0.85), which is not surprising, considering that these analyses were based on mitochondrial data only.

Morphological and molecular evidence indicates the existence of only two valid species in *Notomegilla*, and twelve valid Australian species in *Zonamegilla*. With two exceptions the uncorrected pairwise distances between specimens resulted in unambiguous delineation of the species with maximum intraspecific distances mostly less than 4.5% and interspecific distances greater than 5.8% (Table 1). The first exception concerns the species *Amegilla walkeri* (Cockerell) and *Amegilla thorogoodi* (Rayment), which have very shallow uncorrected pairwise sequence divergences of 0.62–1.37% (Table 1). Although closely related, these species are readily recognised by colour differences and are geographically separated (Fig. 13) by the proposed Carpentarian barrier (Joseph and Omland 2009 and references therein). Geographical separation of the colour forms is complete with no evidence of intermediates. The estimated divergence time between the species (Fig. 2) corresponds well with the Pleistocene age of the barrier inferred in previous studies of birds and rodents (Jennings and Edwards 2005, Lee and Edwards 2008, Breed and Ford 2007). Several similar examples of such recent divergences are known for birds (Joseph and Omland 2009), blowflies (Wallmann et al. 2005) and grasshoppers (Carsten and Knowles 2007).

**Figure 3–14.**
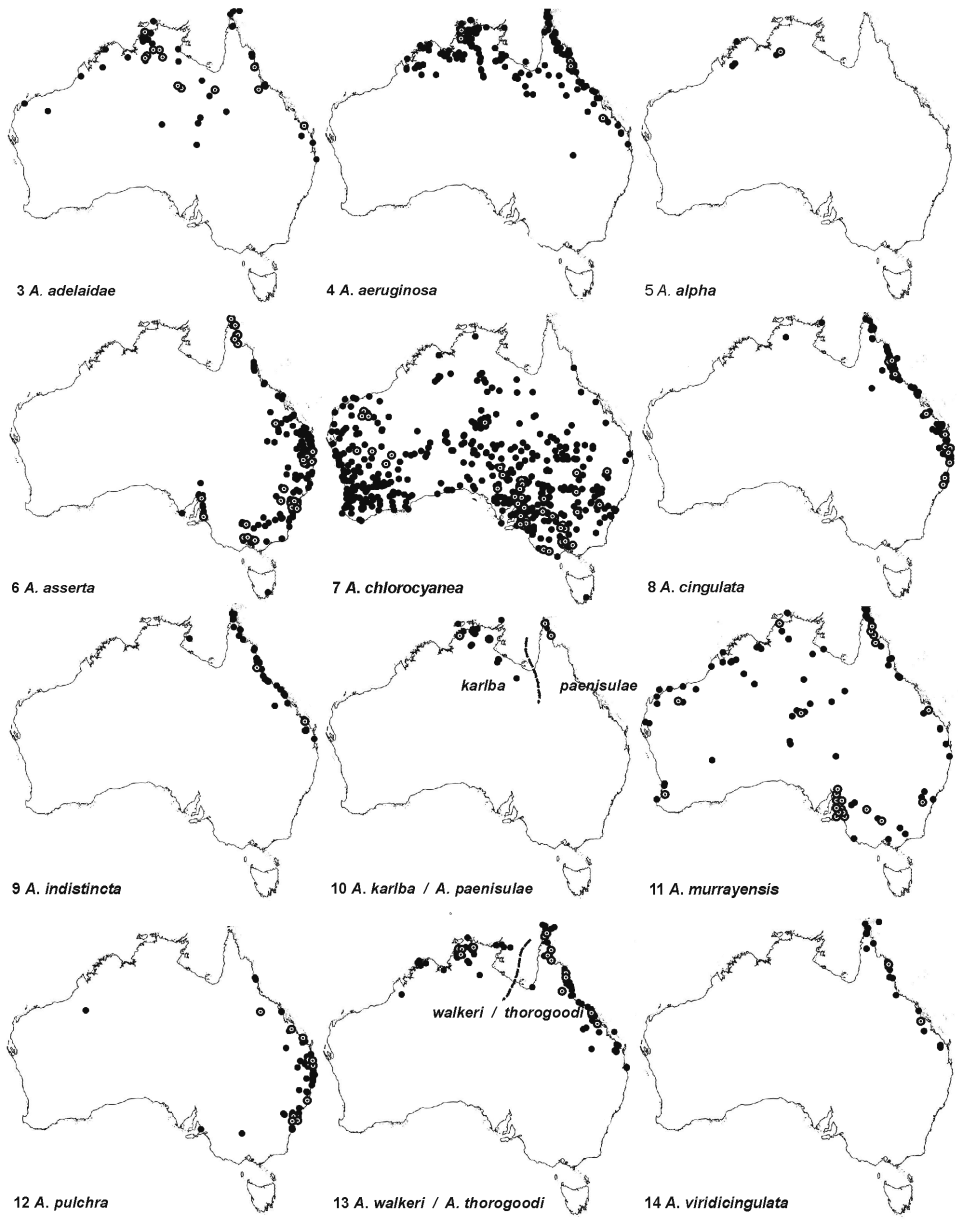
Distribution maps ● indicate museum record, ʘ indicates DNA specimen.

The second exception involved difficulties with delineation within the species-group that includes *Amegilla pulchra* (Smith), *Amegilla murrayensis* (Rayment) and *Amegilla adelaidae* (Cockerell). In all analyses, the group as a whole was well defined with *adelaidae* as sister to the other two species. Within the group, initially analyses of sequences obtained using the M202/M70 primers resulted in two clades for *murrayensis* and a single clade with rather large intraspecific divergences for specimens of *pulchra* (Suppl. material 1: Fig. S1, Table 1). Analysis of sequences obtained using the Folmer primers showed a single clade for *murrayensis*, but did not resolve the problems for *pulchra* (Suppl. material 1: Fig. S2). There are a number of explanations possible for these observations: First, it is likely that there is a nuclear paralogue in the sequenced mtDNA fragments (Bensasson et al. 2001, Pamilo et al. 2007, Song et al. 2008) that evolved in the common ancestor of *pulchra* and *murrayensis*. Evidence for this are double base pair readings found predominantly at third codon positions, but these never resulting in stop codons. Secondly, the observed phylogenetic patterns may be resulting from *Wolbachia* infestations that were noted to have distorted phylogenetic patterns in other Hymenoptera (Klopfstein et al. 2016). It was beyond the scope of this study to further investigate the source of these problems.

**Table 1.** Maximum percentage uncorrected pairwise intraspecific sequence divergence of Australian *Amegilla* and distances to nearest other species clade A) based on 822 bp fragment of CO1 using primers M202/M70, B) based on 648 bp amplified using primers M414/M423. ‘-‘: no data available. ‘*’: inflated pairwise divergence due two multiple clades possible resulting from nuclear paralogues. (See also supplementary Figs S1 and S2)

Subgenus		% maximum intraspecific divergence A	% distance to nearest other species clade A	n	% maximum intraspecific divergence B	% distance to nearest other species clade B	n
*Notomegilla*	*aeruginosa*	0.4	17.2	7	-	-	-
	*chlorocyanea*	2.6	15.5	39	-	-	-
*Zonamegilla*	*adelaidae*	2.5	7.2	8	0.3	6.6	2
	*asserta*	3.7	7.2	35	2.2	5.8	5
	*alpha*	-	-	-	0.2	6.8	2
	*thorogoodi*	0.5	0.6	4	1.3	-	12
	*cingulata*	0.5	6.8	13	0.0	6.5	2
	*indistincta*	0.5	7.2	3	0.3	6.8	2
	*murrayensis*	9.4*	9.8*	56	1.3	5.3	19
	*karlba*	-	-	-	-	7.5	1
	*paeninsulae*	-	-	-	3.9	6.7	4
	*pulchra*	9.3*	9.8*	15	8.6*	5.3	13
	*walkeri*	0.8	0.6	7	-	-	-
	*viridicingulata*	4.5	9.0	4	-	7.3	1

Bayesian phylogenetic analysis (Fig. 2) indicates that the Australian *Zonamegilla* species are not monophyletic with respect to Asian species. Although the relationships among these species and some isolated taxa from Australia and Asia were incompletely resolved (posterior probabilities 0.50–0.85), it is clear that migration of *Zonamegilla* species between Australia and the region to the north has involved more than one event.

One well-supported species-group within *Zonamegilla*, which includes *cingulata* (Fabricius), *paeninsulae* sp. n., *mcnamarae* (Cockerell) from Papua New Guinea and a species from Thailand, comprises species with similar morphological characteristics including brightly coloured hair bands on the posterior margins of the terga and almost uniformly coloured hairs on the scopa of the hind tibia of the females. Among the Australian species, only *viridicingulata* sp. n. shares this combination of characters, but it is present in a number of Asian species. The species *asserta* (Cockerell) is sister to the *cingulata* group.

Another well-supported clade that includes *Amegilla alpha* (Cockerell), *thorogoodi*, *indistincta* sp. n., *karlba* sp. n. and *walkeri* consists of species that are superficially rather different in appearance. Most notable is the orange-brown hair covering most of the metasoma in *alpha*, which led Cockerell to suggest that it was a subspecies of Amegilla (Asaropoda) bombiformis (Smith). A third species-group, *adelaidae*, *murrayensis* and *pulchra*, contains species with partially overlapping distributions (Figs 3, 11, 12).

### Morphological characters

The most useful diagnostic characters that emerged from this revision were the length of the basitibial streak on the hind leg of females, the size, shape and colour of the medial patch of pubescence on the female T5, and the shape of the male S7. The colour of the scutal pubescence, the tergal hair bands and the pubescence on female T5, although sufficiently reliable characters in fresh specimens, are less reliable in older or worn specimens. Similarly, the shape of the yellow face markings, especially in males, although often a useful indicator, can be aberrant in a small number of individuals. Characters of the male genital capsule have not been used in this study, because obvious differences between species were not evident.

### Phenology

Climate seems to determine the phenology of the species. Those species that have a wide latitudinal distribution (*Amegilla chlorocyanea* (Cockerell), *Amegilla asserta* (Cockerell) and *murrayensis*) are active in summer in the south of the continent, but are found year-round in the north. Species that occur only in the north of the country are most active in May and June. Along the eastern seaboards of south Queensland and New South Wales, activity peaks in October and May.

### Floral records

**Figure 15.**
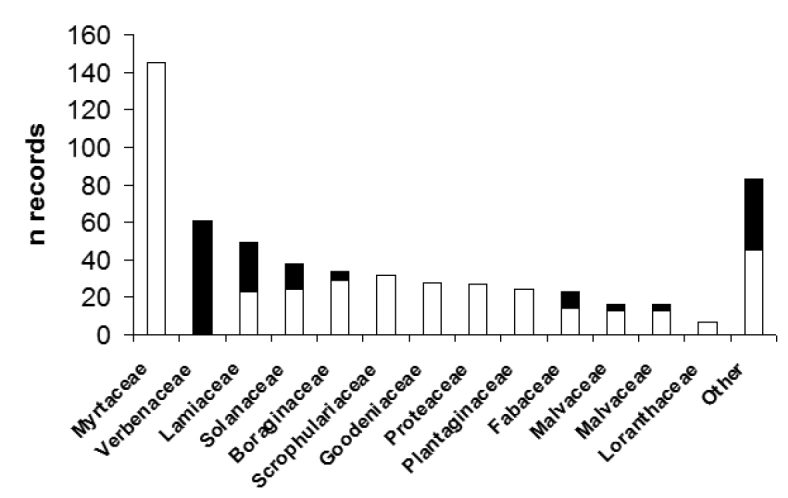
Floral records of blue-banded bees caught on native (white) and introduced (black) plants, arranged by family. The category “Other” contains records from 36 plant families.

Floral records were available for a total of 583 specimens, by far the most for *Amegilla chlorocyanea* (n = 447), followed by *Amegilla aeruginosa* (Smith) (n = 37), *murrayensis* (n = 36) and *thorogoodi* (n = 21). The number of records available for other species ranges from 1 to 17. The bees visited 147 plant species of 47 different families (Fig. 15). Thus, blue-banded bees are highly polylectic. Because 24% of all plants visited were introduced, there seems to be no clear preference for either native or introduced plants. Blue-banded bees are often observed on the following introduced garden plants and weeds: English lavender (*Lavandula angustifolia*), tomato (*Solanum lycopersicum*), Duranta (*Duranta erecta*), various blue-flowering members of the *Boraginaceae and Lamiaceae*, snakeweed (*Stachytarpheta jamaicensis*), and lantana (*Lantana camara*). In the arid zone, *Amegilla*, especially *chlorocyanea*, is frequently found on *Eremophila* sp., *Ptilotus* sp., *Solanum* sp., *Stemodia florulenta* and *Trichodesma zeylanicum*.

### Evolution and historical biogeography of *Amegilla* in Australia

The phylogeny estimated using BEAST (Fig. 2) makes it possible to discuss hypotheses about the evolution and historical biogeography of *Amegilla* in the Australian region. The subgenus Notomegilla is endemic to Australia and Papua New Guinea. Its species *chlorocyanea* and *aeruginosa* diverged circa 17 Mya and have almost non overlapping geographical distributions: *chlorocyanea* is widespread in southern and in arid Australia (Fig. 7) while *aeruginosa* is adapted to subtropical and tropical environments of northern Australia and Papua New Guinea. It is possible that the divergence between the two species was triggered by aridification of inland Australia, which started around 15 Mya (Martin 2006). Adaptation to the unpredictability and patchiness of precipitation in the arid zone would include the propensity to migrate over large distances in search for suitable habitat. The fact that long distance dispersals may indeed be common in *chlorocyanea* is indicated by the lack of geographical signal and the sharing of mitochondrial haplotypes between sample localities thousands of kilometres apart (see NJ-phylogeny in the supplementary information).

The Australian species within the subgenus Zonamegilla are probably not monophyletic. This is indicated by species from India, Thailand and Papua New Guinea occurring amongst the Australian species (Fig. 2). Although, there are unresolved nodes at the base of the Australian clades, it is possible to infer that *Zonamegilla* colonised Australia around 12 Mya. The Australian *Zonamegilla* fauna has close links with those of Papua New Guinea as is also known for large carpenter bees, *Xylocopa*, of which species from Papua New Guinea and Australian form a monophyletic group (Leys et al. 2002). Two of the Australian species of *Amegilla* are also found in Papua New Guinea: *Amegilla aeruginosa* which is a species found in the wet tropics but also in dry grassland areas (Fig. 4); the other species, *Amegilla thorogoodi* is mainly distributed in the wet tropics along the east coast of Queensland and in southern Papua New Guinea. The close relationship between *Amegilla thorogoodi* and *Amegilla walkeri* (see above) is very likely the result of isolation and speciation in the Pleistocene, when populations of an ancestor adapted to the wet tropics became isolated east and west of the Gulf of Carpentaria, because dry grassland south of the Gulf of Carpentaria became a barrier. The close relationship between Australian and Papua New Guinea *Amegilla* species is also evidenced by the species from the *cingulata* group, including *mcnamarae* from Papua New Guinea. Most of the Australian *Zonamegilla* species have coastal distributions. The exceptions are *asserta* which has an east coast and Bassian distribution (Fig. 6), and *murrayensis* which is the only species that is widespread across the entire arid zone (Fig. 11) and, like *chlorocyanea*, shows a lack of geographical signal in mitochondrial haplotypes. This suggests that *murrayensis* also is able to migrate over large distances.

## Systematics

### Decisions for Synonymy - Problems with type specimens for Rayment’s names

In all but three cases, Rayment described his new species without reference to a type specimen, but listed a number of dates, localities and collectors from which a series of presumed syntypes could potentially be identified. He also left a considerable number of specimens with handwritten labels containing a species name and the word “type” or “allotype”. Only a fraction of the material referred to in Rayment’s descriptions was located and in many cases the dates given in the published information differed from those on the specimen labels (though this is not necessary for a specimen to be considered as a syntype). Although these circumstances made it tempting to treat all Rayment’s names as *nomina dubia*, presumed syntypes were identified, primarily from specimens with identification labels in Rayment’s distinctive hand and collection dates before February 1941, the latest date recorded in Rayment’s manuscripts. For type series containing more than one species a lectotype was chosen, though the published descriptions were of little assistance in deciding which species should be selected. The consequences of these decisions were minimal as all but two of Rayment’s names were synonymised. For each species the decision for synonymy is discussed in detail in the systematic section below.

The specimens from examined museum collections as well as newly collected material have been data-based, and were used to generate distribution maps (Figs 3–14). The data are available as online supporting information. The major outcome of this study is that of the 43 available specific names, 33 were synonymised, leaving two species in the subgenus Notomegilla and thirteen Australian species in the subgenus Zonamegilla, including four new Australian species.

### Excluded species

The whereabouts of type material for the following species is unknown or is in such condition that they could not be distinguished from other species by morphology or the original description.

***Anthophora adelaidae ernesti* Rayment**

*Anthophora adelaidae ernesti* Rayment, 1947, p. 47.

*Amegilla ernesti* (Rayment), Michener, 1965, p. 216.

No type material bearing this name was located. Although the name was synonymised with *adelaidae* (Brooks, 1988) no justification was given.

***Anthophora berylae* Rayment**

*Anthophora berylae* Rayment, 1947, p. 49.

*Amegilla berylae* (Rayment), Michener, 1965, p. 216.

Amegilla (Zonamegilla) berylae (Rayment), Brooks, 1988, p. 511.

Type material for *berylae* was not found.

***Anthophora hackeri* Rayment**

*Anthophora hackeri* Rayment, 1947, p.55.

*Amegilla hackeri* (Rayment), Michener, 1965, p. 216.

Amegilla (Zonamegilla) hackeri (Rayment), Brooks, 1988, p. 511.

Syntypes of *hackeri*: male, Mossman, Queensland, 5 May 1940, “Type”, ANIC 32-034557; female, same information as male, “Allotype”, 32-034556.

While the specimens were undoubtedly the syntypes from which the species was described, they could not be distinguished from other species as the hidden sterna and genital capsule of the male are missing and the hair patch on T5 of the female is badly worn. The specimens may eventually prove to be conspecific with *thorogoodi* or *indisctincta* if ancient DNA sequence methods can be applied.

**Amegilla (Zonamegilla) zonata (Linnaeus)**

*Apis zonata* Linnaeus, 1758, p. 576.

This name has frequently been applied to Australian species and has appeared in checklists long after Cockerell’s (1931) suggestion that its use might be inappropriate. Now that the identity of *Amegilla zonata* has been firmly established (Baker 1996), we can confirm that no specimens from Australia have been located to which this name might be applied.

***Anthophora zonata cincta* Sichel**

*Anthophora zonata cincta* Sichel, 1869, p. 58.

No type material associated with this name was located. Smith (1879) pointed out that Sichel had incorrectly applied the name *cincta* to an Australian species and proposed *emmendata* as a replacement name. Rayment (1947) later proposed *fabriciana* as another replacement name, believing that Smith had incorrectly interpreted Sichel’s length measurement as a printer’s error. It is unlikely that either author examined Sichel’s specimen, but both described other material associated with their new names and that material has been located. We initially have, therefore, treated *emmendata* and *fabriciana* as species, but have synonymised both with *cingulata* (see below). We believe Smith (1879) correctly interpreted Sichel’s size measurement as an error, as a similarly improbable size is given in that author’s description of *Amegilla chlorocyanea* (as *Anthophora cingulata*) (Sichel 1869).

### Key to the Australian subgenera of *Amegilla* and the Australian species in the subgenera of *Notomegilla* and *Zonamegilla*

Note: The following key is based on the species re-described in this paper and the majority of the specimens examined were in good condition. Because the key unavoidably includes references to colour and hair patterns that may be affected by age, wear or intraspecific variation, it should be used in conjuction with the remarks that accompany the detailed descriptions.

**Females**

**Table:** 

1	Forewing: hairs in 1^st^ medial cell and most other cells; metasomal terga T1–4 of most species with apical hair bands	**2**
–	Forewing: hairs absent in 1^st^ medial cell, in other cells hairs absent or restricted to radial, marginal, 1^st^ and 2^nd^ submarginal cells; metasomal terga T1–4 without apical hair bands	**subgenus Asaropoda**
2	Integument of paraocular areas black; fore and mid femora and tibiae with iridescent blue-green hairs (subgenus Notomegilla)	**3**
–	Integument of paraocular areas partly yellow, white or ivory; hair on fore and mid legs never iridescent (subgenus Zonamegilla)	**4**
3	Metasoma uniformly covered with green/bronze hair	***aeruginosa***
–	Metasoma with pale blue bands, often with orange tints	***chlorocyanea***
4	Scutal hair various but not bright orange; hind tibial scopa white with dark longitudinal streak below basitibial plate	**5**
–	Scutal hair orange; hind tibial scopa orange with at most very short brown mark below basitibial plate	**12**
5	Dark streak on hind tibial scopa < 0.5× length tibia	**6**
–	Dark streak on hind tibial scopa ≥ 0.5× length tibia	**9**
6	Thoracic hair appears grey due to mixed black and pale hairs; metasomal hair bands metallic blue	***walkeri***
–	Thoracic hair appears orange or pale brown; metasomal hair bands various	7
7	Metasomal hair bands matt orange, relatively wide (band on T2 about 0.4× width of disc); T5 with pale hair across full width (Fig. 26)	***adelaidae***
–	Metasomal hair bands iridescent, but becoming dull with age; T5 with pale hair medially	8
8	Metasomal hair bands blue; dark scopal streak 0.3–0.5× length hind tibia; T5 with broad band of pale hair bordering fimbria (Fig. 35)	***thorogoodi***
–	Metasomal hair bands yellowish; dark scopal streak 0.2–0.4× length hind tibia; T5 with medial patch of pale hair (Fig. 30)	***indistincta***
9	Metasomal hair bands matt orange, relatively wide (band T2 about 0.4× width of disc); T5 with pale hair across full width (Fig. 26); dark streak on scopa 0.5–0.7× length hind tibia	***adelaidae***
–	Metasomal bands iridescent aqua, not wide (band T2 about 0.3× width of disc); T5 with pale hair scattered and sometimes with a central line	**10**
11	Face marks yellow; T5 with scattered white hair and dense central line extending into prepygdial fimbria (Fig. 28)	***asserta***
–	Face marks ivory or pale yellow; T5 with scattered white hair but central line, if present, not extending into prepygdial fimbria	**11**
12	Pale hair pattern on T5 wide, narrower laterally (Fig. 32); T2–4 hair bands extending anterolaterally below the gradulus (Fig. 16)	***murrayensis***
–	White hair pattern on T5 usually weaker, frequently with only scattered white hairs near lateral margins (Fig. 34); T2–4 apical hair bands not extending anterolaterally below the gradulus (Fig. 18)	***pulchra***
13	Metasomal terga with bands of dense orange, scale-like hair on apical margins, simpler orange hair distributed openly elsewhere	**alpha**
–	Metasomal terga T1–T4 with pale hair only in apical bands	**13**
13	T5 medially with black hair only, some white hair at lateral margins (Fig. 29), metasomal hair bands electric blue	***cingulata***
–	T5 with pale hair medially	**14**
14	Metasomal hair bands metallic orange with green iridescence; prepygidial fimbria orange-brown; T5 with scattered orange hair and medial line of denser orange hair extending into fimbria (Fig. 33)	***paeninsulae***
–	Metasomal hair bands green, greenish-yellow or dull orange; T5 with broad patch of dispersed white hair (Fig. 31, 36)	**15**
15	Hind tibial scopa bright orange with at most slight darkening below basitibial plate; more than half hind basitarsus covered with orange hair	***viridicingulata***
–	Tibial scopa usually pale orange, with a short brown streak below basitibial plate; less than half hind basitarsus covered with orange hair	***karlba***

**Males**

**Table:** 

1	Forewing: hairs present in 1^st^ medial cell and most other cells; metasomal terga T1–5 of most species with apical hair bands	**2**
–	Forewing: hairs absent in 1^st^ medial cell, in other cells hairs absent or restricted to radial, marginal, 1^st^ and 2^nd^ submarginal cells; metasomal terga T1–5 without hair bands	**subgenus Asaropoda**
2	S6 gently convex (Fig. 20); apex S7 broadly triangular; legs with some iridescent hairs (subgenus Notomegilla)	**3**
–	S6 with broad depressions either side of midline (Fig. 21); apex S7 ovate; legs without iridescent hairs (subgenus Zonamegilla)	**4**
3	Metasoma uniformly covered with green/bronze hair	***aeruginosa***
–	Metasoma with pale blue bands, often with orange tints	***chlorocyanea***
4	Outer surface of hind tibia covered with white hair	**5**
–	Outer surface of hind tibia covered with orange hair	**11**
5	Clypeus and labrum ivory or pale yellow	**6**
–	Clypeus and labrum bright yellow	**7**
6	Paraocular areas without dark hairs; T2–4 hair bands broad, extending anterolaterally below the gradulus (Fig. 17)	***murrayensis***
–	Paraocular areas with dark hairs; T2–4 apical hair bands not extending anterolaterally below the gradulus (Fig. 19)	***pulchra***
7	Posterior margin of S5 with distinct patch of dark, branched hair	**8**
–	Posterior margin of S5 with at most an indistinct patch of branched hair	**9**
8	Lateral black marks on clypeus narrow, more than twice as long as wide; metasomal bands blue, narrow (band on T2 0.30× width of disc)	***asserta***
–	Lateral black marks on clypeus about twice as long as wide; metasomal bands blue, usually with yellow tinge, band on T2 about 0.35× width of disc	***adelaidae***
9	Thoracic hair appears grey due to mixed black and pale hairs; metasomal hair bands metallic blue	***walkeri***
–	Thoracic hair appears orange or pale brown; metasomal hair bands various	**10**
10	Metasomal bands metallic blue; S7 windows large (Fig. 48)	***thorogoodi***
–	Metasomal bands orange or green/orange; S7 windows small, apical projection truncated (Fig. 43)	***indistincta***
11	Metasomal terga with dense bands of orange, scale-like hair on apical margins and similar hair distributed openly on the rest of each tergum	***alpha***
–	Metasomal terga T1–T4 with coloured hair only in apical bands	**12**
12	Metasomal hair bands electric blue	***cingulata***
–	Metasomal hair bands green or orange or a combination of these colours	**13**
13	Entire outer surface of hind basitarsus covered with orange hair, metasomal hair bands orange, T6 with broad orange hair band; S7 windows absent (Fig. 46)	***paeninsulae***
–	Less than 30% of outer surface of hind basitarsus with pale hair	**14**
14	Thoracic hair usually bright ferruginous; S7 rounded apically (Fig. 49)	***viridicingulata***
–	Thoracic hair pale orange; S7 with small apical projection (Fig. 44)	***karlba***

**Figures 16–19.**
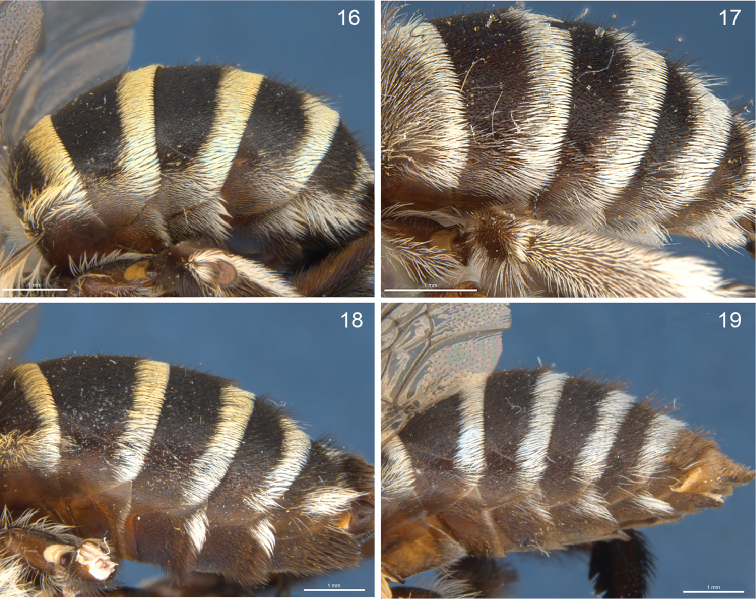
Abdomen, lateral view. **16** *Amegilla murrayensis* female **17** male **18** *Amegilla pulchra* female **19** male.

**Figure 20–21.**
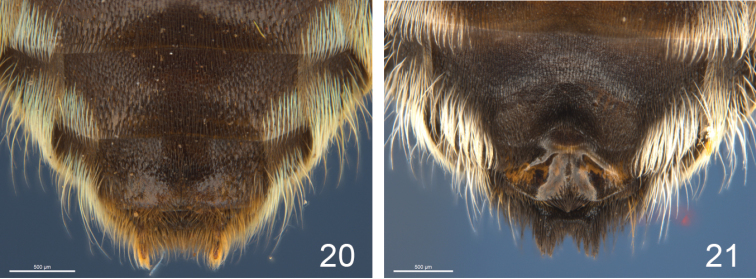
S6 of males **20** *Notomegilla* **21** *Zonamegilla*.

**Figure 22.**
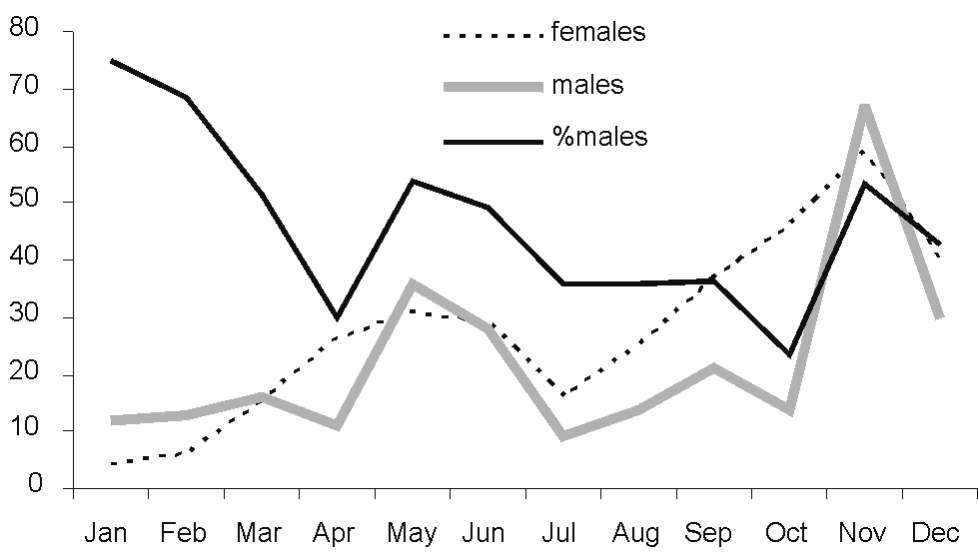
Phenology of *Amegilla aeruginosa*.

**Figure 23.**
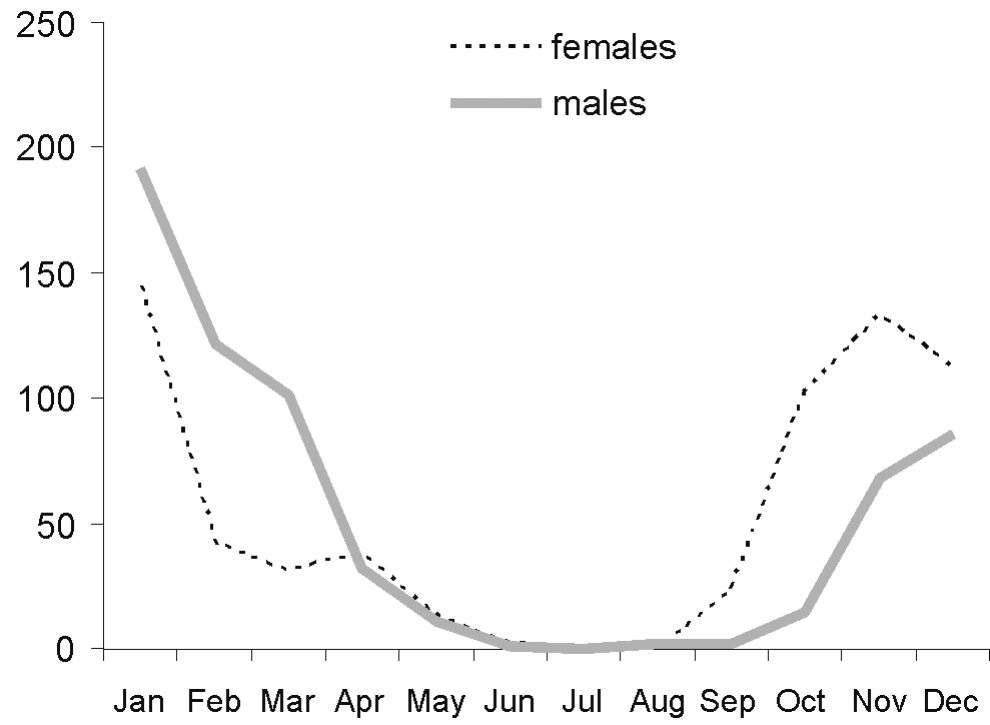
Phenology of *Amegilla chlorocyanea* south of 30°S.

**Figure 24–37.**
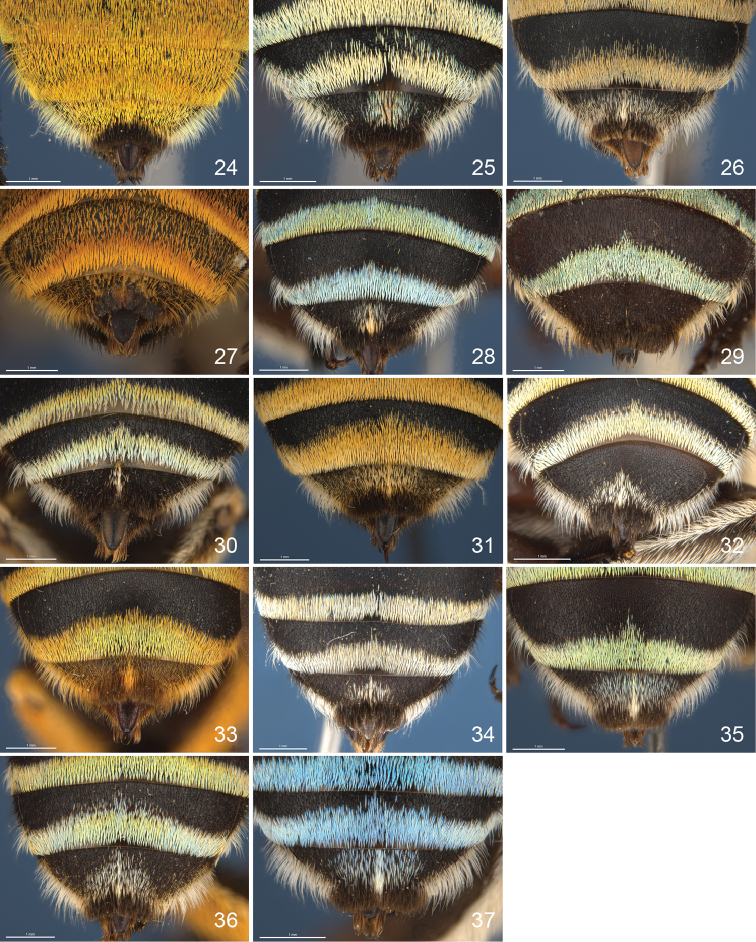
Apical tergites of females, especially T5. **24** *aeruginosa* **25** *chlorocyanea* **26** *adelaidae* **27** *alpha* **28** *asserta* **29** *cingulata* **30** *indistincta* **31** *karlba* **32** *murrayensis* **33** *paeninsulae* **34** *pulchra* **35** *thorogoodi* **36** *viridicingulata* **37** *walkeri*.

**Figures 38–50.**
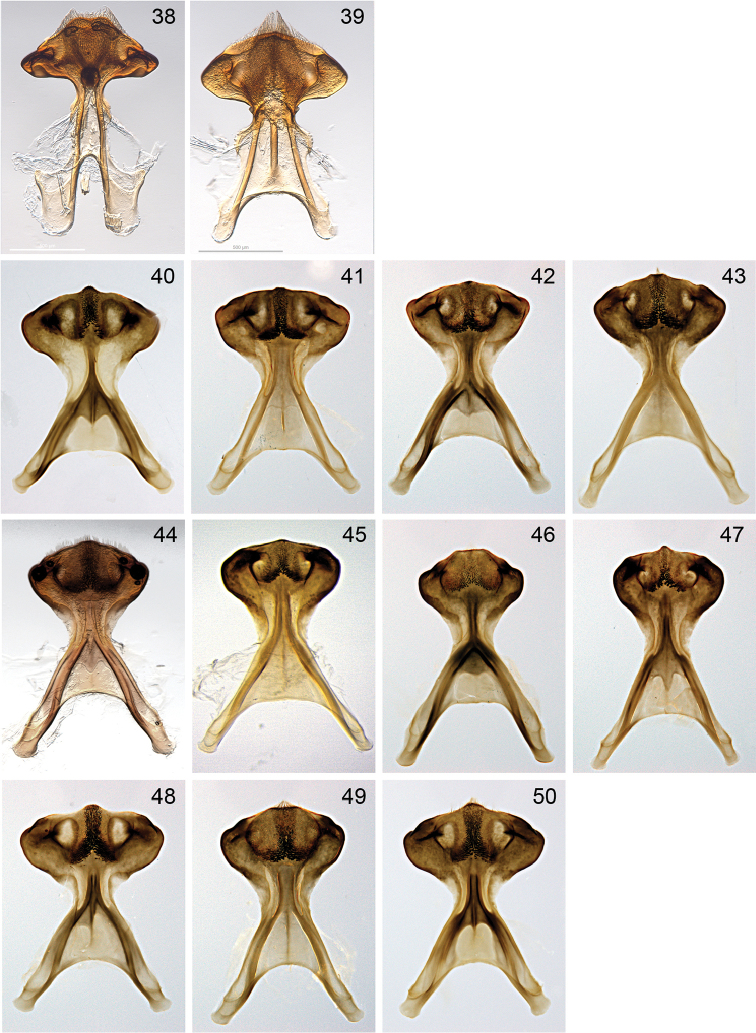
S7 of males. **38** *aeruginosa* **39** *chlorocyanea* **40** *adelaidae* **41** *asserta* **42** *cingulata* **43** *indistincta* **44** *karlba* **45** *murrayensis* **46** *paeninsulae* **47** *pulchra* **48** *thorogoodi* **49** *viridicingulata* **50** *walkeri*.

### Subgenus Asaropoda

Taxon classificationAnimaliaHymenopteraApidae

Cockerell, 1926.

Asaropoda Cockerell, 1926, p. 216. Type species: Saropoda bombiformis Smith, 1854 (original designation).

#### Diagnosis.

Length 13–24 mm; pubescence brown to grey (mostly black in *aurata* from New Guinea and the Bismark Archipelago); maxillary palpus with last segment fused to fifth segment so that maxillary palpus appears five-segmented.; apical margins of male metasomal sterna modified; S4 usually produced medially, rounded and having a thick brush of hair; S5 broadly and deeply emarginate; S6 shallowly emarginate medially with one or two patches of hair laterally; S7 somewhat quadrate, medioapically emarginate; S8 apically narrowed; apex of gonocoxite of male bilobed with long narrow upper lobe and small ventral lobe; gonostylus of male well developed. Forewing conspicuously hairy near costal margin, 1^st^ discoidal cell without hairs, other closed cells with hairs sparse or absent; gonostylus of male distinct, slender, and directed apically.

#### Distribution.

Widely distributed across Australia, New Guinea and the Bismark Archipelago, but not recorded from Tasmania.

#### Number of species in Australia.

Cardale (1993) listed 25 names of which one, *alpha*, is here moved to the subgenus Zonamegilla. An additional species, *paracalva*, was described by Brooks (1993). The species belonging to this subgenus are in need of revision.

### Subgenus Notomegilla

Taxon classificationAnimaliaHymenopteraApidae

Brooks, 1988

Notomegilla Brooks, 1988, p. 511.Type species: Anthophora aeruginosa Smith, 1854 (original designation).

#### Diagnosis.

Length 9–12 mm; metallic green/blue hair present on femur and tibia of fore and mid legs; pale paraocular marks present in males, absent in females; maxiliary palpus with 4 or 5 segments. Most or all closed cells of forewing with some rather long hairs; S6 of male simple, convex without lateral depression, edge entire to shallowly emarginate medially; apex of S7 of male greatly expanded laterally, without subapical circular area; apex of S8 narrowed, truncate or rounded.

#### Description.

For a full description of the subgenus see Brooks (1988).

***Female. Structure.*** Head: wider than long; inner orbits of eyes diverging above; length f3–9 subequal. ***Coloration*.** Integument black, except ivory or pale yellow marks on labrum, mandibles, clypeus and supraclypeal area (Brooks 1988 erroneously stated that paraocular marks are well developed in both sexes.) ***Pubescence*.** Head: labrum white; gena with white hairs. Legs: forecoxa white; hind basitibia white with black streak. Metasoma: T1–T4 with apical hair bands, T5 with long white hair laterally, S3-S4 dark medially, white with metallic blue-green iridescence posterolaterally, S5 dark. ***Punctation*.** Head: interspaces on clypeus pit-reticulate; labrum somewhat shiny, with close punctation, interspaces smooth. Scutum with small, shallow punctures; interspaces almost smooth.

***Male. Structure.*** Head: wider than long; inner orbits diverging above; length f3–10 subequal. Wings: length cu-v of hind wing subequal to second abscissa M+Cu. Metasoma: Apicomedial margin T7 bilobed. ***Coloration*.** Integument black, pale yellow marks on labrum, mandibles, clypeus, scape, paraocular and supraclypeal areas. ***Pubescence*.** Head: labrum white, gena mostly white. Legs: Forefemur posteriorly with long, light coloured hair; forecoxa white; mid and hind legs dark or black, with lighter coloured hairs on apex of femur, outer surface of tibia and basitarsus. Metasoma: T1–T6 with apical hair bands or entirely covered with adpressed hair; S6 dark. ***Sculpture*.** Head: clypeus dull with small, shallow, open punctures; interspaces pit-reticulate; Labrum somewhat shiny, with small, shallow punctures; interspaces interspaces pit-reticulate. Thorax: scutum with medium, strong, dense punctures; interspaces smooth. Metasoma: T1–T5 with shallow, close, somewhat scrobiculate punctures; interspaces pit-reticulate.

#### Distribution.

Australia.

#### Included species.

*aeruginosa* and *chlorocyanea*.

### Amegilla (Notomegilla) aeruginosa

Taxon classificationAnimaliaHymenopteraApidae

(Smith)

Figs 4 , 24 , 38

Anthophora aeruginosa Smith, 1854, p. 336.
Amegilla aeruginosa (Smith) Michener, 1965, p. 216.
Amegilla (Notomegilla) aeruginosa (Smith) Brooks, 1988, p. 512.
Anthophora kershawi Rayment, 1944, p. 21, **n. syn.**
Anthophora sybilae Rayment, 1944, p. 22, **n. syn.**
Amegilla sybilae (Rayment) Michener, 1965, p. 217.
Amegilla (Notomegilla) sybilae (Rayment) Brooks, 1988, p. 512.

#### Material examined.

364 females and 286 males.

#### Types.

Lectotype of *aeruginosa*: male, BMNH 17B.659. The original description was from one or more male and female syntypes, reportedly collected from Hunter River, Australia. The only specimen found that was considered to be one of the syntypes was a single male bearing no collection information, but two handwritten labels: “aeruginosa Type Sm.” and “Anthophora aeruginosa Type Sm.”. Accordingly this specimen is here designated as the lectotype.

Holotype (by monotypy) of *kershawi*: male, Claudie River, Queensland, ANIC 32-033849.

Syntypes of *sybilae*: male, Macintosh Holding, Queensland, 14 Mar.1940,”cotype”, ANIC 32-034555; female, Magnetic Island, Queensland, 20 Dec. 1939, “cotype”, ANIC 32-034554; female, Edungalba, Queensland, 4 Nov.1939, ANIC 32-033280; female, Litchfield, Daly R., NT, T.G. Campbell, 5/4/1929, ANIC 32-033879.

#### Decision for synonymy.

Based on examination of the holotype and of Rayment’s syntypes, combined with results of DNA analyses of specimens from across its geographical range we conclude that there is evidence for only one species. Uncorrected sequence divergence was found to be 0–2.0%, which is within the limits expected for conspecific individuals (Hebert et al. 2010).

#### Diagnosis.

Both sexes are easily distinguished from other Australian *Amegilla* by the green/bronze metallic pubescence which covers most of the dorsal surface.

#### Redescription.

*Female*: Pine Creek, NT, 10 Jul. 1997, Leg. R. Leijs SAMA 32-002560.

Length 10 mm; forewing length 9 mm.

***Structure.*** Head: clypeus protuberant, in profile 0.4× width of eye; galea in repose reaching more than half-way between coxa of fore and mid legs; length f1 3× length f2; IOD 1.4× OOD; OS 0.6× OOD. ***Coloration*.** Yellow marks on labrum, mandibles, supraclypeal areas and inverted T-shape on clypeus; paraocular areas and scape black. ***Pubescence*.** Head: labrum and clypeus white, a light yellow hair patch in centre of paraocular area, some black hairs between antennae, near ocelli and vertex, white hair with blue-green metallic iridescence in remaining areas; gena white with metallic blue-green hair. Thorax: scutum with mixed dark and yellowish hair with a metallic blue-green iridescence; pleura pale yellow below wing base, white with metallic blue-green iridescence in other areas; thoracic sterna white with metallic blue-green iridescence; propodeum laterally, dark intermixed longer whitish hair. Legs: fore outer femur and tibia posteriorly with white hair; outer tibia and basitarsus with white hair with metallic blue-green iridescence, rest of tarsus dark; mid legs predominantly dark, white on apex of femur, white with metallic blue-green iridescence on outer surface of tibia and as a basal patch on basitarsus; hind legs predominantly dark, except white on apex of femur and white with metallic blue-green iridescence on outer surface of tibia plus a small basal patch on basitarsus; basitibial streak 0.5× length of femur. Metasoma: T2–T5: green or bronze with a metallic iridescence; T5 laterally with white hair (Fig. 24); fimbria black. ***Punctation*.** Head: clypeus dull, with close small, shallow punctures, 0.2–1.0 puncture widths apart; labrum with medium, shallow punctures, 0.2–1.0 puncture widths apart, interspaces smooth. Thorax: scutum weakly shining with dense punctures 0.2–1.0 puncture widths apart. Metasoma: T1–T5 shining with open to close, small, shallow punctures 0.5–2.0 puncture widths apart, interspaces transverse pit-reticulate.

*Male*: Pine Creek, NT, 10 July 1997, Leg. R. Leijs SAMA 32-002559.

Length 10 mm; forewing length 8 mm.

***Structure.*** Head: shortest distance between eyes 0.8× length of eye; clypeus protuberant, in profile 0.6× width of eye; galea in repose reaching more than halfway between fore and mid coxae; length f1 1.4× length f2, 0.7× length scape (excluding basal bulb) and 0.7× length f10; length f3–10 1.4× width; IOD 1.8× OOD; OS 0.9× OOD. Wings: length of marginal cell 0.8× distance from apex of marginal cell to wing tip; length of vein M of hind wing 2.5× length second abscissa of M+Cu; length of jugal lobe about 0.6× length of vannal lobe. Metasoma: apicomedial emargination of S5 weak; S7 windows absent, median hair brush very wide at apex and narrowing towards anterior end (Fig. 38). ***Pubescence*.** Head: white on labrum, becoming light yellow towards top of head with some black hairs between antennae, near ocelli and on vertex; ocellocular areas and frons white with blue-green metallic iridescence; gena white with metallic blue-green dense hair. Thorax: scutum black intermixed with white hair with metallic blue-green iridescence; pleura white with metallic blue-green iridescence; thoracic sterna white with metallic blue-green iridescence; propodeum laterally white with metallic blue-green iridescence. Legs: fore outer femur and tibia posteriorly with long white hair; outer tibia and basitarsus with metallic blue-green hair, rest of tarsus dark; mid legs dark, except long white hair posteriorly on femur and tibia, and white hair with metallic blue-green iridescence on apex of femur, outer surface of tibia and posterior edge of basitarsus; hind legs dark, except white with metallic blue-green iridescence on apex of femur and outer surface of tibia. Metasoma: apical hair bands on T1–T6 orange with metallic green iridescence, parts not covered by hair bands bronze with a metallic green iridescence on basal two thirds; T6,T7 brownish-black; S2–S5 dark except apical margins laterally with white hair bands with metallic blue-green iridescence. ***Punctation*.** Head: clypeus punctures 1.0–2.0 puncture widths apart; labrum: punctures 1.0–2.0 puncture widths apart. Thorax: Thorax: scutum somewhat shiny, with close punctures, 0.3–2.0 puncture widths apart. Metasoma: T1–T5 with small, shallow punctures, 0.5–1.5 puncture widths apart.

#### Variation.

The colour of the metasomal hair is predominantly green for approximately half the specimens and bronze for the remaining half, although intermediate coloration is found in a few individuals.

#### Phenology.

**Table:** 

Month:	Jan	Feb	Mar	Apr	May	Jun	Jul	Aug	Sep	Oct	Nov	Dec
**No. of records**:	16	19	31	37	67	55	25	39	58	60	126	70

*Amegilla aeruginosa* was observed throughout the year in the Northern parts of Australia. There were an increase in numbers at the start of the wet season, and a decrease in January and February. This may be due to a slowing down or lack of reproduction in the wettest part of the year. The strong male bias in the first two months of the year (Fig. 22) may be a consequence of protandry.

#### Distribution.

Widely distributed in the tropics and subtropics, with little overlap with the distribution of Amegilla (Notomegilla) chlorocyanea (Fig. 4).

### Amegilla (Notomegilla) chlorocyanea

Taxon classificationAnimaliaHymenopteraApidae

(Cockerell)

Figs 7 , 25 , 39

Anthophora chlorocyanea Cockerell, 1914, p. 469.
Amegilla chlorocyanea (Cockerell) Michener, 1965, p. 216.
Amegilla (Notomegilla) chlorocyanea (Cockerell) Brooks, 1988, p. 512.
Anthophora australis Rayment, 1944, p. 24, **n. syn.**
Amegilla australis (Rayment) Michener, 1965, p. 216.
Amegilla (Zonamegilla) australis (Rayment) Brooks, 1988, p. 511.
Anthophora adamsella Rayment, 1944, p. 23, **n. syn.**
Amegilla adamsella (Rayment) Michener, 1965, p. 216.
Amegilla (Notomegilla) adamsella (Rayment, 1944) Brooks, 1988, p. 512.
Anthophora ferrisi Rayment, 1947, p. 73, **n. syn.**
Amegilla ferrisi (Rayment) Michener, 1965, p. 216.
Amegilla (Zonamegilla) ferrisi (Rayment) Brooks, 1988, p. 511.
Anthophora grayella Rayment, 1944, p. 27, **n. syn.**
Amegilla grayella (Rayment) Michener, 1965, p. 216.
Amegilla (Notomegilla) grayella (Rayment) Brooks, 1988, p. 512.
Anthophora tinsleyella jamesi Rayment, 1944, p. 30, **n. syn.**
Amegilla jamesi (Rayment) Michener, 1965, p. 216.
Amegilla (Notomegilla) jamesi (Rayment) Brooks, 1988, p. 512.
Anthophora luteola Rayment 1944, p. 27.
Amegilla luteola (Rayment) Michener, 1965, p. 217.
Anthophora mewiella Rayment, 1944, p. 28, **n. syn.**
Amegilla mewiella (Rayment) Michener, 1965, p. 217.
Amegilla (Notomegilla) mewiella (Rayment) Brooks, 1988, p. 512.
Anthophora luteola murrayi Rayment, 1944, p. 28, **n. syn.**
Amegilla murrayi (Rayment) Michener, 1965, p. 217.
Amegilla (Notomegilla) murrayi (Rayment) Brooks, 1988, p. 512.
Anthophora tinsleyella Rayment 1944, p. 29.
Amegilla tinsleyella (Rayment) Michener, 1965, p. 217.

#### Material examined.

1110 females and 946 males.

#### Type data.

Holotype of *chlorocyanea*: female, whereabouts unknown. As the original description is unambiguous, no neotype is required.

Syntype of *australis*: female, Sandringham, Victoria, 7 Nov. 1936, on *Dianella revoluta*, ANIC 32-034544.

Syntype of *adamsella*: male, Edungalba, Queensland, May 1940, 5EEA, “Allotype”, ANIC 32-034545.

Syntypes of *grayella*: male, female, Orroroo, South Australia, 20 Feb. 1940 No. 88, ANIC 32-034546,7.

Syntypes of *tinsleyellajamesi*: male, female, Orroroo, South Australia, 20 Feb. 1940, 92, ANIC 32-034553,4.

Syntypes of *luteola*: male, female, Orroroo, South Australia, 3 Mar. 1939, “Gray 23 Adelaide”, ANIC 32-034548,9.

Syntypes of *mewiella*: male, Broken Hill, 20 Feb. 1940, ANIC 32-033124; 2 males and female, Orroroo, South Australia, 20 Feb. 1940, ANIC 32-033133,32-0334550,1.

Syntype (presumed) of *luteolamurrayi*: female, Robertson, New South Wales, Feb 1940, ANIC 32-033076. (Specimen bears no type or identification label.)

Syntypes (presumed) of *tinsleyella*: 5 males, Orroroo, SA, 4 Feb 1940, 20 Feb 1940 (3), 26 Feb 1940, ANIC 32-033182, 32-033192, 32-033194, 32-033196, 32-033396.

No type material was found for *ferrisi* but it is placed in *chlorocyanea* on the basis of the description provided by Rayment (1947).

#### Decisions for synonymy.

The results of DNA analyses of specimens from across the complete geographical range indicated that there is only one banded *Notomegilla* species. Uncorrected sequence divergence was found to be 0–3.05%, which is below the usual limits for conspecific individuals. Moreover, there was no geographical pattern in the sequence variation. No morphological differences, other than colour variation, were found when Rayment’s syntypes were examined. Variations in the genitalia as illustrated by Rayment (1944) appear to be due to different focal distances and angles of the drawings.

We agree with Brooks’s (1988) decision to synonymise *luteola* and *tinsleyella* with *chlorocyanea*. Both Sichel (1869) and Cockerell (1905) have incorrectly referred to this species as *Anthophora cingulata* (Fabr.)

#### Diagnosis.

This species superficially resembles several *Zonamegilla* species, but can be distinguished by the blue/green iridescent pubescence on the fore and mid legs; females by the completely black paraocular areas and a large, dense, medial spot of pale pubescence on T5 (Fig. 25); and males by a smooth unmodified surface of S5 and medially interrupted hair bands on T4–T6.

#### Redescription.

*Female*: Sunnyside, 11km NW of Murray Bridge, 35.0536S 139.3620E, SA, 28 Dec 2003, R.Leijs & K.Hogendoorn, SAMA 32-002545.

Length 13 mm; forewing length 9.5 mm.

***Structure.*** Head: clypeus protuberant, in profile 0.5× width of eye; galea in repose reaching just past forecoxa; length f1 3.5× length f2, 0.9× length scape (excluding basal bulb) and 2× length f10; f3–9 as long as wide; IOD 1.6× OOD; OS 0.8× OOD. ***Coloration*.** Pale yellow marks on labrum, mandibles, supraclypeal areas and inverted T-shape on clypeus; paraocular areas and scape black. ***Pubescence*.** Head: labrum white, remaining areas predominantly pale yellow with some black hairs between antennae and on vertex; gena white/pale brown. Thorax: scutum ginger intermixed with black hairs; pleura light ginger under wing base, remainder white; thoracic sterna white; propodeum laterally light ginger intermixed with black. Legs: foreleg brown, except white long hair posteriorly on femur and pale hair with orange or light blue metallic iridescence on outer tibia and basitarsus; mid legs black, except pale hair with orange or light blue metallic iridescence on apex of femur, outer tibia and basal part of basitarsus; hind legs black except pale with orange or light blue metallic iridescence on apex of femur, outer tibia and basal part of basitarsus; length of basitibial streak 0.4× length of femur. Metasoma: apical hair bands on T1–T4 white with metallic blue-green iridescence, margin of T4 medially with hairless shiny triangle, parts not covered by hair bands black, with some white hair medially on T4; T5 laterally with long white hair intermixed with hairs with metallic blue-green iridescence (Fig. 25), fimbria black, distinct, round, medial patch of white hair with metallic blue-green iridescence. ***Punctation*.** Head: clypeus somewhat shiny, with medium, close, shallow punctures, 0.25–1.0 puncture widths apart; labrum large, shallow punctures, 0.2–0.8 puncture widths apart, interspaces smooth. Thorax: scutum shiny, with close punctation, 0.3–1.0 puncture widths apart. Metasoma: T1–T5 with somewhat shiny, close to open, fine, shallow punctures, 0.5–2.0 puncture widths apart, interspaces pit-reticulate.

*Male*: Sunnyside, 11km NW of Murray Bridge, 35.0536S 139.36199E, SA, 28 Dec 2003, R.Leijs & K.Hogendoorn, SAMA 32-002542.

Length 11 mm; forewing length 9 mm.

***Structure.*** Head: shortest distance between eyes 0.6× length of eye; clypeus protuberant, in profile 0.4× width of eye; galea in repose almost reaching mid coxa; length f1 2.4× length f2, 0.7× length scape (excluding basal bulb) and 1.1× length f11; length f3–10 1.2× width; IOD 1.8× OOD; OS 0.9× OOD. Wings: length of marginal cell 0.7× distance from apex of marginal cell to wing tip; length of vein M of hind wing 2.3× length second abscissa of M+Cu; length of jugal lobe about 0.4× length of vannal lobe. Metasoma: apicomedial emargination of S5 weak and broad; S7 windows absent or very small (Fig. 39); S7 median hair brush very short with long lateral wings in v-shape. ***Pubescence*.** Head: labrum white, remaining areas predominantly pale yellow with some black hairs along lateral margins of clypeus, between antennae and on vertex; gena white. Thorax: scutum brown intermixed with black hair; pleura light brown under wing base, remainder white; propodeum laterally light brown. Legs: forefemur white; fore outer tibia and basitarsus with light blue iridescence; mid legs black, except long white hair posteriorly on femur and tibia and white hair with weak metallic orange or light blue iridescence on apex of femur, outer surface of tibia and base of basitarsus; hind legs black, except white with weak metallic orange or light blue iridescence on apex of femur and outer surface of tibia. Metasoma: T1–T6: apical hair bands on margin greyish white with weak metallic blue-green iridescence, bands on T4–6 black medially and therefore seeming interrupted; parts not covered by hair bands black; T7 brown; S2–S5 black. ***Punctation*.** Head: clypeus punctures 1.5–3.0 puncture widths apart; labrum punctures 1.5–3.0 puncture widths apart. Thorax: scutum shiny, with close punctures, 0.5–1.0 puncture widths apart. Metasoma: T1–T5 with fine, shallow punctures, 1.0–2.0 puncture widths apart.

#### Variation.

About 10% of specimens have enough orange pigmentation to make the tergal bands and scutal hair orange and the hair of the legs pale orange. A larger number of specimens have almost white tergal bands, presumably as a consequence of wear.

#### Phenology.

**Table:** 

Month:	Jan	Feb	Mar	Apr	May	Jun	Jul	Aug	Sep	Oct	Nov	Dec
**No of records Nof 30°S**:	30	18	41	51	77	6	3	26	74	90	72	57
**No of records S of 30°S**:	338	165	133	70	24	6	0	2	25	125	205	202

*Amegilla chlorocyanea* occurs throughout Australia, but the epicentre of the distribution is in the south of the continent, as is demonstrated by the fact that 73% of the specimens with known localities (n = 2043) have been collected south of a latitude of 30°S. The phenology changes with the latitude: peak activity is in January in the south, and in May and October in the north. In Fig. 23 the frequency of males and females is given only for specimens caught south of 30°S.

#### Distribution.

Wide-spread throughout the arid and temperate areas of the southern part of mainland Australia and Tasmania (Fig. 7).

### Subgenus Zonamegilla

Taxon classificationAnimaliaHymenopteraApidae

Popov, 1950

Zonamegilla Popov, 1950, p. 260.

#### Type.

*Apis zonata* Linnaeus, 1758 (original designation).

#### Diagnosis.

Length 10–14 mm; most species with blue, green, white, or occasionally orange, metallic hair bands on metasomal terga; pale paraocular markings present in both sexes; maxiliary palpus with 6 segments; S5 of male apicomedially broadly to narrowly emarginate; S6 of male with lateral depressions on apical third, sometimes with a median protuberance, tuft of black hair apicomedially.

#### Description.

The following description refers to the Australian species of *Zonamegilla* (see Brooks 1988 for a full description of the subgenus).

***Female. Structure.*** Head: wider than long; inner orbits diverging above; f3–9 about equal in length. ***Coloration*.** Integument black, except yellow or pale yellow marks on labrum, mandibles, clypeus, scape, paraocular and supraclypeal area (marks are ivory in *pulchra*). ***Pubescence*.** Head: gena with white hairs. Legs: forecoxa and femur posteriorly light ginger in *paeninsulae*, white in all other species, hair of anterior face of femur and tibia white or ginger; mid and hind legs dark or black, but lighter coloured on apex of femur, on outer surface of tibia and on base of basitarsus. Metasoma: apical hair bands on T1–T4; parts not covered by hair bands dark brown or black in *Amegilla alpha*, black in all other species. ***Punctation*.** Head: clypeus somewhat shiny, close to dense punctation, interspaces pit-reticulate; labrum interspaces almost smooth in *murrayensis* and *adelaidae*, reticulate in other species. Thorax: scutum with small, shallow punctures; interspaces almost smooth. Metasoma: T1–T5 somewhat shiny, with fine, shallow punctures, interspaces pit-reticulate.

***Male. Structure.*** Head: wider than long; inner orbits diverging above; f3–10 about equal in length. Metasoma: apicomedial margin of T7 bilobed. ***Coloration*.** Integument black, except yellow or pale yellow marks on labrum, mandibles, clypeus, scape, paraocular and supraclypeal area (marks are ivory in *pulchra*). ***Pubescence*.** Head: gena with white hair. Thorax: sterna pale orange in *paeninsulae*, white in all other species. Legs: forefemur posteriorly with long, light coloured hairs; coxa greyish white in *paeninsulae*, white in all other species; mid and hind legs: dark or black, with lighter coloured hairs on apex of femur, outer surface of tibia and basitarsus. Metasoma: apical hair bands on T1–T5; parts not covered by hair bands dark brown in *walkeri*, black in all other species. S6 dark except in *paeninsulae*, *thorogoodi* and *cingulata*. ***Punctation*.** Head: clypeus dull, with open shallow punctures, interspaces rough pit-reticulate in *karlba*, pit-reticulate in all other species; labrum somewhat shiny, interspaces pit-reticulate; scutum: interspaces almost smooth. Metasoma: interspaces pit-reticulate.

#### Distribution.

India, South East Asia including southern China, and Australia.

#### Included Australian species.

*adelaidae*, *alpha*, *asserta*, *cingulata*, *thorogoodi*, *indistincta*, *karlba*, *murrayensis*, *paeninsulae*, *pulchra*, *viridicingulata* and *walkeri*.

### Amegilla (Zonamegilla) adelaidae

Taxon classificationAnimaliaHymenopteraApidae

(Cockerell)

Figs 3 , 26 , 40

Anthophora adelaidae Cockerell, 1905, p. 397.
Amegilla adelaidae (Cockerell) Michener, 1965, p. 216.
Amegilla (Zonamegilla) adelaidae (Cockerell) Brooks, 1988, p. 511.

#### Material examined.

54 females and 33 males.

#### Type data.

Holotype of *adelaidae*: male, Adelaide River, NT, BMNH 17B.664.

The identity of the species *adelaidae* was determined unequivocally from the shape of S7 of the holotype.

#### Diagnosis.

*Amegilla adelaidae* may be recognized by the matt, pale orange tergal hair bands in both sexes; females by the broad, entire patch of white hairs on T5 (Fig. 26); males by the shape of S7, which has a smoothly rounded posterior margin with a sharp apical projection, a narrow ventral ridge and narrow Y-shaped brush with an acute angle between the lateral arms (Fig. 40).

#### Redescription.

*Female*: Berrimah, Research Farm Orchard, NT, 12.4333S 130.9167E, 14 May 2003, G.R. Brown & H. Wallace, DNA voucher RB266 (RL501), SAMA-32-002617.

Length 13 mm; forewing length 8 mm.

***Structure.*** Head: clypeus protuberant, in profile 0.5× width of eye; galea in repose reaching beyond mid coxa; length of f1 2.7× length of f2, 0.8× length of scape (excluding basal bulb) and 1.8× length of f10; length of f3–9 0.9× width; IOD 1.3× OOD; OS 0.6× OOD. ***Coloration*.** Yellow marks on labrum, mandible, scape, clypeus, paraocular and supraclypeal areas; inverted T-shape on clypeus; f2 and apex of f1 orange ventrally. ***Pubescence*.** Head: labrum white, remaining areas predominantly pale, darker towards the vertex; black robust hairs scattered between antennae, near ocelli and on vertex, a few on clypeus; gena white, light brown towards vertex. Thorax: scutum ginger intermixed with black hair; pleura ginger with scattered black hairs under wing base, white ventrally; thoracic sterna white; propodeum laterally light ginger with scattered black hairs. Legs: forefemur posteriorly with long white hair, outer surface of foretibia and -tarsus pale yellow, inner surface of foretarsus dark; mid legs dark, except white hairs on apex of femur and on outer surface of tibia and basitarsus and a streak of contiguous short white hairs on posterior proximal part of femur; hind legs dark, except white hairs on apex of femur and outer surface of tibia, white patch on basal part of basitarsus; basitibial streak black, 0.7× length of femur. Metasoma: apical hair bands on T1–T4 white with weak light blue and orange iridescence; T5 laterally with long white hairs and few dispersed short hairs (Fig. 26), fimbria dark, medial patch forming broad band around fimbria, medial streak overlapping fimbria; S3, S4 dark, posterolateral patches of white hairs; S5 dark, laterally with small white patches. ***Punctation*.** Head: clypeus with close medium sized, deep punctures, 0.1–0.8 puncture widths apart; labrum shiny, with close, small punctures of intermediate depth, 0.2–0.9 puncture widths apart, interspaces almost smooth. Thorax: scutum somewhat shiny, with close punctures, 0.2–0.8 puncture widths apart. Metasoma: T1–T5 with close punctures, 0.8–1.5 puncture widths apart.

*Male*: Berrimah, Research Farm Orchard, NT, 130.9167E 12.4333S, 14 May 2003, G.R. Brown & H. Wallace, DNA voucher RB266 (RL502), SAMA-32-002616.

Length 12 mm; forewing length 8.5 mm.

***Structure.*** Head: shortest distance between eyes 0.8× length of eye; clypeus protuberant, in profile 0.5× width of eye; galea in repose reaching beyond mid coxa; length of f1 1.9× length of f2, 0.6× length of scape (excluding basal bulb) and 1.1× length of f11; length of f3–10 1.1× width; IOD 1.4× OOD; OS 0.7× OOD. Wings: length of marginal cell 0.8× distance from apex of marginal cell to wing tip; length of vein M of hind wing 2.3× length of second abscissa of M+Cu; length of jugal lobe about 0.5× length of vannal lobe. Metasoma: apicomedial emargination of S5 wide; S7 with rounded apical margin, sharp apical projection, large windows, width medial ridge 1.6× length, narrow Y-shaped brush (Fig. 40); S8 apical emargination wide almost trilobed. ***Pubescence*.** Head: labrum white, remaining areas predominantly pale, darker towards the vertex; scattered black robust hairs on clypeus, paraocular areas, between antennae, near ocelli and on vertex; gena white. Thorax: scutum light brown intermixed with black hairs; pleura light brown with scattered black hairs under wing base, white ventrally; propodeum laterally light brown with scattered black hairs. Legs: forefemur posteriorly with long white hair, outer surface of tibia and tarsus pale yellow, inner surface of tarsus dark; mid legs dark, except white hair on the apex of femur, posteriorly on proximal half of femur and on outer surface of tibia and basitarsus; hind legs dark, except white hairs on apex of femur and outer surface of tibia, small white patch on outer base of basitarsus. Metasoma: apical hair bands on T1–T5 greyish white with orange tinge, almost not iridescent; T6, T7 black when viewed from behind, brown when viewed from the side; S2–S5 medially dark, lateral thirds white. ***Punctation*.** Head: clypeus with punctures 0.5–3.0 puncture widths apart; labrum with small, shallow punctures 0.5–2.0 puncture widths apart. Thorax: scutum shiny, with close, small, shallow punctures 0.5–1.0 puncture widths apart, interspaces smooth. Metasoma: T1–T5 shiny, with open, fine, shallow punctures, 1.0–3.0 puncture widths apart.

#### Variation.

Most female specimens have pale orange bands with no metallic reflections, but the bands of males are frequently paler and more often show hints of green iridescence. A few specimens, male and female, had ivory, rather than yellow face marks.

#### Phenology.

**Table:** 

Month:	Jan	Feb	Mar	Apr	May	Jun	Jul	Aug	Sep	Oct	Nov	Dec
**No. of records**:	4	4	7	13	32	2	0	3	3	0	2	6

#### Distribution.

Australia, mainly in tropical and subtropical areas, including the arid zone (Fig. 3).

### Amegilla (Zonamegilla) alpha

Taxon classificationAnimaliaHymenopteraApidae

(Cockerell)

Figs 5 , 27

Sarapoda bombiformis var *α* Smith, 1854, p. 318.
Saropoda alpha Cockerell, 1904, p. 204.
Amegilla (Asaropoda) alpha (Cockerell) Michener, 1965, p. 217.

#### Material examined.

5 females and 1 male.

#### Type data.

Holotype of *alpha*: male, (no locality data), BMNH 17B.669.

#### Diagnosis.

*Amegilla alpha* is easily distinguished from all other Australian *Zonamegilla* species by the orange hair covering the dorsal surface of the metasoma in both sexes, superficially resembling that in *Asaropoda* species. The yellow paraocular marks and facial profile of females, the unmodified apical margins of S4 and S5 of males, and for both sexes the presence of hairs in 1^st^ medial cell and most other cells of the forewing, separate it from *Asaropoda*.

#### Redescription.

*Female*: Jasper Gorge 54km NW Victoria River Downs, 16.02S 130.41E, NT, 30 Apr. 1974, T.Weir & T. Angeles, MAGNT I004904.

Length 14 mm; forewing length 9 mm.

***Structure***. Head: clypeus protuberant, in profile 0.5× width of eye; galea in repose reaching just reaching mid coxa; length of f1 3× length of f2, 0.8× length of scape (excluding basal bulb) and 1.7× length of f10; f3–9 as long as wide; IOD 1.3× OOD; OS 0.5× OOD; length of marginal cell 0.8× distance from apex of marginal cell to wing tip; cu-v of hind wing approximately half length of second abscissa of M+Cu; length of vein M of hind wing 1.7 times as long as second abscissa of M+Cu; length of jugal lobe about 0.5× length of vannal lobe. ***Coloration*.** Yellow marks on labrum, mandibles, scape, clypeus, paraocular and supraclypeal areas; inverted T-shape on clypeus; distal part of flagellum brown ventrally from apex of f1. ***Pubescence*.** Head: labrum and clypeus with orange setae, pale yellow hairs intermixed with dark hairs on paraocular areas, frons, near ocelli and on vertex, darker near ocelli; gena white, pale ginger towards vertex. Thorax: scutum orange intermixed with black hairs; pleura orange with few black hairs under wing base, white ventrally; thoracic sterna orange; propodeum laterally orange with few black hairs. Legs: forefemur posteriorly with long white hair, outer surface of foretibia and -tarsus light orange, inner surface of tarsus brown; mid leg black, except light orange hair on apex of femur and on outer surface of tibia and basitarsus, hair on basitarsus lighter than on tibia; posterior proximal part of femur with a narrow line of light orange hair; hind legs black, except orange hair on apex of femur, scopa and basal part of basitarsus; basitibial streak brown, very short. Metasoma: apical hair bands on T1–T4 ginger with weak orange iridescence, parts not covered by hair bands dark orange; T5 laterally pale yellow (Fig. 27), fimbria brown, medial patch absent; pubescence as on T1–4; S3, S4 dark, posterolateral patches of pale yellow hairs; S5 dark. ***Punctation*.** Head: clypeus with dense to close, large, deep punctures, 0.1–0.5 puncture widths apart; labrum somewhat shiny, with close to dense, medium, deep punctures, 0.1–0.5 puncture widths apart. Thorax: scutum shiny, with close punctures, 0.2–1.0 puncture widths apart. Metasoma: T1–T5 with close to open punctures, 0.5–1.5 puncture widths apart.

*Male*: Holotype.

Length 11 mm; forewing length 7.5 mm.

***Structure.*** Head: clypeus protuberant, in profile 0.4× width of eye; length f1 2.1× length f2, 0.6× length scape (excluding basal bulb); length f3–10 1.1× width; IOD 1.4× OOD; OS 0.7× OOD. Metasoma: apicomedial emargination of S5 wide, 40% of the sternal width, S6 with lateral depressions. ***Pubescence*.** Head: labrum white, remaining areas predominantly pale, darker towards vertex; scattered black robust hairs on clypeus laterally and in pale paraocular areas; gena white. Thorax: scutum orange-brown intermixed with black hairs; pleura light brown, white ventrally; propodeum laterally orange-brown with scattered black hairs. Legs: forefemur posteriorly with long white hairs, outer surface of tibia, tarsus orange-brown, inner surface of tarsus dark; mid legs black, except orange-brown hair on apex of femur, outer surface of tibia and basitarsus; hind legs black, except orange-brown hair on apex of femur, outer face of tibia and base of basitarsus. Metasoma: apical hair bands on T1–T5 orange-brown, elsewhere hair simple, open, orange-brown; S3–S5 medially dark, laterally orange.

#### Phenology.

**Table:** 

Month:	Jan	Feb	Mar	Apr	May	Jun	Jul	Aug	Sep	Oct	Nov	Dec
**No. of records**:	0	0	0	3	2	0	0	0	0	0	0	0

#### Distribution.

(Fig. 5). The locality data given by Rayment (1951) as Toowoomba and Mackay does not fit the distribution as we know it. Since Rayment did not examine the type of *alpha*, the basis of his statement about the distribution is unclear.

### Amegilla (Zonamegilla) asserta

Taxon classificationAnimaliaHymenopteraApidae

(Cockerell)

Figs 6 , 28 , 41

Anthophora asserta Cockerell, 1926, p. 224.
Amegilla asserta (Cockerell) Michener, 1965, p. 216.
Amegilla (Zonamegilla) asserta (Cockerell) Brooks, 1988, p. 511.
Anthophora perasserta assertiella Rayment, 1947, p. 63, **n. syn.**
Amegilla assertiella (Rayment) Michener, 1965, p. 216.
Anthophora longmani Rayment, 1947, p. 21, **n. syn.**
Amegilla longmani (Rayment) Michener, 1965, p. 217.
Amegilla (Zonamegilla) longmani (Rayment) Brooks, 1988, p. 511.
Anthophora perasserta Rayment, 1947, p. 62, **n. syn.**
Amegilla perasserta (Rayment) Michener, 1965, p. 217.
Amegilla (Zonamegilla) perasserta (Rayment) Brooks, 1988, p. 511.
Anthophora perpulchra Rayment, 1947, p. 64, **n. syn.**
Amegilla perpulchra (Rayment) Michener, 1965, p. 217.
Amegilla (Zonamegilla) perpulchra (Rayment) Brooks, 1988, p. 511.
Anthophora whiteleyella Rayment, 1947, p. 72, **n. syn.**
Amegilla whiteleyella (Rayment) Michener, 1965, p. 217.
Amegilla (Zonamegilla) whiteleyella (Rayment) Brooks, 1988, p. 511.

#### Material examined.

253 females and 215 males.

#### Type data.

Holotype of *asserta*: male, Lower Ferntree Gully, 22.1.1916, VIC, MV, T-11865.

Lectotype of *assertiella*: male, Cooranbong, NSW, 20 May 1939, ANIC 32-034245, here designated.

Syntype of *longmani*: male, Bribie Is., Queensland, “allotype”, ANIC 32-034572.

Syntypes of *perasserta*: male, female, Clermont, QLD, K.K. Spence, AM K.105230, K.105227; male, female, Edungalba, Qld, 5 Nov. 1940, ANIC 32-033329, 32-033332; 2 males and female, White Swamp, NSW, ANIC 32-033341, 32-034566, 32-033514; female, Magnetic Is., Qld, ANIC 32-034078; male, Orroroo, SA, 1 Mar. 1940, “J.T.G.23”, ANIC 32-033362; female, Gatton, QLD, N.C. Lloyd, 20 Dec. 1937, ASCU; female, Glen Innes, NSW, 20 Mar. 1914, ASCU.

Syntypes of *perpulchra*: male, female, Mittagong, NSW, 2 Feb. 1940, “TYPE”and “Allotype”, ANIC 32-034564; female, Port Hacking, NSW, T.G Campbell, 30–31 Jan. 1925, AM K.55811; female, Port Hacking, NSW, T.G Campbell, 12 Mar. 1927, AM K.60206; female, Bendermeer, NSW, D.A. Porter, 28 Feb. 1926, AM K.53522; male, Sydney, NSW, C. Gibbons, AM K.48991; female, Brisbane, QLD, AM K.15779; male, female, Robertson, NSW, 9 Mar. 1940, ANIC 32-034309, 32-034533; 2 males, Edungalba, Qld, , 2 Nov. 1940, “No E30”, ANIC 32-034314,5; male, Gosford, NSW, 10 Mar. 1940, “103”, ANIC, 32-034323; female, Ingham, Qld, 27 Dec. 1940, “EEA 2”, ANIC 32-034321; female, Cooper Park, Sydney, NSW, “No 18 NS”, ANIC 32-034301; female, Lismore, NSW, Mrs Higgison, 10 Jan. 1934, ASCU; female, Glen Innes, NSW, 20 Mar. 1914, ASCU; female, Liverpool, NSW, 30 Mar. 1909, ASCU.

Holotype of *whiteleyella* (Rayment, 1947) (by presumed monotypy), male, Macquarie River, NSW, Nov. 1935, ANIC 32-033369.

#### Decisions for synonymy.

Examination of the above type material revealed no morphological differences.

#### Diagnosis.

*Amegilla asserta* is distinguished from other Australian *Zonamegilla* species by a combination of the following characters: Face marks yellow; tergal hair bands pale blue. Hind tibia of females with long dark basitibial streak; T5 with dense medial patch of pale hair including a central line extending into prepygidial fimbra (Fig. 28). Posterior margin of S5 of males with a distinct patch of branched hairs; S7 with a narrow medial ridge (Fig. 41).

#### Redescription.

*Female*: Sydney Botanical gardens, 33.850S 151.200E, NSW, 31 Mar 2003, M. Bell, DNA voucher RB081 (RL490), SAMA 32-002567.

Length 13 mm; forewing length 9 mm.

***Structure.*** Head: clypeus protuberant, in profile 0.4× width of eye; galea in repose reaching half-way between coxa of fore and mid legs; length of f1 3× length of f2, 0.8× length of scape (excluding basal bulb) and 1.7× length of f10; length of f3–9 0.9× width; IOD 1.3× OOD; OS 0.7× OOD. ***Coloration*.** Pale yellow-yellow marks on labrum, mandibles, scape, clypeus, paraocular and supraclypeal areas; inverted T-shape on clypeus. ***Pubescence***. Head: labrum white, remaining areas predominantly pale brown with scattered black robust hairs on clypeus, paraocular areas, between antennae, near ocelli and on vertex; gena white, pale orange towards vertex. Thorax: scutum dark ginger intermixed with black hairs; pleura ginger with scattered black hairs under wing base, white ventrally; thoracic sterna white; propodeum laterally pale ginger with scattered black hairs. Legs: forefemur posteriorly with long white hair, outer surface of tibia and tarsus with white hair, inner surface of tarsus brown; mid legs dark, with whitish hair on apex of femur, posteriorly on proximal third of femur, on outer surface of tibia and forming a small basal patch posteriorly on basitarsus; hind legs black, except white hair on apex of femur and outer surface of tibia; basitibial streak black, 0.7× length of femur. Metasoma: apical hair bands on T1–T4 white with metallic blue iridescence; T5 laterally with long white hairs intermixed with short hairs, fimbria dark, medial patch as in Fig. 28; S3, S4 dark, except posterolateral patches of white hair; S5 dark brown, laterally with small white patches. ***Punctation*.** Head: clypeus with close, medium sized, shallow punctures, 0.2–1.0 puncture widths apart; labrum somewhat shiny, with close, small, shallow punctures, 1.0–2.0 puncture widths apart. Thorax: scutum shiny, with close punctures, 0.2–0.8 puncture widths apart. Metasoma: T1–T5 with close to open punctures, 0.5–2.0 puncture widths apart.

*Male*: S. of Coen, 14.0424S 143.19888E, Qld, R. Leijs & M. Batley, DNA voucher RB277 (RL777), SAMA 32-002572.

Length 11 mm; forewing length 8 mm.

***Structure***. Head: shortest distance between eyes 0.7 length of eye; clypeus protuberant, in profile 0.5 width of eye; galea in repose almost reaching mid coxa; length of f1 2× length of f2, 0.7× length of scape (excluding basal bulb) and 1.1× length of f12; length of f3–10 1.3× width; IOD 1.2× OOD; OS 0.6× OOD. Wings: length of marginal cell 0.8× distance from apex of marginal cell to wing tip; length of vein M of hind wing 2 times as long as second abscissa of M+Cu; length of jugal lobe about 0.3× length of vannal lobe. Metasoma: apicomedial emargination of S5 narrow; S7 windows medium size, half circular; S7 median hair brush 3× as long as wide; S7 lateral wings of median hair brush well developed an angle of ≥90° between them (Fig. 41) ; S8 apical emargination shallow. ***Pubescence***. Head: labrum white, remaining areas predominantly pale brown with scattered black robust hairs on clypeus, paraocular areas, between antennae, near ocelli and on vertex; gena white, pale orange towards vertex. Thorax: scutum brown intermixed with black hairs; pleura brown with scattered black hairs under wing base, white ventrally; propodeum laterally brown with scattered black hairs. Legs: forefemur posteriorly with long white hairs, outer surface of tibia and tarsus with pale brown hairs, inner surface of tarsus brown; mid legs dark, except pale yellow hairs on the apex of the femur, posteriorly on proximal one third of femur and on outer surface of tibia and basitarsus; hind legs black, except pale yellow hairs on apex of femur and outer surface of tibia. Metasoma: apical hair bands on T1–T5 white with metallic blue iridescence; T6, T7 black when viewed from behind, light brown when viewed from the side; S2–S5 dark with white apicolateral patches. ***Punctation***. Head: clypeus punctures 1.0–3.0 puncture widths apart; labrum with small, shallow punctures 0.5–1.5 puncture widths apart. Thorax: scutum somewhat shiny, with close medium, shallow punctures 0.5–1.0 puncture widths apart. Metasoma: T1–T5 shiny, with open small, shallow punctures, 1.0–2.5 puncture widths apart.

#### Variation.

The black areas on the clypeus of males are consistently narrow, usually 4 or 5 times as long as wide, but occasionally the length is only 2.5 times the width. The colour of the tergal bands in both sexes usually displays distinct blue iridescence and appears to be less susceptible to the effects of aging than in species like *pulchra*. The scutal hair of both sexes is usually distinctly ginger in appearance. The colour of the flagellum varies in both sexes from dull orange-brown to dark brown on the ventral surface and from dark brown to black on the dorsal surface. Very rarely, the length of the dark streak in the hair of the hind tibia of females is 0.5× the length of the tibia.

#### Remarks.

*Amegilla asserta* superficially resembles *thorogoodi* and *indistincta*, but females may be distinguished by the longer hind basitibial streak the hair pattern on T5 (Fig. 28). Males may be distinguished from both species by the patch of branched hairs on S5 and from *indistincta* by the shape of S7 (Fig. 41).

#### Phenology.

**Table:** 

Month:	Jan	Feb	Mar	Apr	May	Jun	Jul	Aug	Sep	Oct	Nov	Dec
**No of records Nof 30°S**:	36	30	20	18	20	12	8	2	5	11	39	23
**No of records S of 30°S**:	55	31	37	11	3	0	0	0	0	0	12	22

*Amegilla asserta* is one of the most common and widespread species along the eastern seaboard, reaching from the tip of Cape York into South Australia. In the south of the continent, the species is active between November and April, with a peak in January. In the north, *Amegilla asserta* can be found year round, albeit at lower frequencies in July, August and September.

#### Distribution.

From Eyre Peninsula and the Lofty Ranges in South Australia, Tasmania, and the temperate areas of Victoria and New South Wales to subtropical and tropical areas along the east coast of Queensland (Fig. 6).

### Amegilla (Zonamegilla) cingulata

Taxon classificationAnimaliaHymenopteraApidae

(Fabricius)

Figs 8 , 29 , 42

Andrena cingulata Fabricius, 1775, p. 378.
Amegilla cingulata (Fabricius) Michener, 1965, p. 216.
Amegilla (Zonamegilla) cingulata (Fabricius) Brooks, 1988, p. 511.
Anthophora emendata Smith, 1879, p. 123. **n. syn.**
Amegilla emendata (Smith) Michener, 1965, p. 216.
Anthophora emendata gilberti Cockerell, 1905, p. 396. **n. syn.**
Amegilla gilberti (Cockerell) Michener, 1965, p. 216.
Anthophora lilacine Cockerell, 1921, p. 84. **n. syn.**
Amegilla lilacine (Cockerell) Michener, 1965, p. 216.
Anthophora fabriciana Rayment, 1947, p. 53 **n. syn.**
Amegilla fabriciana (Rayment) Michener, 1965, p. 216.
Amegilla (Zonamegilla) fabriciana (Rayment) Brooks, 1988, p. 511.

#### Material examined.

186 females and 148 males.

#### Type data.

Holotype of *cingulata*, female, BMNH, ‘Australia’, Banks Collection, E-668712. Mr D. Notton informed us that it is stored in the Banks Collection over the cabinet label ‘Andrena cingulata Fabr. Sp. Ins. No. 17’.

Holotype of *emendata* (by monotypy), male, BMNH, 17B.448.

Syntype of *emendatagilberti*, female, QLD, BMNH, 17B.665.

Holotype of *lilacine*, male, Kuranda, QLD, QM Hy/2497.

Holotype of *fabriciana* (by monotypy), female, “ No 31; In a tunnelled cell in plaster in the walls of an old house”, “Anthophora cincta, Dours”, ANIC 32-034445.

#### Discussions for synonymy.

Based on examination of the type material, we concur with Meade-Waldo’s (1914) decision to synonimise *emendata* and *gilberti* and Brooks’s (1988) decision to synonymise *lilacine*. Meade-Waldo (1914) suggested that Smith’s (1879) description of the type of *emmendata* as a female is a typographical error. Although the holotype of *fabriciana* does not have that name attached, it is the only specimen found bearing the name “Anthophora cincta, Dours” and the other attached information is consistent with Rayment’s references to “wattle-and-daub walls” (Rayment 1935), “a remarkably large female from a plaster cell. Maitland” (as *Anthophora gilberti* in Rayment 1939), which became “Dours’s bee … from the Hunter River” (Rayment 1941). Tergites 5 and 6, and the corresponding sternites, were missing from the specimen, presumably because they were mislaid after being used in the production of Fig III, 2 in Rayment (1941). Despite repeated references by Rayment to a length of 18 mm, our estimate is that the length of the intact specimen would have been 15 mm. All observable features of the specimen are consistent with its belonging to *Amegilla cingulata*.

#### Diagnosis.

*Amegilla cingulata* is a distinctive species with metallic blue tergal hair bands and orange scutal pubescence in both sexes. Females lack a dark basitibial dark streak on the hind legs and the disc of T5 is without pale hair (Fig. 29).

#### Redescription.

*Female*: Levers Plateau, 28.33S 152.88E, Qld, 13 Mar 1966, T. F. Houston, WAM 5461.

Length 14 mm; forewing length 9 mm.

***Structure.*** Head: clypeus protuberant, in profile 0.3× width of eye; galea in repose reaching mid coxa; length of f1 2.9× length of f2, 0.8× length of scape (excluding basal bulb) and 1.5× length of f10; f3–9 as long as wide; IOD 1.1× OOD; OS 0.5× OOD. ***Coloration*.** Yellow marks on labrum, mandibles, scape, clypeus, paraocular and supraclypeal areas; inverted T-shape on clypeus; distal part of flagellum brown ventrally from f2. ***Pubescence*.** Head: labrum white, remaining areas white to light ginger, darker towards vertex and intermixed with black hairs on clypeus, paraocular areas, frons, near ocelli and on vertex; gena white, light ginger towards vertex. Thorax: scutum orange intermixed with black hairs; pleura orange with few black hairs under wing base, white ventrally; thoracic sterna white; propodeum laterally ginger with few black hairs. Legs: forefemur posteriorly with long white hairs, outer surface of fore tibia and -tarsus light ginger, inner surface of tarsus brown; mid legs black, except light ginger hairs on apex of femur and on outer surface of tibia and basitarsus, hairs on basitarsus lighter than on tibia; posterior proximal part of femur with a narrow line of light ginger hairs; hind legs black, except ginger hairs on apex of femur, scopa and basal part of basitarsus, a white tuft on apex of tibia; basitibial streak absent. Metasoma: apical hair bands on T1–T4 white with electric blue iridescence; T5 laterally white (Fig. 29), fimbria brown, medial patch absent; S3, S4 brown, posterolateral patches of white hairs; S5 brown. ***Punctation*.** Head: clypeus with close medium to large, deep punctures, 0.2–1.0 puncture widths apart; labrum somewhat shiny, with close, medium, deep punctures, 0.2–0.6 puncture widths apart. Thorax: scutum shiny, with close punctures, 0.2–0.8 puncture widths apart. Metasoma: T1–T5 with close to open punctures, 0.5–1.0 puncture widths apart.

*Male*: Bombana National Park, 27.47S 153.02E, Qld, 16 Mar 1966, T. F. Houston, WAM 5462.

Length 13 mm; forewing length 8 mm.

***Structure***. Head: shortest distance between eyes 0.8 length of eye; clypeus protuberant, in profile 0.5 width of eye; galea in repose reaching mid coxa; length of f1 1.3× length of f2, 0.5× length of scape (excluding basal bulb) and 0.8× length of f11; length of f3–10 1.2× width; IOD 1.2× OOD; OS 0.5× OOD. Wings: length of marginal cell 0.9× distance from apex of marginal cell to wing tip; vein M of hind wing 2.3 times as long as second abscissa of M+Cu; length of jugal lobe about 0.5× length of vannal lobe. Metasoma: apicomedial emargination of S5 intermediate width and depth; S7 windows medium size, median hair brush 4× width, lateral wings of hair brush narrow almost perpendicular to the long axis (Fig. 42); S8 apical emargination deep. ***Pubescence***. *Head*: labrum white; clypeus and paraocular marks predominantly black, remainder ginger with scattered black hairs on frons, near ocelli and on vertex; gena white, light ginger towards vertex. Thorax: scutum orange intermixed with black hairs; pleura orange under wing base, turning white ventrally; thoracic sterna and propodeum laterally orange. Legs: forefemur posteriorly with long white hairs, outer surface of tibia and tarsus pale yellow, inner surface of tarsus brown; mid legs brown, except pale ginger hairs on apex of femur, posteriorly proximally on femur and on outer surface of tibia and basitarsus; hind legs black, except orange hairs on apex of femur and outer surface of tibia and a very small tuft on base of basitarsus, apex of tibia white. Metasoma: apical hair bands on T1–T5 white with electric blue iridescence; T6–T7 black when viewed from behind, brown when viewed from side; T7 black; S2–S5 pale brown with white lateral patches; S6 black when viewed from behind, brown when viewed from side. ***Punctation***. Head: clypeus punctures 1.0–3.0 puncture widths apart; labrum with medium, shallow punctures 0.5–2.0 puncture widths apart. Thorax: scutum somewhat shiny with close to open, medium, shallow punctures 0.2–2.0 puncture widths apart. Metasoma: T1–T5 somewhat shiny, with close, fine, shallow punctures, 0.8–1.5 puncture widths apart.

#### Variation.

The thoracic hair, especially of females, is consistently bright orange, but the metasomal bands are seldom infused with orange and even then, the colour is mostly restricted to the band on T1. The colour of the flagellum is variable, as in *asserta*. Two females were found with a few white hairs on T5 forming the narrowest of longitudinal lines.

#### Phenology.

**Table:** 

Month:	Jan	Feb	Mar	Apr	May	Jun	Jul	Aug	Sep	Oct	Nov	Dec
**No. of records**:	29	23	29	54	33	39	16	17	8	15	14	23

#### Distribution.

Along the east coast of New South Wales and Queensland (Fig. 8).

### Amegilla (Zonamegilla) indistincta sp. n.

Taxon classificationAnimaliaHymenopteraApidae

http://zoobank.org/E1938D51-F866-4DE9-BB1D-46F6EE46EA28

Figs 9 , 30 , 43

#### Material examined.

40 females and 27 males.

#### Type data.

Holotype: female: Millstream Falls, 17.6427S 145.4588E, 4 Jul 2007, R. Leijs & M. Batley, DNA voucher RB312 (RL867), SAMA 32-002623.

Allotype: male, Iron Range, QLD, 1 Jul 2007, 12.7465S 143.2556E, R.Leijs & M. Batley, SAMA 32-002633.

Paratypes: male, female, 23 km SW of Agnes Water, QLD, 24.3500S 151.9333E, 31 Jan 2007, M. Batley, DNA vouchers RB194, RB195, AM K-290886, AM K-290887.

#### Diagnosis.

*Amegilla indistincta* is distinguished from other Australian *Zonamegilla* species by the following combination of characters: Tergal hair bands usually with a yellowish tint. Hind tibia of females with a short dark streak (≤ 0.4× length hind tibia); pale hair on T5 forming a relatively small medial patch (Fig. 30). Apical margin of T5 of males lacking a patch of dense, dark hair; medial ridge of S7 very broad and apical projection truncate (Fig. 43).

#### Description.

*Female*: holotype.

Length 14 mm; forewing length 9 mm.

***Structure.*** Head: clypeus protuberant, in profile 0.4× width of eye; galea in repose just reaching mid coxa; length of f1 3× length of f2, 0.9× length of scape (excluding basal bulb) and 1.6× length of f10; length of f3–9 0.9× width; IOD 1.3× OOD; OS 0.6× OOD; length of marginal cell 0.8× distance from apex of marginal cell to wing tip; length of cu-v of hind wing approximately half the length of second abscissa of M+Cu; length of vein M of hind wing 2.2× length of second abscissa of M+Cu; length of jugal lobe about 0.5× length of vannal lobe. ***Coloration*.** Yellow marks on labrum, mandibles, scape, clypeus, paraocular and supraclypeal areas; inverted T-shape on clypeus; small brown spot on f2. ***Pubescence*.** Head: labrum and clypeus white, paraocular areas and frons white intermixed with black hairs, light ginger intermixed with black hairs near ocelli and on vertex; gena white, light ginger towards vertex. Thorax: scutum light orange intermixed with black hair; pleura light orange with few black hairs under wing base, white ventrally; thoracic sterna white; propodeum laterally light orange with few black hairs. Legs: forefemur posteriorly with long white hair, outer surface of tibia and tarsus greyish white, inner surface of tarsus brown; mid legs dark, except white hair on apex of femur and on outer surface of tibia and basal part of basitarsus; apex of tibia with brown spot; posterior proximal part of femur with narrow line of white hair; hind legs black, except greyish white hair on apex of femur, scopa, white hair on basal part of basitarsus and on apex of tibia; basitibial streak black, 0.4× length of femur. Metasoma: apical hair bands on T1–T4 greyish white with green-blue iridescence; T5 laterally white, fimbria dark brown, medial patch of dispersed short white hairs around a denser longitudinal line (Fig. 30); S3, S4 dark with posterolateral patches of white hair; S5 dark. ***Punctation*.** Head: clypeus with dense, large, deep punctures, 0.1–0.5 puncture widths apart; labrum with somewhat shiny, with close, medium sized, deep punctures, 0.1–0.8 puncture widths apart. Thorax: scutum shiny, with close punctures, 0.2–0.7 puncture widths apart. Metasoma: T1–T5 with close punctures, 0.8–1.5 puncture widths apart.

*Male*: allotype.

Length 12 mm; forewing length 9 mm.

***Structure.*** Head: shortest distance between eyes 0.5× length of eye; clypeus protuberant, in profile 0.6× width of eye; galea in repose reaching mid coxa; length of f1 1.5× length of f2, 0.6× length of scape (excluding basal bulb) and 0.8× length of f1; f3–10 as long as wide; IOD 1.3× OOD; OS 0.6× OOD. Wings: length of marginal cell 0.8× distance from apex of marginal cell to wing tip; length of vein M of hind wing 2.1× length of second abscissa of M+Cu; length of jugal lobe about 0.4× length of vannal lobe. Metasoma: apicomedial emargination of S5 moderately wide and shallow; S7 with wide medial ridge and truncate apical projection, hair pattern almost inverted T-shaped, apical half weak (Fig. 43); S8 apical emargination deep. ***Pubescence*.** Head: labrum white, remaining areas predominantly ginger, some black hairs on clypeus, paraocular areas, frons, near ocelli and on vertex; gena white, ginger towards vertex. Thorax: scutum ginger intermixed with black hair; pleura ginger, white ventrally; propodeum laterally light ginger. Legs: forefemur posteriorly with long white hair, outer surface of tibia and tarsus light ginger, inner surface of tarsus brown; mid legs black, except white hair on apex of femur, posteriorly on proximal end of femur and on outer surface of tibia and basitarsus; hind legs black, except ginger hair on apex of femur and outer surface of tibia, apex of tibia white. Metasoma: apical hair bands on T1–T5 light ginger; T6 with few long white hairs on apical margin, remaining hair black when viewed from behind, brown when viewed from the side; S2–S5 medially dark, laterally with white patches; S6 dark. ***Punctation*.** Head: clypeus punctures 0.5–3.0 puncture widths apart; labrum with small, shallow punctures 0.5–2.0 puncture widths apart. Thorax: scutum shiny, with close small, shallow punctures, 0.2–1.0 puncture widths apart. Metasoma: T1–T5 somewhat shiny, with open, fine, shallow punctures, 0.8–2.0 puncture widths apart.

#### Phenology.

**Table:** 

Month:	Jan	Feb	Mar	Apr	May	Jun	Jul	Aug	Sep	Oct	Nov	Dec
**No. of records**:	5	4	6	10	6	4	3	0	9	9	5	4

#### Distribution.

In the subtropics and tropics along the east coast of Queensland (Fig. 9).

#### Etymology.

The specific epithet refers to the fact that specimens of this species were found among the type series of other species described by Rayment (*indistincta* in Latin means ’not distinguished’).

#### Remarks.

*Amegilla indistincta* is closely related to *karlba* and superficially resembles *thorogoodi* and *asserta*. Females may be distinguished from *asserta* by the length of the hind tibial streak and from *karlba* and *thorogoodi* by the relatively small patch of pale hair on T5. Males may be distinguished from *asserta* by the absence of a hair patchon S5, and from *thorogoodi* by the shape of S7 (Fig. 43). While the tergal hair bands of female *indistincta* are less blue than those of *thorogoodi*, this colour difference may prove unreliable for separating the species.

Males of *indistincta* are not easily distinguishable from those of *karlba*, but the small number of specimens available all had a truncate apical projection on S7 (Fig. 43), while the projection is blunt but rounded in *karlba* (Fig. 44).

### Amegilla (Zonamegilla) karlba sp. n.

Taxon classificationAnimaliaHymenopteraApidae

http://zoobank.org/F5CAC773-BD97-4ECC-86A7-A8E10E5A3F0F

Figs 10 , 31 , 44

#### Material examined.

28 females and 19 males.

#### Type data.

Holotype: female, 12km NNW of Mt Cahill, NT, 12.46S 132.39E, 20 Jun 1973, T. Weir & T. Angeles, MAGNT I004902.

Allotype: male, 19km NE by E of Mt Cahill, NT, 12.47S 132.51E, 16.xi.1972, T. Weir & A. Allwood, MAGNT I004897.

Paratypes: male, 16km E by N of Mt Cahill, NT, 12.8333S 132.8500E, 16.xi.1972, T. Weir & A. Allwood, MAGNT I004895; 2 females, 19km E by N of Mt Cahill, NT, 12.5000S 132.5200E, 14 Jun 1973, J.C. Cardale, ANIC 32-33723, 33725; female, male, 19km E by N of Mt Cahill, NT, 12.5000S 132.5200E, 16 Nov 1972, J.C. Cardale, ANIC 32-33757, 34386; 4 males, 19km NE by E of Mt Cahill, NT, 12.47S 132.51E, 16.xi.1972, T. Weir & A. Allwood, MAGNT I004894, I004896, I004898, I004899; female, Litchfield NP, NT, 13.1167S 130.7833E, G. Williams & W. Pulawski, AM K-290928, DNA voucher RB330.

#### Diagnosis.

*Amegilla karlba* is distinguished from other Australian *Zonamegilla* species by the following characters: Metasomal hair bands of both sexes yellow ochre coloured; hair on outer face of the hind tibia usually orange or brown. Basitibial hair streak on hind leg of females short; hind basitarsus less than half covered with pale hair; T5 with pale hair reaching lateral margins. S7 of males with a broad medial ridge resulting in small, lightly pigmented windows and a broad but distinct apical projection (Fig. 44).

#### Description.

*Female*: holotype.

Length 13 mm; forewing length 9 mm.

***Structure.*** Head: clypeus protuberant, in profile 0.3× width of eye; galea in repose reaching mid coxa; length of f1 3.1× length of f2, 0.7× length of scape (excluding basal bulb) and 1.6× length of f10; f3–9 as long as wide; IOD 1.2× OOD; OS 0.6× OOD; length of marginal cell 0.8× distance from apex of marginal cell to wing tip; cu-v of hind wing 2.7× length of second abscissa of M+Cu; length of vein M of hind wing 2× length of second abscissa of M+Cu; length of jugal lobe about 2× length of vannal lobe. ***Coloration*.** Yellow marks on labrum, mandibles, scape, clypeus, paraocular and supraclypeal areas; inverted T-shape on clypeus; distal part of flagellum orange-brown ventrally from f2. ***Pubescence*.** Head: labrum white, remaining areas predominantly pale yellow, darker towards vertex; scattered black robust hairs on clypeus, paraocular areas, frons, near ocelli and on vertex; gena white, pale yellow towards vertex. Thorax: scutum ginger intermixed with black hair; pleura ginger under wing base, turning white ventrally; thoracic sterna pale brown; propodeum laterally ginger with scattered black hairs. Legs: forefemur posteriorly with long white hair, outer surface of foretibia and -tarsus light brown, inner surface of tarsus brown; mid legs dark, except pale brown hair on apex of femur and outer surface of tibia, slightly darker than on foretibia, posteriorly proximal end of femur with narrow line of white hair, apex of tibia with brown spot, basitarsus white basally; hind legs black, except pale brown hair on apex of femur and outer surface of tibia, a patch of pale brown hair on base of basitarsus, small white tuft on apex of tibia; basitibial streak brown, 0.25× length of femur. Metasoma: apical hair bands on T1–T4 yellow ochre with orange and weak light blue iridescence; T5 laterally with moderately dense white hairs, fimbria brown; T5 entirely covered with open pale yellow ochre hairs, a denser longitudinal line extends into fimbria (Fig. 31); S3, S4 dark, posterolateral patches of pale yellow hairs; S5 dark. ***Punctation*.** Head: clypeus with dense to close, medium sized, deep punctures, 0.2–0.7 puncture widths apart; labrum somewhat shiny, with close to dense, small, deep punctures, 0.1–0.5 puncture widths apart. Thorax: scutum shiny, with close punctures, 0.2–1.5 puncture widths apart. Metasoma: T1–T5 with close punctures 0.2–0.8, puncture widths apart.

*Male*: allotype.

Length 11 mm; forewing length 8 mm.

***Structure.*** Head: shortest distance between eyes 0.7× length of eye; clypeus protuberant, in profile 0.6× width of eye; galea in repose reaching mid coxa; length of f1 1.8× length of f2, 0.5× length of scape (excluding basal bulb) and 0.9× length of f11; length of f3–10 1.3× width; IOD 1.4× OOD; OS 0.7× OOD. Wings: length of marginal cell 0.8× distance from apex of marginal cell to wing tip; length of vein M of hind wing 2.2× length of second abscissa of M+Cu; length of jugal lobe about 0.4× length of vannal lobe. Metasoma: apicomedial emargination of S5 very shallow, moderately wide; S7 with very broad medial ridge leaving small, lightly pigmented windows, apical projection very broad, but distinct and not truncate, hair pattern almost inverted T-shaped (Fig. 44); S8 apical emargination deep. ***Pubescence*.** Head: labrum white, remaining areas predominantly pale yellow, darker towards vertex; scattered black robust hairs on clypeus, paraocular areas, frons, near ocelli and on vertex; gena white, pale yellow towards the vertex. Thorax: scutum ginger intermixed with black hair; pleura ginger under wing base, becoming white ventrally; propodeum laterally ginger with scattered black hairs. Legs: forefemur posteriorly with long white hair, outer surface of tibia and tarsus pale brown, inner surface of tarsus brown; mid legs dark, except pale brown hair on apex of femur, posteriorly on proximal end of femur and on outer surface of tibia and basitarsus; hind legs black, except ginger hair on apex of femur and outer surface of tibia, a few light ginger hairs on base of basitarsus, apex of tibia white. Metasoma: apical hair bands on T1–T5 yellow ochre with orange and weak light blue iridescence; T6 yellow ochre hairs on apical margin, remaining hair black when viewed from behind, brown when viewed from the side; S2–S5 brown with narrow white lateral patches. ***Punctation*.** Head: clypeus with punctures 1.0–3.0 puncture widths apart, interspaces rough pit-reticulate; labrum with small, shallow punctures, 0.5–2.0 puncture widths apart. Thorax: scutum somewhat shiny, close to open, medium, shallow punctures 0.2–2.0 puncture widths apart. Metasoma: T1–T5 somewhat shiny, with close, fine, shallow punctures, 0.5–1.5 puncture widths apart.

#### Phenology.

**Table:** 

Month:	Jan	Feb	Mar	Apr	May	Jun	Jul	Aug	Sep	Oct	Nov	Dec
**No. of records**:	2	3	0	5	5	13	1	0	0	2	13	1

#### Distribution.

Arnhem Land, Kakadu, Kimberleys (Fig. 10).

#### Etymology.

The specific epithet is a noun in apposition referring to the colour of the tergal hair bands (*karlba* in the language of the Kuninjku people of Western Arnhemland means yellow ochre (Evans et al. 2002)).

#### Remarks.

*Amegilla karlba* is similar to *indistincta* and, to a lesser extent, *viridicingulata*. Females may be distinguished from *viridicingulata* by the extent of pale hair on the hind basitarsus and from *indistincta* the more extensive area of white hair on T5 (Fig. 31). Hair on the hind tibia of female *karlba* is usually more orange than that in *indistincta*. Males of *karlba* may be distinguished by the shape of S7, differing from *adelaidae* and *walkeri* in the width of the medial ridge and from *viridicingulata* by the presence of an apical projection. Colour variation may make it difficult to distinguish males from those of *indistincta*, though the species are probably allopatric. Truncation of the apical projection of S7 may prove to be diagnostic for *indistincta* but examination of a greater number of specimens is needed.

### Amegilla (Zonamegilla) murrayensis

Taxon classificationAnimaliaHymenopteraApidae

(Rayment)

Figs 11 , 32 , 45

Anthophora murrayensis Rayment, 1939, p. 288.
Amegilla murrayensis (Rayment) Michener, 1965, p. 217.
Amegilla (Zonamegilla) murrayensis (Rayment) Brooks, 1988, p. 511.
Anthophora longula Rayment, 1947, p. 59. **n. syn.**
Amegilla longula (Rayment) Michener, 1965, p. 217.
Amegilla (Zonamegilla) longula (Rayment) Brooks, 1988, p. 511.
Anthophora subsalteri Rayment, 1947, p. 69. **n. syn.**
Amegilla subsalteri (Rayment) Michener, 1965, p. 217.
Amegilla (Zonamegilla) subsalteri (Rayment) Brooks, 1988, p. 511.

#### Material examined.

229 females and 132males.

#### Type data.

Syntypes of *murrayensis*, male, female, Gunbower, VIC, 16 Mar. 1940, No. G500, “Type” & “allotype”, ANIC 32-034560-1; male, Gunbower, 3 Feb. 1934, 20, ANIC 32-034208.

Syntypes of *longula*, male, female, Orroroo, SA, 3 & 10 Feb. 1940, ANIC 32-034558-9.

#### Decisions for synonymy.

The results of DNA analyses of specimens from across the complete geographical range showed no geographical pattern with respect to sequence variation. The uncorrected sequence divergence was found to be 0–1.3% (Table 1), which is well below the usual limits for conspecific individuals.

The syntypes of *murrayensis* and *longula* were examined and considered to be conspecific. Type material for *subsalteri*, presumed to be the holotype by monotypy, was not found, but from Rayment’s description and drawings (Rayment 1947) together with the collection locality, *subsalteri* is believed to be conspecific with *murrayensis*.

#### Diagnosis.

*Amegilla murrayensis* is a relatively small species with pale yellow face marks and narrow (about 0.3x the width of the disc on T2) apical hair bands which are usually pale blue, occasionally with an orange tint, but never bright orange; female hind tibia with a dark streak at least 0.5× as long as the tibia; T5 with a patch of scattered white hair that narrows laterally and with a longitudinal line of denser white hair that does not intrude significantly into the prepygidial fimbria. Both sexes can be distinguished from other species by hair bands on T1–4 that appear broader laterally below the gradulus because of numerous scattered pale hairs on the disc (Figs 16, 17).

#### Description.

*Female*: Sunnyside, N of Murray Bridge, 35.0500S 139.3600E, 27 Feb 2003, R.Leijs & K. Hogendoorn, SAMA 32-002635.

Length 12 mm; forewing length 8 mm.

***Structure.*** Head: clypeus protuberant, in profile 0.5 width of eye; galea in repose reaching half-way between fore and mid coxae; length of f1 2.8× length of f2, 0.9× length of scape (excluding basal bulb) and 1.6× length of f10; length of f3–9 0.9× width; IOD 1.4× OOD; OS 0.8× OOD. ***Coloration*.** Yellow marks on labrum, mandibles, scape, clypeus, paraocular and supraclypeal areas; inverted T-shape on clypeus. ***Pubescence*.** Head: labrum white, remaining areas predominantly pale yellow with scattered black robust hairs on clypeus, paraocular areas, between antennae, near ocelli and on vertex; gena white. Thorax: scutum ginger intermixed with black hairs; pleura ginger with scattered black hairs under wing base, white ventrally; thoracic sterna white; propodeum laterally light ginger with scattered black hairs. Legs: forefemur posteriorly with long white hairs, outer surface of fore tibia and tarsus white, inner surface of tarsus dark; mid legs dark, except white hairs on apex of the femur, posteriorly on proximal one third of femur and on outer surface of tibia and basitarsus; hind legs black, except white hairs on apex of femur and outer surface of tibia; basitibial streak black, 0.6–0.9 length of femur. Metasoma: apical hair bands on T1–T4 white with iridescence varying from light blue to greenish orange; T5 laterally with long white hairs and few dispersed short hairs (Fig. 32), fimbria black, medial patch around the fimbria, narrowing laterally; S3, S4 dark, posterolateral patches of white hairs; S5 black, laterally with small white patches. ***Punctation*.** Head: clypeus with close, medium sized, deep punctures, 0.3–1.0 puncture widths apart; labrum shiny, with close, small punctures of intermediate depth, 0.2–0.9 puncture widths apart, interspaces almost smooth. Thorax: scutum somewhat shiny, with close punctures, 0.2–0.8 puncture widths apart. Metasoma: T1–T5 with close to open punctures, 0.5–2.0 puncture widths apart.

*Male*: Coen, 13.94415S 143.20022E, QLD, 27 June 2007, SAMA 32-002584, R. Leijs & M. Batley, DNA voucher RB280 (RL783).

Length 11 mm; forewing length 8 mm.

***Structure.*** Head: shortest distance between eyes 0.7 length of eye; clypeus protuberant, in profile 0.5 width of eye; galea in repose reaching halfway between fore and mid coxae; length of f1 1.5× length of f2, 0.5× length of scape (excluding basal bulb) and 0.8× length of f11; length of f3–10 1.2× width; IOD 1.5× OOD; OS 0.7× OOD. Wings: length of marginal cell 0.8× distance from apex of marginal cell to wing tip; length of vein M of hind wing 2.6 times as long as second abscissa of M+Cu; length of jugal lobe about 0.5× length of vannal lobe. Metasoma: apicomedial emargination of S5 wide and deep; S7 windows small, median hair brush 3–4× width, lateral wings of hair brush well developed making an angle of 60° with long axis of brush (Fig. 45); S8 apical emargination deep. ***Pubescence*.** Head: labrum white, remaining areas predominantly pale brown with scattered black robust hairs on clypeus, paraocular areas, between antennae, near ocelli and on vertex; gena white. Thorax: scutum light brown intermixed with black hairs; pleura brown with scattered black hairs under wing base, white ventrally; propodeum laterally light brown with scattered black hairs. Legs: foreleg: femur posteriorly with long white hairs, outer surface of tibia and tarsus white, inner surface of and tarsus dark; mid legs dark, except white hairs on the apex of femur, posteriorly on proximal one third of femur and on outer surface of tibia and basitarsus; hind legs black, except white hairs on apex of femur and outer surface of tibia, small white patch on outer base of basitarsus. Metasoma: apical hair bands on T1–T5 white; T6, T7 black when viewed from behind, light brown when viewed from the side; S2–S5: S3, S4 medially dark, lateral thirds white, S5 medially dark, lateral quarters white. ***Punctation*.** Head: clypeus with punctures 1.0–3.0 puncture widths apart; labrum with medium, shallow punctures 0.5–1.5 puncture widths apart. Thorax: scutum shiny, with close, small, shallow punctures 0.3–1.0 puncture widths apart, interspaces smooth. Metasoma: T1–T5 with shiny, open, fine, shallow punctures, 1.0–3.0 puncture widths apart.

#### Variation.

Males from southern Western Australia often have more black hairs on the clypeus and paraclypeal areas than specimens from northern Queensland or specimens from the Lofty Ranges in South Australia. There is also some variation in the width of the pale patch on T5 in fresh females. Some specimens from the NW Pilbarra and Barrow Island are seemingly larger, have wider tergal bands and a more intense patch of pale hairs on female T5 and have almost ivory face marks and white apical bands on T1–4. These specimens are sufficiently different from *murrayensis* and may belong to an undescribed species. Future collections and molecular work may shed light on their identity.

#### Remarks.

There were some problems with the molecular delineation of *murrayensis* and *pulchra*, as mentioned in the Results and Discussion section. The sequences obtained with the M202/M70 primers resulted in two morphologically similar clades of *murrayensis* specimens, often with specimens from the same localities in different clades (Suppl. material 1: Fig. S1). It also resulted in a number of specimens now believed to be *pulchra* to appear in one of the *murrayensis* clades. Using the CO1 barcoding primers (M414/M423, Hebert et al. 2004) the two *murrayensis* clades collapsed into one (Suppl. material 1: Fig. S2). These problems may have been caused by amplification of a mitochondrial copy in the nuclear genome.

#### Phenology.

**Table:** 

Month:	Jan	Feb	Mar	Apr	May	Jun	Jul	Aug	Sep	Oct	Nov	Dec
**No of records N of 30°S**:	10	6	24	10	24	4	7	25	4	11	17	5
**No of records S of 30°S**:	161	16	6	0	0	0	1	0	0	0	7	16

*Amegilla murrayensis* is the most widespread and common species in the subgenus Zonamegilla. Below 30°S the species is active from November until March, with a peak in January. In the north of the continent they can be found year round possibly with a peak in May.

#### Distribution.

Widespread throughout Australia, but not found in Tasmania (Fig. 11).

### Amegilla (Zonamegilla) paeninsulae sp. n.

Taxon classificationAnimaliaHymenopteraApidae

http://zoobank.org/CC0898E3-E67E-453E-96EF-789D5F9A6361

Figs 10 , 33 , 46

#### Material examined.

26 females and 40 males.

#### Type data.

Holotype: female, N of Bamaga, Qld, 10.84117S 142.42316E, 28 Jun 2007, R. Leijs & M. Batley, DNA voucher RB287 (RL795), SAMA 32-002622.

Allotype: male, N of Bamaga, Qld, 10.84117S 142.42316E, 28 Jun 2007, R. Leijs & M. Batley, DNA voucher RB286 (RL793), SAMA 32-002619.

Paratypes: 2 males, 2 females, same locality data as holotype, ABTC (RL794, RL 796), in absolute ethanol; female, N of Bamaga, Qld, 10.75744S 142.50475E, 28 Jun 2007, R. Leijs & M. Batley, ABTC (RL808), in absolute ethanol; 2 males, N of Bamaga, Qld, 10.74721S 142.58459E, 28 Jun 2007, R. Leijs & M. Batley, ABTC (RL812), in absolute ethanol; 4 females, N of Lockerby, Qld, 10.78141S 142.48837E, 29 Jun 2007, R. Leijs & M. Batley, ABTC (RL818, RL819), in absolute ethanol; 2 males, Iron Range, Qld, 10.74302S 142.23521E, 1 Jul 2007, R. Leijs & M. Batley, DNA voucher RB301 (RL837), SAMA 32-002620.

#### Diagnosis.

*Amegilla paeninsulae* is a distinctive species with orange tergal hair bands, sometimes with green iridescence, orange pubescence on the scutum, orange scopa on the hind legs and no dark basitibial streak in females. Females have T5 covered with scattered orange hair and an orange medial streak (Fig. 33). Males have S7 with a small rounded head and no lightly pigmented windows (Fig. 46).

#### Description.

*Female*: holotype

Length 14 mm; forewing length 9 mm.

***Structure.*** Head: clypeus protuberant, in profile 0.4× width of eye; galea in repose reaching just past forecoxa; length of f1 3× length of f2, equal to length of scape (excluding basal bulb) and 1.4× length of f10; f3–9 as long as wide; IOD 1.5× OOD; OS 0.5× OOD. ***Coloration*.** Yellow marks on labrum, mandibles, scape, clypeus, paraocular and supraclypeal areas, inverted T-shape on clypeus; distal part of flagellum brown ventrally from f2 onwards. ***Pubescence*.** Head: labrum white, clypeus light ginger intermixed with black, hairs in paraocular areas, frons, near ocelli and on vertex, ginger hair darker towards the top; gena white, light ginger towards vertex. Thorax: scutum ginger intermixed with black hair; pleura ginger with scattered black hair under wing base, light ginger ventrally; thoracic sterna light ginger; propodeum laterally ginger with scattered black hairs. Legs: forefemur posteriorly with long, light ginger hair intermixed with some black hairs, outer surface of foretibia and -tarsus light ginger, inner surface of tarsus brown, coxa light ginger; mid legs black, except light ginger hair on apex of femur and on outer surface of tibia and basitarsus and a small patch of light ginger hair on posterior proximal part of femur; hind legs black, except ginger on apex of femur, posterior rim of outer surface of tibia and basal part of basitarsus; basitibial streak absent. Metasoma: apical hair bands on T1–T4 orange-brown with orange and green iridescence; T5 laterally with moderately long light ginger hairs (Fig. 33), fimbria brown, T5 covered with short, light ginger hairs, with light ginger medial streak overlapping the fimbria; S3, S4 very narrow light ginger posterolateral patches; S5 brown. ***Punctation*.** Head: clypeus with close large, deep punctures, 0.2–1.5 puncture widths apart; labrum somewhat shiny, with close, medium, shallow punctures, 0.2–1.0 puncture widths apart. Thorax: scutum shiny, with close to open punctures 0.2–3.0 puncture widths apart. Metasoma: T1–T5 with close to open punctures, 1.0–2.0 puncture widths apart, interspaces transverse pit-reticulate.

*Male*: allotype.

Length 12 mm; forewing length 8 mm.

***Structure.*** Head: shortest distance between eyes 0.7× length of eye; clypeus protuberant, in profile 0.5× width of eye; galea in repose reaching past mid coxa; length of f1 1.6× length of f2, 0.5× length of scape (excluding basal bulb) and 0.9× length of f11; length of f3–10 1.3× width; IOD 1.3× OOD; OS 0.6× OOD. Wings: length of marginal cell 0.9× distance from apex of marginal cell to wing tip; length of vein M of hind wing 2.1× length second abscissa of M+Cu; length of jugal lobe about 0.4× length of vannal lobe. Metasoma: apicomedial emargination of S5 wide and deep; S7 windows absent; S7 median hair brush, broad 2× width; S7 with a small rounded head, very wide medial ridge leaving no lightly pigmented windows, no apical projection and a flattened apical margin (Fig. 46); S8 apical emargination deep. ***Pubescence*.** Head: labrum white, clypeus and paraocular areas black, remaining areas predominantly ginger with scattered black hairs on frons, near ocelli and on vertex; gena white, light ginger towards vertex. Thorax: scutum orange brown intermixed with black hair; pleura orange brown, intermixed with black hair, whitish ventrally; thoracic sterna greyish white; propodeum laterally orange-brown with scattered black hairs. Legs: forefemur posteriorly with light ginger hair, outer surface of foretibia and -tarsus orange-brown, inner surface of tarsus brown, coxa greyish white; mid legs dark, except orange-brown on apex of femur, posteriorly proximally on femur and on outer surface of tibia and tarsus; hind legs black, except orange-brown hair on apex of femur and outer surface of tibia and tarsus, light ginger tuft on apex of tibia. Metasoma: apical hair bands on T1–T5 orange-brown with orange and green iridescence; T6 with black medial patch, remaining hair orange when viewed from behind, light brown when viewed from the side, T7 black when viewed from behind with a few orange hairs laterally, brown when viewed from the side; S2–S5 medially light orange, laterally with light orange patches; S6 light orange. ***Punctation*.** Head: clypeus punctures 1.0–4.0 puncture widths apart; labrum with medium, shallow punctures 0.8–3.0 puncture widths apart. Thorax: scutum shiny, with close to sparse, medium, shallow punctures 0.2–4 puncture widths apart. Metasoma: T1–T5 somewhat shiny, with close to open, fine, shallow punctures, 1.0–2.0 puncture widths apart; interspaces pit-reticulate.

#### Variation.

Most males have predominantly orange tergal bands, while those of females are usually a mixture of iridescent green and orange. The hair on the sterna and coxae of most males is pale orange and some males lack the medial brown patch on T6.

#### Phenology.

**Table:** 

Month:	Jan	Feb	Mar	Apr	May	Jun	Jul	Aug	Sep	Oct	Nov	Dec
**No. of records**:	0	0	0	7	6	26	18	1	0	0	0	4

#### Distribution.

In tropical rainforest patches on Cape York, Queensland (Fig. 10).

#### Etymology.

The specific epithet refers to its distribution on Cape York Peninsula.

### Amegilla (Zonamegilla) pulchra

Taxon classificationAnimaliaHymenopteraApidae

(Smith)

Figs 12 , 34 , 47

Anthophora pulchra Smith, 1854, p. 335.
Amegilla pulchra (Smith) Michener, 1965, p. 217.
Amegilla (Zonamegilla) pulchra (Smith) Brooks, 1988, p. 511.
Anthophora holmesi Rayment, 1947, p. 56, **n. syn.**
Amegilla holmesi (Rayment) Michener, 1965, p. 216.
Amegilla (Zonamegilla) holmesi (Rayment) Brooks, 1988, p. 511.
Anthophora parapulchra Rayment, 1947, p.61, **n. syn.**
Amegilla parapulchra (Rayment) Michener, 1965, p. 217.
Amegilla (Zonamegilla) parapulchra (Rayment) Brooks, 1988, p. 511.
Anthophora salteri Cockerell, 1905, p. 398, **n. syn.**
Amegilla salteri (Cockerell) Michener, 1965, p. 217.
Amegilla (Zonamegilla) salteri (Cockerell) Brooks, 1988, p. 511.
Anthophora pulchra townleyella Rayment, 1947, p. 67. **n. syn.**
Amegilla townleyella (Rayment) Michener, 1965, p. 217.
Anthophora shafferyella Rayment, 1947, p. 70. **n. syn.**
Amegilla shafferyella (Rayment) Michener, 1965, p. 217.
Amegilla (Zonamegilla) shafferyella (Rayment) Brooks, 1988, p. 511.
Anthophora perpulchra wallaciella Rayment, 1947, p. 65. **n. syn.**
Amegilla wallaciella (Rayment) Michener, 1965, p. 217.

#### Material examined.

171 females and 182 males.

#### Type data.

Lectotype of *pulchra*, male, “pulchra type Sm.”, “Anthophora pulchra type” BMNH 17B.666b, here designated.

Another, female, specimen in the British Museum bore the following labels: Moreton Bay; pulchra type ♀ Sm; Anthophora pulchra type; Amegilla niveocincta SM D.B. Baker 2008: BMNH 17B.666a. As the specimen was unlike any Australian species, we have no reason to doubt Baker’s identification and the corollary that there has been a labelling error.

Syntype of *townleyella*, female, Lismore, NSW, 8 Feb 1940, “Type, Anthophora salteri townleyella”, ANIC 32-034170.

Lectotype of *parapulchra*, female, Hunters Hill, Sydney, Dec. 1939, “No 28”, “Type, Anthophora parapulchra”, ANIC 32-034562, here designated.

Syntypes of *holmesi*: female, Como, NSW, 4 Apr. 1940, “Type, Anthophora holmesi”, ANIC 32-034573; male, Sydney, NSW, 20 Mar. 1940, “Allotype, Anthophora perpulchra holmesi” , ANIC 32-034574; female, Woollahra, NSW, 26 Mar. 1940, ANIC 32-033648; male, Hunters Hill HS, NSW, 20 Mar. 1940, ANIC 32-033651.

Holotype of *salteri* (by monotypy): male, N.S.Wales, BMNH 17B.665.

Holotype of *shaffereyella* (by monotypy): male, Mossman, Queensland, Feb. 1940, Anthophora salteri shafferyella, ANIC 32-033534.

Lectotype of *perpulchrawallaciella*: female, Hunters Hill, NSW, 20 Mar. 1940, “type, Anthophora perpulchra wallaciella”, ANIC 32-034571, here designated.

#### Decisions for synonymy.

Examination of the above types indicated that *holmesi*, *parapulchra*, *salteri* and *townleyella* were conspecific with *pulchra*. The holotype of *shafferyella* had a dense hair patch on S5 an orange tint in the tergal hair bands like *adelaidae*, as suggested by Rayment (1947) but the shape of S7 was unmistakably that of *pulchra*, not *adelaidae*. When examined carefully, the type of *salteri* was found to be indistinguishable from *pulchra*. In particular, it was found that the emargination of S5 was normal, though the hair pattern made it appear superficially as reported by Cockerell (1905).

#### Diagnosis.

For diagnosis and description we used specimens from the Sydney area because they vary less compared to those from the Brisbane area (see also under variation and remarks)

*Amegilla pulchra* is a species with ivory face marks and paraocular areas with some long dark hairs. Tergites with pale blue or white hair bands that are not broadened laterally below the lateral arm of the gradulus (Figs 18, 19). Female T5 with a broad oval patch of white hair, usually not extended laterally, and a medial line of denser white hair that does not extend greatly into the prepygidial fimbria; female hind tibia with dark streak at least 0.5× times as long as the tibia. Male S6 with tuft dark hair apicomedially; S7 with narrow rounded head and small windows.

#### Description.

*Female*: East Kurrajong, 33.500S 150.767E, NSW, 8 Jan 2003, R Spooner Hart, DNA voucher RB083 (RL494), SAMA 32-002612.

Length 14 mm; forewing length 9 mm.

***Structure.*** Head: clypeus protuberant, in profile 0.4× width of eye; galea in repose reaching half-way between coxa of fore and mid legs; length of f1 2.8× length of f2, 0.8× length of scape (excluding basal bulb) and 1.6× length of f10; length of f3–9 0.9× width; IOD 1.2× OOD; OS 0.6× OOD. ***Coloration*.** Ivory marks on labrum, mandibles, scape, clypeus, paraocular and supraclypeal areas; inverted T-shape on clypeus. ***Pubescence*.** Head: labrum white, remaining areas predominantly pale, darker towards vertex with scattered black robust hairs on clypeus, paraocular areas, between antennae, near ocelli and on vertex; gena white, ginger towards vertex. Thorax: scutum ginger intermixed with many black hairs, therefore overall darker than other species; pleura ginger with scattered black hair under wing base, white ventrally; thoracic sterna white; propodeum laterally ginger with scattered black hair. Legs: forefemur posteriorly with long white hair, outer surface of foretibia and -tarsus greyish white, inner surface of tarsus dark; mid legs black, except white hair on apex of femur and on outer surface of tibia and basitarsus, contiguous short white hairs on posterior proximal part of femur; hind legs black except white hair on apex of femur and outer surface of tibia, very small white patch on basal part of basitarsus; basitibial streak black, 0.8× length of femur. Metasoma: apical hair bands on T1–T4 white with very weak light blue iridescence; T5 laterally with moderately long white hair (Fig. 34), fimbria dark, medial patch ovoid with weak medial stripe; S3, S4 dark, posterolateral patches of white hairs; S5 dark, laterally with small white patches. ***Punctation*.** Head: clypeus with close, medium, deep punctures, 0.1–0.8 puncture widths apart; labrum somewhat shiny, with close to open, small punctures of intermediate depth, 0.5–2.0 puncture widths apart. Thorax: scutum somewhat shiny, with close punctures, 0.2–1.0 puncture widths apart. Metasoma: T1–T5 with open punctures, 0.8–1.5 puncture widths apart.

*Male*: Northbridge, 33.800S 151.217E, NSW, 27 Feb 2003, M. Bell, DNA voucher RB078 (RL487), SAMA 32-002611.

Length 12 mm; forewing length 8 mm.

***Structure.*** Head: shortest distance between eyes 0.5× length of eye; clypeus protuberant, in profile 0.5× width of eye; galea in repose reaching just past forecoxa; length of f1 2× length of f2, 0.6× length of scape (excluding basal bulb) and 1.1× length of f11; length of f3–10 1.2× width; IOD 1.4× OOD; OS 0.7× OOD. Wings: length of marginal cell 0.8× distance from apex of marginal cell to wing tip; length of vein M of hind wing 2.8× length second abscissa of M+Cu; length of jugal lobe about 0.4× length of vannal lobe. Metasoma: apicomedial emargination of S5 narrow and deep; S7 windows medium size, median hair brush 3× width, lateral wings of hair brush narrow but well developed with 110° angle between them (Fig. 47). ***Pubescence*.** Head: labrum white, clypeus and paraocular marks predominantly black, remaining pubescence grey, scattered black hairs between antennae, near ocelli and on vertex; gena white. Thorax: scutum pale brown intermixed with black hair; pleura pale brown with scattered black hair under wing base, white ventrally; propodeum laterally pale brown with scattered black hair. Legs: forefemur posteriorly with long white hair, outer surface of tibia and tarsus greyish, inner surface of tarsus dark; mid legs dark, except white hair on the apex of femur, a small patch near the apex of the femur and on outer surface of tibia and basitarsus; hind legs dark, except white hair on apex of femur and outer surface of tibia, small white patch on outer base of basitarsus. Metasoma: apical hair bands on T1–T5 white, lacking iridescence; parts that are not covered by hair bands dark brown; T6, T7 black when viewed from behind, brown when viewed from side; S2–S5 medial 50% dark, laterally white. ***Punctation*.** Head: clypeus with punctures 1.0–2.0 puncture widths apart; labrum with medium, shallow punctures 0.7–1.5 puncture widths apart. Thorax: scutum somewhat shiny, with close, medium, shallow punctures 0.4–0.9 puncture widths apart. Metasoma: T1–T5 somewhat shiny, with open, fine, shallow punctures, 1.0–2.0 puncture widths apart.

#### Variation.

Most specimens of *pulchra* in collections have relatively narrow white bands with small amounts of green-blue iridescence and ivory face markings. However, examination of a series of fresh specimens collected from the Brisbane area on two consecutive days showed iridescent bands that varied in colour from green-blue to orange or white. Some specimens also had yellowish face marks and specimens varied with respect to the shape of the white patch on female T5, some approaching those found in *murrayensis*. There was however no correlation with sequenced mitochondrial DNA, because the majority of those specimens shared the same mitochondrial haplotypes (Suppl. material 1: Fig. S2).

#### Remarks.

In all phylogenetic analyses *murrayensis* and *pulchra* appeared as sister species (Fig. 2, Suppl. material 1: S1, S2). As mentioned in the Results and Discussion section and under the remarks for *murrayensis* there are some unresolved problems with the genetics of the two species, probably due to the presence of a mitochondrial copy in the nuclear genome, which makes molecular identification of these two species not straightforward. DNA barcoding of *pulchra* specimens using the standard barcoding primers (Hebert et al. 2004) resulted in two distinct clades, separating morphologically similar specimens with identical collection details (Suppl. material 1: Fig. S2). Future results of DNA barcoding of *pulchra* specimens should therefore be interpreted with caution. Additionally, morphological variation of the above mentioned specimens collected in the Brisbane area and museum specimens from that area and others incidentally showed *murrayensis* characters such as pale yellow face markings and hair patch that reaches the lateral margins of the female T5. This may be a consequence of intraspecific variability, but it is also possible that these character states result from hybridization with *murrayensis*. Hybridization regularly occurs between closely related species that historically had allopatric distributions, but that in more recent time became in secondary contact (Kawakami and Butlin 2012) as could be the case with *pulchra* and *murrayensis* when their distribution patterns are considered (Fig. 2I, J). Unfortunately, testing above hypotheses about nuclear paralogues and introgression is beyond the scope of this paper.

#### Phenology.

**Table:** 

Month:	Jan	Feb	Mar	Apr	May	Jun	Jul	Aug	Sep	Oct	Nov	Dec
**No. of records**:	38	67	127	51	5	3	0	2	7	12	44	51

#### Distribution.

Mainly east of the Great Dividing Range in New South Wales and Queensland (Fig. 12). This species has also been found on Fiji. Specimens from the Pacific islands had mitochondrial haplotypes identical to those from the Brisbane area and were probably introduced there (Groom et al. 2014).

### Amegilla (Zonamegilla) thorogoodi

Taxon classificationAnimaliaHymenopteraApidae

(Rayment)

Figs 13 , 35 , 48

Anthophora thorogoodi Rayment, 1939, p. 289.
Amegilla thorogoodi (Rayment) Michener, 1965, p. 217.
Amegilla (Zonamegilla) thorogoodi (Rayment) Brooks, 1988, p. 511.

#### Material examined.

95 females and 68 males.

#### Type data.

Holotype of *thorogoodi*: male, Proserpine, QLD, 15 Nov. 1937, ANIC 32-033973. (Hidden sterna and genitalia missing.)

#### Diagnosis.

*Amegilla thorogoodi* is distinguished from other Australian *Zonamegilla* species by the following characters: Scutal hair of both sexes brown; apical tergal hair bands predominantly blue. Hind basitibial streak of females short; pale pubescence of T5 forming a large medial patch (Fig. 35). Apical margin of S5 of males lacking a medial patch of dense hair; S7 with a moderately narrow medial ridge, a rounded apical projection and a Y-shaped brush (Fig. 48).

#### Description.

*Female*: Iron Range, 12.743S 143.2352E, 1 Jul 2007, R. Leijs & M. Batley, DNA voucher RB302 (RL838), SAMA 32-002603.

Length 13.5 mm; forewing length 9 mm.

***Structure.*** Head: shortest distance between eyes equal to the length of the eye; clypeus protuberant, in profile 0.4× width of eye; galea in repose reaching just reaching mid coxa; length of f1 3.2× length of f2, 0.8× length of scape (excluding basal bulb) and 1.5× length of f10; length of f3–9 0.8× width; IOD 1.6× OOD; OS 0.5× OOD. ***Coloration*.** Black except labrum, mandibles, scape, clypeus, paraocular and supraclypeal areas with pale yellow marks; pale mark on clypeus inverted T-shaped. ***Pubescence*.** Head: labrum white, remaining areas pale yellow, darker towards vertex with scattered black robust hairs on clypeus, paraocular areas, between antennae, near ocelli and on vertex; gena white, ginger towards vertex. Thorax: scutum ginger intermixed with black hair; pleura ginger with scattered black hair under wing base, white ventrally; thoracic sterna white; propodeum laterally light ginger with scattered black hairs. Legs: forefemur posteriorly with long white hair, foretibia and -tarsus whitish on outer surface, dark on inner surface; mid legs black, except greyish white hair on apex of femur and on outer surface of tibia and basitarsus and a dense patch of short white hair on posterior proximal part of femur; hind legs black, except white hair on apex of femur; tibia white on posterior rim, greyish-white with orange tinge in scopal area and black anteriorly; whitish patch on basal part of basitarsus; basitibial streak black, 0.3x length of femur. Metasoma: apical hair bands on margin T1–T4 iridescent blue with orange tinge, especially across anterior edge; T5 laterally with moderately long white hairs (Fig. 35), fimbria dark, medial patch distinct, complete, narrowing laterally, iridescence weak light blue, medial stripe overlapping fimbria; S3, S4 dark, posterolateral patches of white hairs; S5 dark. ***Punctation***. Head: clypeus with close, medium sized, deep punctures, 0.1–0.8 puncture widths apart; labrum somewhat shiny, with close, small, shallow punctures, 0.3–1.0 puncture widths apart. Thorax: scutum shiny, with punctures 0.1–1.5 puncture widths apart. Metasoma: T1–T5 with open, punctures, 0.5–1.5 puncture widths apart.

*Male*: Bloomfield near rubbish tip, 15.9011S 143.34161E, Qld, 2 Jul 2007, DNA voucher RB311 (RL865), SAMA 32-002599.

Length 13 mm; forewing length 8 mm.

***Structure.*** Head: shortest distance between eyes 0.7× length of eye; clypeus protuberant, in profile 0.4× width of eye; galea in repose reaching halfway between fore and mid coxae; length of f1 2.1× length of f2, 0.5× length of scape (excluding basal bulb) and as long f11; length of f3–10 1.3× width; IOD 1.6× OOD; OS 0.7× OOD. Wings: length of marginal cell 0.9× distance from apex of marginal cell to wing tip; length of vein M of hind wing 1.8× length of second abscissa of M+Cu; length of jugal lobe about 0.4× length of vannal lobe. Metasoma: apicomedial emargination of S5 wide and very shallow; S7 head large, medial ridge moderately narrow resulting in a narrow apical projection, large weakly pigmented windows and an inverted Y-shaped brush (Fig. 48); S8 apical emargination deep. ***Pubescence*.** Head: labrum white, remaining areas predominantly pale yellow, darker towards the vertex; scattered black robust hairs on clypeus, paraocular areas, frons, near ocelli and on vertex; gena white. Thorax: scutum ginger intermixed with black hairs; pleura ginger, white ventrally; propodeum laterally light ginger with scattered black hairs. Legs: forefemur posteriorly with long white hair, outer surface of tibia, tarsus light ginger, inner surface of tarsus dark; mid legs black, except white hair on apex of femur, posteriorly proximally on femur and on outer surface of tibia and basitarsus; hind legs black, except white hair on apex of femur and outer surface of tibia, white patch on base of basitarsus, orange tuft near inner tibial spur. Metasoma: apical hair bands on T1–T5 white with orange tinge and blue-green iridescence; T6 white on apical margin, remaining hair black when viewed from behind, brown when viewed from side; S2–S5 medially dark, laterally with white patches; s6 dark and few robust white hairs laterally. ***Punctation*.** Head: clypeus punctures 1.5–3.0 puncture widths apart; labrum: with small, shallow punctures 0.1–1.5 puncture widths apart. Thorax: scutum shiny, with close small, shallow punctures, 0.2–1.5 puncture widths apart. Abdomen: T1–T5 somewhat shiny, with open, shallow punctures, 0.8–2.0 puncture widths apart.

#### Variation.

Variation in the colour of the tergal bands caused by fading of the colour and varying amounts of orange can complicate identification, but no other significant variation was observed. Only two specimens had scutal hair with a grey appearance.

#### Phenology.

**Table:** 

Month:	Jan	Feb	Mar	Apr	May	Jun	Jul	Aug	Sep	Oct	Nov	Dec
**No. of records**:	6	2	27	23	20	24	12	5	4	3	2	13

#### Distribution.

In the subtropics and tropics along the east coast of Queensland (Fig. 13), also found in southern Papua New Guinea.

#### Remarks.

Closely related to the allopatric species *walkeri*, but easily distinguished by the brown scutal hair in both sexes. Similar to *Amegilla asserta* and *Amegilla indistincta*, but females distinguished by the length of the hind basitibial streak. Males may be distinguished from those of *asserta* by the absence of a hair patch on S5 and from *indistincta* by the colour of the tergal hair bands and the shape of S7 (Fig. 48).

### Amegilla (Zonamegilla) viridicingulata sp. n.

Taxon classificationAnimaliaHymenopteraApidae

http://zoobank.org/2FC90985-6CF3-4B14-816A-B2DD74C90B7F

Figs 14 , 36 , 49

#### Material examined.

16 females and 27males.

#### Type data.

Holotype: Female, Cooktown, Qld, 15.4898S 145.2413E, 3 Jul 2007, R. Leijs & M. Batley, DNA voucher RB308 (RL857), SAMA 32-002624.

Allotype: Cooktown, Qld, 15.4898S 145.2413E, 3 Jul 2007, R. Leijs & M. Batley, DNA voucher RB309 (RL859), SAMA 32-002625.

Paratypes: 4 males, 3 females, same locality data as holotype, SAMA 32-002626, DNA voucher RB310 (RL860), ABTC (RL855, RL856, RL858) in absolute alcohol; male, Cooktown, Qld, 15.4667S 145.2500E, 17 Jul 1982, N.W. Rodd, AM K-316266; female, Cooktown, Qld, 15.4667S 145.2500E, 15 Jul 1982, N.W. Rodd, AM K-316267.

#### Diagnosis.

*Amegilla viridicingulata* is a distinctive species with orange-brown scutal pubescence, tergal hair bands with green iridescence and orange hair on the hind legs in both sexes. Females have a hind tibial scopa without a dark streak and T5 with a broad area of scattered white hair above the fimbria (Fig. 36). Males have pale hair across the sterna, particularly S3, 4 and a distinctive S7 (Fig. 49).

#### Description.

*Female*: holotype.

Length 12 mm; forewing length 8.5 mm.

***Structure.*** Head: clypeus protuberant, in profile 0.44× width of eye; galea in repose reaching reaching mid coxa; length of f1 4× length of f2, 0.9× length of scape (excluding basal bulb) and 1.6× length of f10; f3–9 as long as wide; IOD 1.3× OOD; OS 0.5× OOD. ***Coloration*.** Pale yellow marks on labrum, mandibles, scape, clypeus, paraocular and supraclypeal areas, inverted T-shape on clypeus; f2 red-brown ventrally, remainder of flagellum brown ventrally. ***Pubescence*.** Head: labrum white, light ginger intermixed with black hair on clypeus, paraocular areas, frons, near ocelli and on vertex, gena white, light ginger towards vertex. Thorax: scutum ginger intermixed with black hair; pleura ginger with few black hairs under wing base, white ventrally; thoracic sterna light ginger; propodeum laterally ginger with few black hairs. Legs: forefemur posteriorly with long white hair, outer surface of tibia and tarsus light ginger, inner surface of tarsus brown; mid legs black, except light ginger hair on apex of femur and on outer surface of tibia and basitarsus and a narrow line of light ginger hairs on posterior proximal part of femur; hind legs black, except ginger hair on apex of femur, posterior rim of the outer surface of tibia and basal part of basitarsus, a white tuft on the apex of tibia; basitibial streak absent. Metasoma: apical hair bands on T1–T4 light orange-brown with clear green iridescence; T5 laterally white, fimbria brown, medial patch well developed, centrally white, laterally greyish-white and narrowing, medial streak overlapping fimbria (Fig. 36); S3, S4 dark with posterolateral patches of white hair; S5 dark. ***Punctation*.** Head: clypeus with close, medium, deep punctures, 0.1–1.0 puncture widths apart; labrum somewhat shiny, with close, small, shallow punctures, 0.3–1.0 puncture widths apart. Thorax: scutum shiny, with close punctures 0.1–0.7 puncture widths apart. Metasoma: T1–T5 with close to open punctures, 0.5–2.0 puncture widths apart.

*Male*: allotype.

Length 11 mm; forewing length 8 mm.

***Structure.*** Head: shortest distance between eyes 0.7× length of eye; clypeus protuberant, in profile 0.5× width of eye; galea in repose reaching halfway between fore and mid coxae; length of f1 1.6× length of f2, 0.5× length of scape (excluding basal bulb) and 0.8× length of f11; length of f3–10 1.3× width; IOD 1.3× OOD; OS 0.5× OOD. Wings: length of marginal cell equal to distance from apex of marginal cell to wing tip; length of vein M of hind wing 1.6× length second abscissa of M+Cu; length of jugal lobe about 0.3× length of vannal lobe. Metasoma: apicomedial emargination of S5 wide and shallow; S7 with very wide medial ridge, resulting in a flattened apex with no apical projection, very small weakly pigmented areas and an inverted T-shaped brush, weak and broadened towards the apex (Fig. 49); S8 apical emargination deep. ***Pubescence*.** Head: labrum white; clypeus and paraocular areas predominantly black, remaining areas with scattered black hair on frons, near ocelli and on vertex; gena white, ginger towards vertex. Thorax: scutum ginger intermixed with black hair; pleura light brown with green iridescence; propodeum laterally ginger. Legs: forefemur posteriorly with long white greyish hair, outer surface of tibia and tarsus pale brown, inner surface of tarsus brown; mid legs dark, except pale brown hair on the apex of femur, posteriorly proximally on femur and on outer surface of tibia and basitarsus; hind legs black, except light ginger hair on apex of femur and outer surface of tibia, light ginger patch on base of basitarsus, white tuft on apex of tibia. Metasoma: apical hair bands on T1–T5 light orange-brown with green iridescence; T6 with a row of pale ginger hair on apical margin, remaining hair black when viewed from behind, brown when viewed from the side; T7 black; S2–S5 greyish white. ***Punctation*.** Head: clypeus punctures 0.5–2.0 puncture widths apart; labrum: medium, shallow punctures 0.8–2.5 puncture widths apart. Thorax: scutum shiny, close to open, medium, shallow punctures, 0.2–2.5 puncture widths apart. Metasoma: T1–T5 somewhat shiny, with close to open, fine, shallow punctures, 1.0–2.0 puncture widths apart.

#### Variation.

Some males had white metasomal hair bands with blue reflections, making them difficult to recognise before dissection.

#### Phenology.

**Table:** 

Month:	Jan	Feb	Mar	Apr	May	Jun	Jul	Aug	Sep	Oct	Nov	Dec
**No. of records**:	3	6	2	7	1	5	8	3	3	1	3	3

#### Distribution.

Coastal NE Queensland (Fig. 14).

#### Etymology.

The specific epithet is a Latin adjective meaning green banded.

### Amegilla (Zonamegilla) walkeri

Taxon classificationAnimaliaHymenopteraApidae

(Cockerell)

Figs 13 , 37 , 50

Anthophora walkeri Cockerell, 1905, p. 396.
Amegilla walkeri (Cockerell) Michener, 1965, p. 217.
Amegilla (Zonamegilla) walkeri (Cockerell) Brooks, 1988, p. 511.
Anthophora darwini Cockerell, 1910, p. 409. **n. syn.**
Amegilla darwini (Cockerell) Michener, 1965, p. 216.

#### Type data.

Holotype of *walkeri*, female, Baudin I. Long Reef, WA, 91-155, 4593, BMNH 17B.663.

Holotype of *darwini*, male, P. Darwin, Turner Coll. 1910-7, 11-02, “Anthophora darwini, Ckll”, BMNH 17B.448.

#### Decision for synonymy.

The holotype of *darwini* bears a label “Amegilla=walkeri, M.A. Lieftinck, 1958”. Examination the type specimens confirmed Lieftinck’s and Brook’s (1988) decisions to synonymise *darwini* with *walkeri*.

#### Material examined.

66 females and 63 males.

#### Diagnosis.

Both sexes of *walkeri* have grey pubescence on the scutum due of a mixture of black and white hair and conspicuous light blue, metallic hair bands on the terga. Females have a broad band of white hair bordering the fimbria on T5 (Fig. 37).

#### Description.

*Female*: Darwin, East Point, 28 Feb.2006, 12.4130S 130.8300E, D. A. Young, SAMA 32-002593, DNA voucher RB127 (RL714).

Length 12 mm; forewing length 8.5 mm.

***Structure.*** Head: clypeus protuberant, in profile 0.4× width of eye; galea in repose reaching just past fore coxa; length of f1 2.6× length of f2, 0.7× length of scape (excluding basal bulb) and 1.6× length of f10; length of f3–9 1.1× width; IOD 1.3× OOD; OS 0.5× OOD. ***Coloration*.** Yellow marks on labrum, mandibles, scape, clypeus, paraocular and supraclypeal areas; inverted T-shape on clypeus; f2 orange, f3–10 brown ventrally. ***Pubescence*.** Head: white, intermixed with black hair on clypeus, paraocular areas, frons, near ocelli and on vertex; gena white. Thorax: scutum white intermixed with black hair, producing an overall grey appearance; pleura white with scattered black hair under wing base; thoracic sterna white; propodeum laterally white with scattered black hair. Legs: fore femur posteriorly with long white hair, outer surface of tibia and tarsus white, inner surface of tarsus dark; mid legs black, except white hair on the apex of femur and on outer surface of tibia and basitarsus and a dense streak of short white hair on posterior proximal part of femur; hind legs black, except white hair on apex of femur, posterior rim of the outer surface of tibia, white patch on basal part of basitarsus; basitibial streak black, 0.4× length of femur. Metasoma: apical hair bands on T1–T4 white with clear light blue iridescence; T5 laterally with moderately long white hair (Fig. 37), fimbria dark, a broad patch of scattered white hair above fimbria with a denser medial stripe overlapping fimbria; S3, S4 dark, posterolateral patches of white hair; S5 dark. ***Punctation*.** Head: clypeus with close, medium sized, deep punctures, 0.1–1.0 puncture widths apart; labrum somewhat shiny, with close, medium punctures of intermediate depth, 0.1–0.8 puncture widths apart. Thorax: scutum somewhat shiny, with close, punctures, 0.2–1.2 puncture widths apart. Metasoma: T1–T5 with open punctures, 0.8–1.8 puncture widths apart.

*Male*: Darwin, East Point, 28 Feb.2006, 12.4130S 130.8300E, D. A. Young, SAMA 32-002596, DNA voucher RB126 (RL713).

Length 10 mm; forewing length 7 mm.

***Structure.*** Head: shortest distance between eyes 0.7× length of eye; clypeus protuberant, in profile 0.5× width of eye; galea in repose reaching just past mid coxa; length of f1 1.8× length of f2, 0.9× length of scape (excluding basal bulb) and 0.9× length of f11; length of f3–10 1.2× width; IOD 1.3× OOD; OS 0.6× OOD. Wings: length of marginal cell 0.9× distance from apex of marginal cell to wing tip; length of vein M of hind wing 1.6× length of second abscissa of M+Cu; length of jugal lobe about 0.4× length of vannal lobe. Metasoma: apicomedial emargination of S5 wide and shallow; S7 head large, medial ridge moderately narrow resulting in a narrow, rounded apical projection, large weakly pigmented windows and an inverted Y-shaped brush (Fig. 50); S8 apical emargination of intermediate depth. ***Pubescence*.** Head: labrum white, black hair on clypeus, white and scattered black hair on paraocular areas, on frons, near ocelli and on vertex; gena white. Thorax: scutum white intermixed with black hair, producing an overall grey appearance; pleura white with scattered black hair under wing base; propodeum laterally white with scattered black hair. Legs: forefemur posteriorly with long white hair, outer surface of tibia and tarsus white, inner surface of tarsus dark; mid legs dark, except white hair on the apex of femur, posteriorly proximally on femur and on outer surface of tibia and basitarsus; hind legs dark, except white hair on apex of femur and outer surface of tibia, a few white hairs on base of basitarsus. Metasoma: apical hair bands on T1–T5 white with clear light blue iridescence; T6, T7 black when viewed from behind, brown when viewed laterally; S2–S5 medially dark, laterally with white patches. ***Punctation*.** Head: clypeus medium, shallow punctures 1.0–2.0 puncture widths apart; labrum: small, shallow punctures 0.7–2.0 puncture widths apart. Thorax: scutum shiny, with open, small, shallow punctures 0.5–2.5 puncture widths apart. Metasoma: T1–T5 somewhat shiny, with open, fine, shallow punctures, 1.0–2.0 puncture widths apart.

#### Phenology.

**Table:** 

Month:	Jan	Feb	Mar	Apr	May	Jun	Jul	Aug	Sep	Oct	Nov	Dec
**No. of records**:	6	17	15	3	26	11	3	2	15	12	15	8

#### Distribution.

In tropical areas of the Northern Territories and Western Australia (Kimberleys) (Fig. 13).

#### Remarks.

Closely related to the allopatric species *thorogoodi* from which it can be distinguished by the colour of the thoracic hair.

## Author contributions

R.L., M.B. and K.H. designed the research, collected and examined specimens and wrote the paper, R.L. extracted and sequenced DNA and interpreted the molecular data.

## Supplementary Material

XML Treatment for Asaropoda

XML Treatment for Notomegilla

XML Treatment for Amegilla (Notomegilla) aeruginosa

XML Treatment for Amegilla (Notomegilla) chlorocyanea

XML Treatment for Zonamegilla

XML Treatment for Amegilla (Zonamegilla) adelaidae

XML Treatment for Amegilla (Zonamegilla) alpha

XML Treatment for Amegilla (Zonamegilla) asserta

XML Treatment for Amegilla (Zonamegilla) cingulata

XML Treatment for Amegilla (Zonamegilla) indistincta

XML Treatment for Amegilla (Zonamegilla) karlba

XML Treatment for Amegilla (Zonamegilla) murrayensis

XML Treatment for Amegilla (Zonamegilla) paeninsulae

XML Treatment for Amegilla (Zonamegilla) pulchra

XML Treatment for Amegilla (Zonamegilla) thorogoodi

XML Treatment for Amegilla (Zonamegilla) viridicingulata

XML Treatment for Amegilla (Zonamegilla) walkeri

## Supplementary materials

Supplementary material 1 Phylogenetic trees and table of DNA voucher numbers, including Genbank accession numbers Data type: phylogenetic data Explanation note: **Figure S1.** CO1 (primers M70/M202) neighbour-joining tree calculated using uncorrected sequence divergence in PAUP*. Terminals are labelled with RB-numbers, which refer to Table S1, sequencing primer or ‘cons’, indicating consensus sequence based on forward and reverse sequencing.
**Figure S2.** CO1 (primers M414/M423) neighbour-joining tree calculated using uncorrected sequence divergence in PAUP*. Terminals are labelled with RB-numbers, which refer to Table S1, sequencing primer or ‘cons’, indicating consensus sequence based on forward and reverse sequencing.
**Table S1.** Table of DNA specimens and GenBank accession numbers and locality data. This dataset is made available under the Open Database License (http://opendatacommons.org/licenses/odbl/1.0/). The Open Database License (ODbL) is a license agreement intended to allow users to freely share, modify, and use this Dataset while maintaining this same freedom for others, provided that the original source and author(s) are credited.Remko Leijs, Michael Batley, Katja Hogendoorn

Supplementary material 2 Examined specimens Data type: specimens database Explanation note: The database contains information copied from specimen labels for each species: including repository, catalogue numbers and DNA voucher numbers. This dataset is made available under the Open Database License (http://opendatacommons.org/licenses/odbl/1.0/). The Open Database License (ODbL) is a license agreement intended to allow users to freely share, modify, and use this Dataset while maintaining this same freedom for others, provided that the original source and author(s) are credited.Remko Leijs, Michael Batley, Katja Hogendoorn

## References

[B1] Anderson GJ Symon DE (1988) Insect foragers on *Solanum* flowers in Australia. Annals of the Missouri Botanical Garden 75: 842–852. https://doi.org/10.2307/2399372

[B2] Baker DB (1996) The identity of *Apis zonata* Linneaus, 1758 (Insecta: Hymenoptera: Apoidea: Anthophoridae). Reichenbachia 31: 203–206.

[B3] Bell MC Spooner-Hart RN Haigh AM (2006) Pollination of Greenhouse Tomatoes by the Australian blue banded Bee Amegilla (Zonamegilla) holmesi (Hymenoptera: Apidae). Journal of Economic Entomology 99: 437–442. https://doi.org/10.1093/jee/99.2.437 10.1603/0022-0493-99.2.437 16686144

[B4] Bensasson D Zhang D-X Hartl DL Hewitt GM (2001) Mitochondrial pseudogenes: evolution’s misplaced witnesses. Trends in Ecology and Evolution 16: 314–321. https://doi.org/10.1016/S0169-5347(01)02151-6 10.1016/s0169-5347(01)02151-6 11369110

[B5] Breed B Ford F (2007) Native mice and rats. CSIRO Publishing, Collingwood.

[B6] Brooks RW (1988) Systematics and phylogeny of the Anthophorine bees (Hymenoptera: Anthophoridae; Anthophorini). University of Kansas Science Bullletin 53: 436–575.

[B7] Brooks RW (1993) A new *Amegilla* (Hymenoptera: Anthophoridae) from Western Australia. Records of the Western Australian Museum 16: 279–282.

[B8] Brower AVZ (1994) Rapid morphological radiation and convergence among races of the butterfly *Heliconius erato* inferred from patterns of mitochondrial DNA evolution. Proceedings of the National Academy of Sciences, USA 91: 6491–6495. https://doi.org/10.1073/pnas.91.14.6491 10.1073/pnas.91.14.6491 PMC44228 8022810

[B9] Buchmann SL (1983) Buzz pollination in angiosperms. In: Jones CE, Little RJ (Eds) Handbook of Experimental Pollination Biology. Van Nostrand-Reinhold, Pronceton, 73–113.

[B10] Cardale JC (1968) Nest and nesting behaviour of Amegilla (Amegilla) pulchra (Smith) (Hymenoptera: Apoidea: Anthophorinae). Australian Journal of Zoology 16: 689–707. https://doi.org/10.1071/zo9680687

[B11] Cardale JC (1993) Hymenoptera: Apoidea. In: Houston WWK, Maynard GV (Eds) Zoological Catalogue of Australia, Vol. 10. AGPS, Canberra.

[B12] Carsten BC Knowles LL (2007) Shifting distributions and speciation: species divergence during rapid climate change. Molecular Ecology 16: 619–627. https://doi.org/10.1111/j.1365-294X.2006.03167.x 10.1111/j.1365-294X.2006.03167.x 17257117

[B13] Cockerell TDA (1905) Descriptions and Records of Bees-IV. Annals and Magazine of Natural History 16: 392–403. https://doi.org/10.1080/03745480509442880

[B14] Cockerell TDA (1914) Descriptions and Records of Bees-LXIV. Annals and Magazine of Natural History 14: 464–472. https://doi.org/10.1080/00222931408693602

[B15] Cockerell TDA (1926) Descriptions and Records of Bees-CXII. Annals and Magazine of Natural History 18: 216–227. https://doi.org/10.1080/00222932608633504

[B16] Cockerell TDA (1931) The Bees of Australia. Australian Zoologist 7: 34–54.

[B17] Cooper SJB Hinze S Leys R Watts CHS Humphreys WF (2002) Islands under the desert: molecular systematic and evolutionary origins of stygobitic water beetles (Coleoptera: Dytiscidae) from central Western Australia. Invertebrate Systematics 16: 589–598. https://doi.org/10.1071/IT01039

[B18] Dollin A Batley M Robinson M Faulkner B (2000) Native Bees of the Sydney Region, a Field Guide. Richmond: Australian Native Bee Research Centre.

[B19] Drummond AJ Rambaut A (2007) BEAST: Bayesian evolutionary analysis by sampling trees. BMC Evolutionary Biology 7: 214. https://doi.org/10.1186/1471-2148-7-214 10.1186/1471-2148-7-214 PMC2247476 17996036

[B20] Evans N Brown D Corbett GG (2002) The semantics of gender in Mayali: Partially parralel systems and formal implementation. Language 78: 111–155. https://doi.org/10.1353/lan.2002.0014

[B21] Fabricius JC (1758) Systema Naturae per Regna tria Naturae, secundem Classes, Ordines, Genera, Species, cum Characteribus, Differentis, Synonymis, Locis. Tom.1 Editio decima, reformata, Laurentii Salvii, Holmiae.

[B22] Folmer O Black M Hoeh W Lutz R Vrijenhoek R (1994) DNA primers for the amplification of mitochondrial cytochrome c oxidase subunit I from metazoan invertebrates. Molecular Marine Biology and Biotechnology 3: 294–299. 7881515

[B23] Fung KK (2005) Photonic Iridescence of a Blue-banded Bee. Microscopy and Microanalysis 11: 1202–1203. https://doi.org/10.1017/S1431927605500813

[B24] Groom SVC Ngo HT Rehan SM Skelton P Stevens MI Schwarz MP (2014) Multiple recent introductions of apid bees into Pacific archipelagos signify potentially large consequences for both agriculture and indigenous ecosystems. Biol Invasions 16: 2293–2302. https://doi.org/10.1007/s10530-014-0664-7

[B25] Harris RA (1979) A glossary of surface sculpturing. Occasional Papers in Entomology 28: 1–31.

[B26] Hebert PDN Penton EH Burns JM Janzen DH Hallwachs W (2004) Ten species in one: DNA barcoding reveals cryptic species in the neo-tropical skipper butterfly *Astraptes fulgerator* Proceedings National Academy of Sciences, USA 101: 14812–14817. https://doi.org/10.1073/pnas.0406166101 10.1073/pnas.0406166101 PMC522015 15465915

[B27] Hebert PDN deWaard JR Landry JF (2010) DNA barcodes for 1/1000 of the animal kingdom. Biology Letters 6: 359–362. https://doi.org/10.1098/rsbl.2009.0848 10.1098/rsbl.2009.0848 PMC2880045 20015856

[B28] Hogendoorn K Bartholomaeus F Keller MA (2010) Chemical and sensory comparison of tomatoes pollinated by bees and by a pollination wand. Journal of Economic Entomology 103: 1286–1292. https://doi.org/10.1603/EC09393 10.1603/ec09393 20857738

[B29] Hogendoorn K Sedgley M Gross CL Keller MA (2006) Increased tomato yield through pollination by native Australian blue-banded bees (*Amegilla chlorocyanea* Cockerell). Journal of Economic Entomology 99: 828–833. https://doi.org/10.1093/jee/99.3.828 10.1603/0022-0493-99.3.828 16813318

[B30] Houston TF (1975) A revision of the Australian Hylaeine bees (Hymenoptera: Colletidae) I. Australian Journal of Zoology, Supplementary Series 36: 1–135. https://doi.org/10.1071/AJZS036

[B31] Huelsenbeck JP Ronquist F (2001) MRBAYES: Bayesian inference of phylogeny. Bioinformatics 17: 754–755. https://doi.org/10.1093/bioinformatics/17.8.754 10.1093/bioinformatics/17.8.754 11524383

[B32] Jennings WB Edwards SV (2005) Speciational History of Australian Grass Finches (*Poephila*) Inferred from Thirty Gene Trees. Evolution 59: 2033–2047. https://doi.org/10.1111/j.0014-3820.2005.tb01072.x 16261740

[B33] Joseph L Omland KE (2009) Phylogeography: its development and impact in Australo-Papuan ornithology with special reference to paraphyly in Australian birds. Emu 109: 1–23. https://doi.org/10.1071/MU08024

[B34] Kawakami T Butlin RK (2012) Hybrid zones. eLS. https://doi.org/10.1002/9780470015902.a0001752.pub2

[B35] Klopfstein S Kropf C Baur H (2016) *Wolbachia* endosymbionts distort DNA barcoding in the parasitoid wasp genus *Diplazon* (Hymenoptera: Ichneumonidae). Zoological Journal of the Linnean Society (London) 2016: 1–17.

[B36] Lee JL Edwards SV (2008) Divergence across Australia’s Carpentarian Barrier: Statistical Phylogeography of the Red-backed Fairy Wren (*Malurus melanocephalus*). Evolution 62: 3117–3134. https://doi.org/10.1111/j.1558-5646.2008.00543.x 10.1111/j.1558-5646.2008.00543.x 19087188

[B37] Leys R Cooper SJB Schwarz MP (2002) Molecular phylogeny and historical biogeography of the large carpenter bees, genus *Xylocopa* (Hymenoptera: Apidae). Biological Journal of the Linnean Society 77: 249–266. https://doi.org/10.1046/j.1095-8312.2002.00108.x

[B38] Linnæus C (1758) Systema Naturae per Regna tria Naturae, secundem Classes, Ordines, Genera, Species, cum Characteribus, Differentis, Synonymis, Locis, Vol. I (10^th^ edn). Salvius, Holmia.

[B39] Martin HA (2006) Cenozoic climatic change and the development of the arid vegetation in Australia. Journal of Arid Environments 66: 533–563. Michener C D (1944) Comparative external morphology, phylogeny, and a classification of the bees (Hymenoptera). Bulletin of the American Museum of Natural History 82: 151–326.

[B40] Michener CD (1960) Observations on the behaviour of a burrowing bee (*Amegilla*) near Brisbane, Queensland (Hymenoptera, Anthophorinae). Queensland Naturalist 16: 63–67.

[B41] Michener CD (1965) A Classification of the Bees of the Australian and South Pacific Regions. Bulletin of the American Museum of Natural History 130: 1–362.

[B42] Michener CD (2000) The Bees of the World. John Hopkins University Press, Baltimore, 913 pp.

[B43] Pamilo P Viljakainen L Vihavainen A (2007) Exceptionally high density of NUMTs in the honeybee genome. Molecular Biology and Evololution 24: 1340–1346. https://doi.org/10.1093/molbev/msm055 10.1093/molbev/msm055 17383971

[B44] Popov VV (1950) Concerning the genus *Amegilla* Friese (Hymenoptera, Apoidea). Entomologicheskoe Obozrenie 31: 257–261. [In Russian]

[B45] Rambaut A Drummond AJ (2007) Tracer v1.4. http://beast.bio.ed.ac.uk/Tracer

[B46] Rayment T (1935) A Cluster of Bees. Endeavour, Sydney, 752 pp.

[B47] Rayment T (1944) A critical revision of species in the zonata group of *Anthophora* by new characters (Part I). Treubia (Dobutu Gaku-Iho), Hors series (Japanese series), 30 pp.

[B48] Rayment T (1947) A critical revision of species in the zonata group of *Anthophora* by new characters (Part II), Treubia 19: 46–73.

[B49] Rayment T (1951) A critical revision of species in the genus *Asaropoda* by new characters. Memoirs of the Natural History Museum of Victoria 17: 65–80.

[B50] Simon C Frati F Beckenbach A Crespi B Liu H Flook P (1994) Evolution, weighting, and phylogenetic utility of mitochondrial gene sequences and a compilation of conserved polymerase chain reaction primers. Annals of the Entomological Society of America 87: 651–701. https://doi.org/10.1093/aesa/87.6.651

[B51] Smith F (1854) Catalogue of hymenopterous insects in the collection of the British Museum – Part II: Apidae. British Museum, London,198–465.

[B52] Song H Buhay JE Whiting MF Crandall KA (2008) Many species in one: DNA barcoding overestimates the number of species when nuclear mitochondrial pseudogenes are coamplified. Proceedings of the National Academy of Sciences of the United States of America 105: 13486–13491. https://doi.org/10.1073/pnas.0803076105 10.1073/pnas.0803076105 PMC2527351 18757756

[B53] Swofford DL (2001) PAUP*: phylogenetic analysis using parsimony (and other methods). Version 4.0b8. Sunderland, Massachusetts.

[B54] Symon DE (1979) Sex forms in *Solanum* (Solanaceae) and the role of pollen collecting insects. In: Hawkes JG, Lester RN, Skelding AD(Eds) The Biology and Taxonomy of the Solanaceae. Academic Press, London.

[B55] Wallman JF Leys R Hogendoorn K (2005) Molecular systematics of Australian carrion-breeding blowflies (Diptera: Calliphoridae) based on mitochondrial DNA. Invertebrate Systematics 19: 1–15. https://doi.org/10.1071/IS04023

