# Supplementary material for: The genus Amegilla (Hymenoptera, Apidae, Anthophorini) in Australia: A revision of the subgenera Notomegilla and Zonamegilla
Source: Zookeys. 2017 Feb 8;(653):79–140. doi: 10.3897/zookeys.653.11177 (PMC5345376; doi:10.3897/zookeys.653.11177)
Supplement: Supplementary material 1 — Phylogenetic trees and table of DNA voucher numbers, including Genbank accession numbers [file zookeys-653-079-s001.pdf]

Supplementary Material

Associated with

The genus *Amegilla* (Hymenoptera: Apidae: Anthophorini) in Australia: A revision of the subgenera *Notomegilla* and *Zonamegilla* by REMKO LEIJS, MICHAEL BATLEY AND KATJA HOGENDOORN.

Fig. S1.
CO1 (primers M70/M202) neighbour-joining tree calculated using uncorrected sequence divergence in PAUP*. Terminals are labelled with RB-numbers, which refer to Table S1, sequencing primer or 'cons', indicating consensus sequence based on forward and reverse sequencing.

Fig. S2.
CO1 (primers M414/M423) neighbour-joining tree calculated using uncorrected sequence divergence in PAUP*. Terminals are labelled with RB-numbers, which refer to Table S1, sequencing primer or 'cons', indicating consensus sequence based on forward and reverse sequencing.

Table S1. Table of DNA specimens and GenBank accession numbers and locality data.

Figure S1

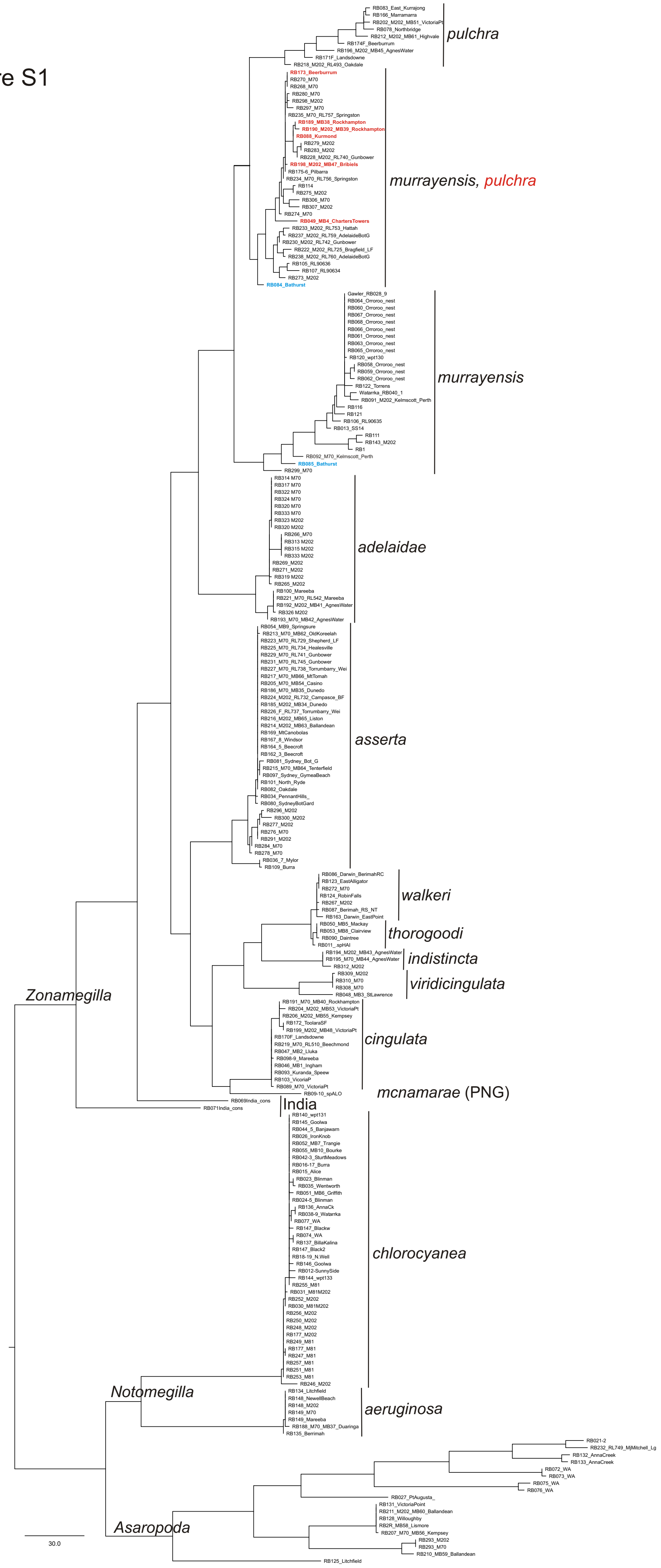
pulchra
murrayensis, pulchra
murrayensis
adelaidae
asserta
walkeri
thorogoodi
indistincta
viridicingulata
cingulata
mcnamarae (PNG)
India
chlorocyanea
aeruginosa
Zonamegilla
Notomegilla
Asaropoda
30.0

Figure S2

Notes about *Amegilla (Zonamegilla) pulchra*:
Specimens in red were collected at Victoria Point, Queensland

Specimens in blue were collected in Brisbane, Queensland

Specimens in green appeared in one of the *murrayensis* clades in the analysis with the M202/M70 primers

Although, variable with respect to colour of face marks and hair bands, the variation did not correlate with the two clades, all these specimens were identified as *pulchra*

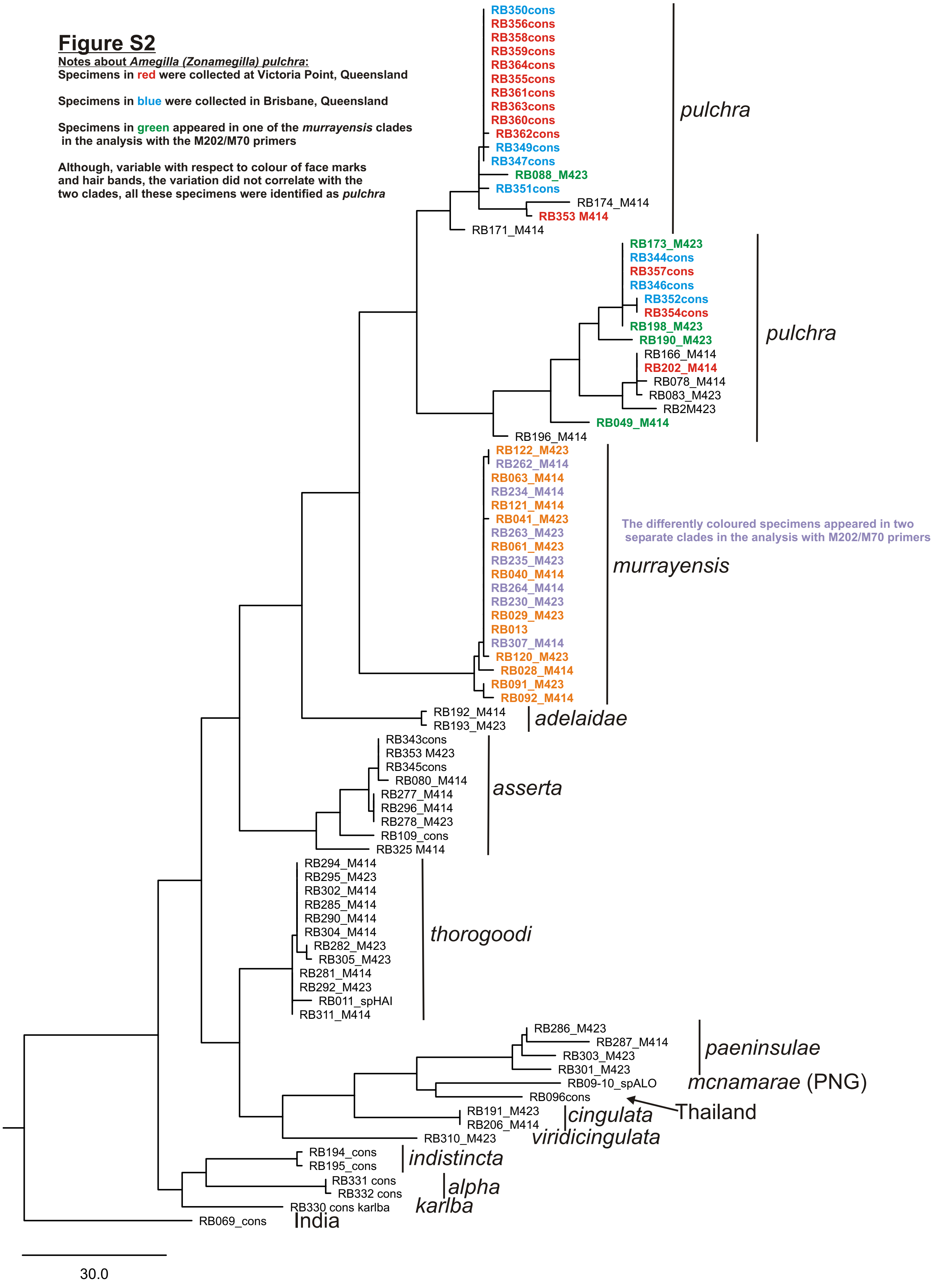

collection	reg prefix	REGNO	DNA number	GenBank Accn M414/M423	GenBank Accn M202/M070	Field No.	subgenus	species	location/site	state	long dec	lat dec lat	date	Leg.
			RB009	x	x		Zon-	mcnamarae	Alotau, Milnebay Prov.	PNG				R.Foster
			RB010	KY485637	KY485773		Zon-	mcnamarae	Alotau, Milnebay Prov.	PNG				R.Foster
			RB011	KY485625	KY485762		Zon-	thorogoodi	Haia Village,	PNG				R.Foster
ABTC		78813	RB013	KY485660	KY485850	RL355	Zon-	murrayensis	Sunny Side, N of Murray Bridge	SA	139.36000	-35.05000	28/12/2003	R.Leijs & K.Hogendoorn
SAM-DNA-Tissue			RB028	KY485661	KY485851	RL326a	Zon-	murrayensis	Gawler, from nests aggregation	SA	138.75000	-34.59000	30/10/2004	R.Leijs
SAM-DNA-Tissue			RB029	KY485662		RL326b	Zon-	murrayensis	Gawler, from nests aggregation	SA	138.75000	-34.59000	30/10/2004	R.Leijs
ABTC			RB040	KY485663	KY485877	RL439	Zon-	murrayensis	Watarrka NP	NT	133.88000	-23.70000	2/05/2005	Chris Brocks
ABTC			RB041	KY485664	x	RL440	Zon-	murrayensis	Watarrka NP	NT	133.88000	-23.70000	11/04/2005	Chris Brocks
AM	K.	290979	RB049	KY485647	KY485878		Zon-	pulchra	120 km S Charters Towers	QLD	146.43333	-21.08333	16/04/2005	M. Batley
ABTC			RB061	KY485665	KY485855	RL474	Zon-	murrayensis	Orroroo ex nest	SA	138.52000	-32.98000	1/10/2005	R.Leijs & K.Hogendoorn
ABTC			RB063	KY485666	KY485857	RL476	Zon-	murrayensis	Orroroo ex nest	SA	138.52000	-32.98000	1/08/2005	R.Leijs & K.Hogendoorn
			RB069	KY485622	KY485757		Zon-	sp.India		India				B. Kranz
ABTC		32-002611	RB078	KY485648	KY485841	RL487	Zon-	pulchra	Northbridge	NSW	151.21667	-33.80000	27/02/2003	M.Bell
ABTC			RB080	KY485640	KY485790	RL489	Zon-	asserta	Sydney Botanic Gardens	NSW	151.20000	-33.85000	31/03/2003	M.Bell
ABTC		32-002612	RB083	KY485649	KY485842	RL494	Zon-	pulchra	East Kurrajong	NSW	150.76667	-33.50000	8/01/2003	R. Spooner-Hart
ABTC			RB088	KY485650	KY485880	RL504	Zon-	pulchra	Kurmond	NSW	150.70000	-33.53333	19/03/2002	M.Bell
ABTC			RB091	KY485667	KY485881	RL520	Zon-	murrayensis	Kelmscott Perth	WA	116.16667	-33.16667	14/01/2003	
ABTC			RB092	KY485668	KY485864	RL521	Zon-	murrayensis	Kelmscott Perth	WA	116.16667	-33.16667	14/01/2003	
ABTC			RB109	KY485641	KY485795	RL574	Zon-	asserta	Burra	SA	138.51855	-33.00263	3/01/2006	R.Leijs & K.Hogendoorn
ABTC			RB120	KY485669	KY485872	RL647	Zon-	murrayensis	Beetalo Valley	SA	138.44434	-33.57023	3/01/2006	R.Leijs & K.Hogendoorn
ABTC			RB121	KY485670	KY485873	RL648	Zon-	murrayensis	Beetalo Valley	SA	138.44434	-33.57023	3/01/2006	R.Leijs & K.Hogendoorn
ABTC			RB122	KY485671	KY485874	RL669	Zon-	murrayensis	Torrens aggr	SA	138.61680	-34.90140	8/01/2006	R.Leijs & K.Hogendoorn
AM	K.	290908	RB166	KY485651	KY485843		Zon-	pulchra	Marramarra NP	NSW	151.06667	-33.55000	14/01/2006	M. Batley
AM	K.	290906	RB171	KY485652	KY485844		Zon-	pulchra	Landsdowne	NSW	152.53333	-31.80000	27/01/2006	M. Batley
AM	K.	290893	RB173	KY485653	KY485882		Zon-	pulchra	Beerburrum	QLD	152.83333	-26.93333	18/04/2006	M. Batley
AM	K.	290894	RB174	KY485654	KY485845		Zon-	pulchra	Beerburrum	QLD	152.83333	-26.93333	18/04/2006	M. Batley
SAM-DNA-Tissue			RB190	KY485655	KY485885	MB39	Zon-	murrayensis	Rockhampton	QLD	150.48333	-23.40000	12/01/2007	M. Batley
SAM-DNA-Tissue			RB191	KY485638	KY485782	MB40	Zon-	cingulata	Rockhampton	QLD	150.48333	-23.40000	12/01/2007	M. Batley
AM	K.	290885	RB193	KY485646	KY485824		Zon-	adelaidae	Agnes Water 23 km SW	QLD	151.93333	-24.35000	13/01/2007	M. Batley
AM	K.	290886	RB194	KY485679	KY485907		Zon-	indistincta	Agnes Water 23 km SW	QLD	151.93333	-24.35000	13/01/2007	M. Batley
AM	K.	290887	RB195	KY485680	KY485908		Zon-	indistincta	Agnes Water 23 km SW	QLD	151.93333	-24.35000	13/01/2007	M. Batley
AM	K.	290888	RB196	KY485656	KY485846		Zon-	pulchra	Agnes Water 23 km SW	QLD	151.93333	-24.35000	13/01/2007	M. Batley
AM	K.	290896	RB198	KY485657	KY485886		Zon-	pulchra	Bribie Is	QLD	153.15000	-27.05000	15/01/2007	M. Batley
/I+SAM-DNA-Tissue	K.	290922	RB202	KY485658	KY485847	MB51	Zon-	pulchra	Victoria Point	QLD	153.30000	-27.56667	16/01/2007	M. Batley
AM	K.	290904	RB206	KY485639	KY485785		Zon-	cingulata	Kempsey	NSW	152.83333	-31.10000	17/01/2007	M. Batley
ABTC			RB230	KY485672	KY485889	RL742	Zon-	murrayensis	Gunbower	VIC	144.34512	-35.90349	6/01/2007	R.Leijs & K.Hogendoorn
ABTC			RB234	KY485673	KY485891	RL756	Zon-	murrayensis	Springston	SA	139.08935	-34.70785	8/01/2007	R.Leijs
ABTC			RB235	KY485674	KY485892	RL757	Zon-	murrayensis	Springston	SA	139.08935	-34.70785	8/01/2007	R.Leijs
ABTC		32-002572	RB277	KY485642	KY485816	RL777	Zon-	asserta	S of Coen	QLD	143.19888	-14.04284	27/06/2007	R. Leijs & M. Batley
ABTC		32-002571	RB278	KY485643	KY485817	RL778	Zon-	asserta	S of Coen	QLD	143.19888	-14.04284	27/06/2007	R. Leijs & M. Batley
ABTC			RB296	KY485644	KY485820	RL822	Zon-	asserta	Capt Billies Landing	QLD	142.85619	-11.63194	30/06/2007	R. Leijs & M. Batley
SAM-DNA-Tissue		32-002587	RB307	KY485678	KY485906	RL854	Zon-	murrayensis	Wenlock River X-ing	QLD	142.94161	-13.09570	2/07/2007	R. Leijs & M. Batley
ABTC		32-002626	RB310	KY485624	KY485761	RL860	Zon-	viridicingulata	Cooktown	QLD	145.24130	-15.48977	3/07/2007	R. Leijs & M. Batley
			RB096	KY485681	KY485915		Zon-	sp. Thailand		Thaila				
AM	K.	290884	RB192	KY485645	KY485823		Zon-	adelaidae	Agnes Water 23 km SW	QLD	151.93333	-24.35000	13/01/2007	M. Batley
ABTC			RB262	KY485675		RL767	Zon-	murrayensis	W9.3 Katja's kweek	SA	138.44434	-33.57023	17/03/2007	K.Hogendoorn
ABTC			RB263	KY485676		RL768	Zon-	murrayensis	BY22 Katja's kweek	SA	138.44434	-33.57023	17/03/2007	K.Hogendoorn
ABTC			RB264	KY485677		RL769	Zon-	murrayensis	Katja's kweek	SA	138.44434	-33.57023	17/03/2007	K.Hogendoorn
ABTC		32-002608	RB281	KY485626		RL784	Zon-	thorogoodi	Coen	QLD	143.20022	-13.94415	27/06/2007	R. Leijs & M. Batley
ABTC		32-002607	RB282	KY485627		RL786	Zon-	thorogoodi	Archer River X-ing	QLD	142.94516	-13.43489	27/06/2007	R. Leijs & M. Batley
SAM-DNA-Tissue		32-002606	RB285	KY485628		RL792	Zon-	thorogoodi	N of Bamaga	QLD	142.42316	-10.84117	28/06/2007	R. Leijs & M. Batley
ABTC		32-002619	RB286	KY485682		RL793	Zon-	paeninsulae	N of Bamaga	QLD	142.42316	-10.84117	28/06/2007	R. Leijs & M. Batley
ABTC			RB287	KY485683		RL795	Zon-	paeninsulae	N of Bamaga	QLD	142.42316	-10.84117	28/06/2007	R. Leijs & M. Batley
ABTC		32-002600	RB290	KY485629		RL801	Zon-	thorogoodi	N of Bamaga	QLD	142.42316	-10.84117	28/06/2007	R. Leijs & M. Batley
ABTC		32-002598	RB292	KY485630		RL806	Zon-	thorogoodi	N of Bamaga	QLD	142.42316	-10.84117	28/06/2007	R. Leijs & M. Batley
ABTC		32-002605	RB294	KY485631		RL816	Zon-	thorogoodi	near Jardine's Well	QLD	142.60858	-10.75012	29/06/2007	R. Leijs & M. Batley
SAM-DNA-Tissue		32-002604	RB295	KY485632		RL817	Zon-	thorogoodi	near Jardine's Well	QLD	142.60858	-10.75012	29/06/2007	R. Leijs & M. Batley
ABTC		32-002620	RB301	KY485684		RL837	Zon-	paeninsulae	Iron Range	QLD	143.23521	-12.74302	1/07/2007	R. Leijs & M. Batley
SAM-DNA-Tissue		32-002603	RB302	KY485633		RL838	Zon-	thorogoodi	Iron Range	QLD	143.23521	-12.74302	1/07/2007	R. Leijs & M. Batley
SAM-DNA-Tissue		32-002622	RB303	KY485685		RL846	Zon-	paeninsulae	Iron Range	QLD	143.25562	-12.74050	1/07/2007	R. Leijs & M. Batley
SAM-DNA-Tissue		32-002602	RB304	KY485634		RL848	Zon-	thorogoodi	Iron Range	QLD	143.25562	-12.74050	1/07/2007	R. Leijs & M. Batley
SAM-DNA-Tissue		32-002601	RB305	KY485635		RL851	Zon-	thorogoodi	Iron Range	QLD	143.25562	-12.74050	1/07/2007	R. Leijs & M. Batley
SAM-DNA-Tissue		32-002599	RB311	KY485636		RL865	Zon-	thorogoodi	Bloomfield nr rubbish tip	QLD	145.34161	-15.90114	3/07/2007	R. Leijs & M. Batley
AM	K.	290956	RB325	KY485686			Zon-	asserta	40 Mile Scrub		144.83167	-18.10694	17/05/2008	M. Batley
AM	K.	290928	RB330	KY485687			Zon-	karlba	Litchfield NP	NT	130.78333	-13.11667	19-22/4/08	G. Williams & W. Pulawski
AM	K.	278565	RB331	KY485688			Zon-	alpha	175 km SW Katherine (Victoria river roadhouse?)	NT	131.13300	-15.61600	9/04/2008	G. Williams & W. Pulawski
AM	K.	278566	RB332	KY485689			Zon-	alpha	175 km SW Katherine (Victoria river roadhouse?)	NT	131.13300	-15.61600	9/04/2008	G. Williams & W. Pulawski
ABTC			RB343	KY485694			Zon-	asserta	Brisbane, Mt Coot-tha Botanical Gardens	QLD	152.97690	-27.47626	7/11/2013	R.Leijs & K.Hogendoorn
ABTC			RB344	KY485690			Zon-	pulchra	Brisbane, Mt Coot-tha Botanical Gardens	QLD	152.97690	-27.47626	7/11/2013	R.Leijs & K.Hogendoorn
ABTC			RB345	KY485698			Zon-	asserta	Brisbane, Mt Coot-tha Botanical Gardens	QLD	152.97690	-27.47626	7/11/2013	R.Leijs & K.Hogendoorn
ABTC			RB346	KY485699			Zon-	pulchra	Brisbane, West End, Montague Rd	QLD	152.99951	-27.48903	7/11/2013	R.Leijs & K.Hogendoorn
ABTC			RB347	KY485700			Zon-	pulchra	Brisbane, West End, Montague Rd	QLD	152.99951	-27.48903	7/11/2013	R.Leijs & K.Hogendoorn
ABTC			RB349	KY485693			Zon-	pulchra	Brisbane, West End, Vulture Rd	QLD	153.01960	-27.48201	7/11/2013	R.Leijs & K.Hogendoorn
ABTC			RB350	KY485691			Zon-	pulchra	Brisbane, West End, Vulture Rd	QLD	153.01960	-27.48201	7/11/2013	R.Leijs & K.Hogendoorn
ABTC			RB351	KY485708			Zon-	pulchra	Brisbane, West End, Vulture Rd	QLD	153.01960	-27.48201	7/11/2013	R.Leijs & K.Hogendoorn
ABTC			RB352	KY485705			Zon-	pulchra	Brisbane, West End, Vulture Rd	QLD	153.01960	-27.48201	7/11/2013	R.Leijs & K.Hogendoorn
ABTC			RB353	KY485709			Zon-	asserta	Victoria Point, John Klampp	QLD	153.01960	-27.48201	7/11/2013	R.Leijs & K.Hogendoorn
ABTC			RB354	KY485706			Zon-	pulchra	Victoria Point, John Klampp	QLD	153.30141	-27.57784	7/11/2013	R.Leijs & K.Hogendoorn
ABTC			RB355	KY485701			Zon-	pulchra	Victoria Point, John Klampp	QLD	153.30141	-27.57784	7/11/2013	R.Leijs & K.Hogendoorn
ABTC			RB356	KY485711			Zon-	pulchra	Victoria Point, John Klampp	QLD	153.30141	-27.57784	7/11/2013	R.Leijs & K.Hogendoorn
ABTC			RB357	KY485692			Zon-	pulchra	Victoria Point, John Klampp	QLD	153.30141	-27.57784	7/11/2013	R.Leijs & K.Hogendoorn
ABTC			RB358	KY485695			Zon-	pulchra	Victoria Point, John Klampp	QLD	153.30141	-27.57784	7/11/2013	R.Leijs & K.Hogendoorn
ABTC			RB360	KY485707			Zon-	pulchra	Victoria Point, John Klampp	QLD	153.30141	-27.57784	7/11/2013	R.Leijs & K.Hogendoorn
ABTC			RB361	KY485702			Zon-	pulchra	Victoria Point, John Klampp	QLD	153.30141	-27.57784	7/11/2013	R.Leijs & K.Hogendoorn
ABTC			RB362	KY485703			Zon-	pulchra	Victoria Point, John Klampp	QLD	153.30141	-27.57784	7/11/2013	R.Leijs & K.Hogendoorn
ABTC			RB363	KY485704			Zon-	pulchra	Victoria Point, John Klampp	QLD	153.30141	-27.57784	7/11/2013	R.Leijs & K.Hogendoorn
ABTC			RB364	KY485697			Zon-	pulchra	Victoria Point, John Klampp	QLD	153.30141	-27.57784	7/11/2013	R.Leijs & K.Hogendoorn
ABTC		78812	RB012		KY485722	RL354	Not-	chlorocyanea	Sunny Side, N of Murray Bridge	SA	139.36000	-35.05000	28/12/2003	R.Leijs & K.Hogendoorn
ABTC		78815	RB015		KY485719	RL336	Not-	chlorocyanea	Alice Springs Olive Pink Botanic Gardens	NT	133.88000	-23.70000	2/10/2003	K.Hogendoorn
ABTC		78816	RB016		KY485720	RL322	Not-	chlorocyanea	Barina Creek	SA	139.08000	-33.67000	7/10/2003	R.Leijs
ABTC		78817	RB017		x	RL353	Not-	chlorocyanea	Barina Creek	SA	139.08000	-33.67000	7/10/2003	R.Leijs
ABTC		78818	RB018		KY485721	RL350	Not-	chlorocyanea	North Well	SA	135.51667	-31.40000	24/06/2005	R.Leijs & D. Piani
ABTC		78819	RB019		x	RL351	Not-	chlorocyanea	North Well	SA	135.51667	-31.40000	24/06/2005	R.Leijs & D. Piani
SAM-DNA-Tissue			RB023		KY485724	RL126	Not-	chlorocyanea	Blinman Amegilla on Eremophila	SA	138.67798	-31.09357	27/01/2005	R.Leijs
SAM-DNA-Tissue			RB024		KY485725	RL130	Not-	chlorocyanea	Second Spring Amegilla on Melaleuca	SA	138.58278	-31.03159	27/01/2005	R.Leijs
SAM-DNA-Tissue			RB025		x	RL131	Not-	chlorocyanea	Rawnsley Park Amegilla on Eremophila	SA	138.59380	-31.65085	28/01/2005	R.Leijs
			RB026		KY485726		Not-	chlorocyanea	Iron Nob	SA	137.2925	-32.6570	2-Oct-04	M.Batley
SAM-DNA-Tissue			RB030		KY485727	RL367	Not-	chlorocyanea	10 km SW Heywood	VIC	141.59000	-38.19000	15/12/2003	R.Leijs & K.Hogendoorn
			RB031		KY485728		Not-	chlorocyanea	11 km SW Heywood	VIC	141.59000	-38.19000	16/12/2003	R.Leijs & K.Hogendoorn
SAM-DNA-Tissue			RB034		KY485788	RL377	Zon-	asserta	Pennant Hills	NSW	151.06667	-33.73333	17/12/2003	M. Batley
AM			RB035		KY485743		Not-	chlorocyanea	5 km N Wentworth	NSW	141.56000	-34.04000	5/01/2004	M. Batley
SAM-DNA-Tissue		32-002579	RB036		KY485787	RL392	Zon-	asserta	Mylor, Nursery Bradburry Rd	SA	138.73000	-35.05000	28/02/2004	R.Leijs & K.Hogendoorn

SAM-DNA-Tissue		2-002580-81	RB037		x	RL393	Zon-	asserta	Mylor, Nursery Bradburry Rd	SA	138.73000	-35.05000	28/02/2004	R.Leijs & K.Hogendoorn
SAM-DNA-Tissue			RB038		KY485742	RL436	Not-	chlorocyanea	Watarrka NP	NT	133.88000	-23.70000	11/04/2005	Chris Brocks
SAM-DNA-Tissue			RB039		x	RL437	Not-	chlorocyanea	Watarrka NP	NT	133.88000	-23.70000	11/04/2005	Chris Brocks
SAM-DNA-Tissue			RB042		KY485741	RL447	Not-	chlorocyanea	Katherine 180 km SW	WA	120.96998	-28.67735	26/04/2005	R.Leijs
SAM-DNA-Tissue			RB043		x	RL448	Not-	chlorocyanea	Katherine 180 km SW	WA	120.96998	-28.67735	26/04/2005	R.Leijs
SAM-DNA-Tissue			RB044		KY485723	RL452	Not-	chlorocyanea	Banjawarn HS	WA	121.61354	-27.70531	5/05/2005	R.Leijs
SAM-DNA-Tissue			RB045		x	RL453	Not-	chlorocyanea	Banjawarn HS	WA	121.61354	-27.70531	5/05/2005	R.Leijs
AM	K.	290999	RB046		KY485774		Zon-	cingulata	Ingham	QLD	146.18333	-18.53333	18/04/2005	M. Batley
AM	K.	290903	RB047		KY485775		Zon-	cingulata	Iluka	NSW	153.35000	-29.40000	26/04/2005	M. Batley
AM	K.	290978	RB048		KY485758		Zon-	viridicingulata	40 km S St Lawrence	QLD	149.51667	-22.48333	20/04/2005	M. Batley
AM	K.	290877	RB050		KY485763		Zon-	thorogoodi	50 km N Mackay	QLD	148.80000	-20.91667	19/04/2005	M. Batley
AM	K.	290875	RB051		KY485729		Not-	chlorocyanea	30 km N Griffith	NSW	146.10000	-34.13333	8/10/2004	M. Batley
AM	K.	290980	RB052		KY485730		Not-	chlorocyanea	16 km NE Trangie	NSW	145.96667	-31.93333	12/04/2005	M. Batley
AM	K.	290898	RB053		KY485764		Zon-	thorogoodi	Clairview	QLD	149.53333	-22.11667	20/04/2005	M. Batley
AM	K.	290982	RB054		KY485789		Zon-	asserta	17 km SE Springsure	QLD	148.20000	-24.21667	15/04/2005	M. Batley
AM	K.	290876	RB055		KY485731		Not-	chlorocyanea	45 km N Bourke	NSW	145.96667	-29.75000	13/04/2005	M. Batley
ABTC			RB058		KY485852	RL471	Zon-	murrayensis	Orroroo ex nest	SA	138.52000	-32.98000	1/10/2005	R.Leijs & K.Hogendoorn
ABTC			RB059		KY485853	RL472	Zon-	murrayensis	Orroroo ex nest	SA	138.52000	-32.98000	1/10/2005	R.Leijs & K.Hogendoorn
ABTC			RB060		KY485854	RL473	Zon-	murrayensis	Orroroo ex nest	SA	138.52000	-32.98000	19/10/2006	R.Leijs & K.Hogendoorn
ABTC			RB062		KY485856	RL475	Zon-	murrayensis	Orroroo ex nest	SA	138.52000	-32.98000	1/08/2005	R.Leijs & K.Hogendoorn
ABTC			RB064		KY485858	RL477	Zon-	murrayensis	Orroroo ex nest	SA	138.52000	-32.98000	1/08/2005	R.Leijs & K.Hogendoorn
ABTC			RB065		KY485859	RL478	Zon-	murrayensis	Orroroo ex nest	SA	138.52000	-32.98000	1/08/2005	R.Leijs & K.Hogendoorn
ABTC			RB067		KY485861	RL480	Zon-	murrayensis	Orroroo ex nest	SA	138.52000	-32.98000	1/08/2005	R.Leijs & K.Hogendoorn
SAM-DNA-Tissue			RB068		KY485862	RL481	Zon-	murrayensis	Orroroo ex nest	SA	138.52000	-32.98000	1/08/2005	R.Leijs & K. Hogendoorn
			RB070		x		Zon-	sp.India		India				
ABTC			RB074		KY485732	RL461	Not-	chlorocyanea		WA	116.96560	-27.04085	19/10/2005	R.Leijs
ABTC			RB077		KY485733	RL464	Not-	chlorocyanea	Avenong Well Hillview Stn	WA	119.11432	-27.22518	21/10/2006	R.Leijs
ABTC		32-002576	RB081		KY485791	RL490	Zon-	asserta	Sydney Botanic Gardens	NSW	151.20000	-33.85000	31/03/2003	M.Bell
ABTC		32-002578	RB082		KY485792	RL492	Zon-	asserta	Oakdale	NSW	150.50000	-34.06667	23/02/2002	M.Eden
ABTC			RB084		KY485879	RL495	Zon-	murrayensis	Bathurst	NSW	149.71667	-33.81667	11/01/2003	M.Bell
ABTC			RB085		KY485863	RL496	Zon-	murrayensis	Bathurst	NSW	149.71667	-33.81667	11/01/2003	M.Bell
ABTC			RB086		KY485766	RL499	Zon-	walkeri	Berimah Researh Farm Orchard	NT	130.91667	-12.43333	14/05/2003	G.R. Brown & H Wallace
ABTC		32-002597	RB087		KY485767	RL500	Zon-	walkeri	Berimah Researh Farm Orchard	NT	130.91667	-12.43333	14/05/2003	G.R. Brown & H Wallace
ABTC			RB089		KY485776	RL513	Zon-	cingulata	Victoria Point	QLD	153.31667	-27.58333	16/04/2003	J. Klumpp
ABTC		32-002609	RB090		KY485765	RL515	Zon-	thorogoodi	Daintree	QLD	145.31667	-16.28333	28/10/2003	W. Forno
ABTC			RB093		KY485777	RL522	Zon-	cingulata	Kurranda-Speewah	QLD	145.61667	-16.88333	19/04/2003	
ABTC		32-002577	RB097		KY485793	RL537	Zon-	asserta	Gymea Beach, Sydney	NSW	151.10100	-34.04900	13/04/2003	G.Hambridge
ABTC			RB098		KY485778	RL538	Zon-	cingulata	8K SE Mareeba	QLD	145.41667	-16.98333	14/04/2003	R. Storey
ABTC			RB099		x	RL540	Zon-	cingulata	8K SE Mareeba	QLD	145.41667	-16.98333	14/04/2003	R. Storey
ABTC		32-002618	RB100		KY485822	RL543	Zon-	adelaidae	8K SE Mareeba	QLD	145.41667	-16.98333	14/04/2003	R. Storey
ABTC			RB101		KY485794	RL546	Zon-	asserta	North Ryde	NSW	151.12200	-33.79600	1/01/2003	
ABTC			RB103		KY485779	RL549	Zon-	cingulata	Victoria Point	QLD	153.31667	-27.58333	13/04/2003	J. Klumpp
ABTC			RB105		KY485865	RL553	Zon-	murrayensis		SA	138.61858	-32.63653	3/01/2006	R.Leijs & K.Hogendoorn
ABTC			RB106		KY485866	RL556	Zon-	murrayensis		SA	138.61858	-32.63653	3/01/2006	R.Leijs & K.Hogendoorn
ABTC			RB107		KY485867	RL569	Zon-	murrayensis		SA	138.51989	-35.44167	3/01/2006	R.Leijs & K.Hogendoorn
ABTC			RB111		KY485868	RL577	Zon-	murrayensis		SA	138.59848	-32.63877	3/01/2006	R.Leijs & K.Hogendoorn
ABTC			RB114		KY485869	RL599	Zon-	murrayensis		SA	139.00634	-33.63083	3/01/2006	R.Leijs & K.Hogendoorn
ABTC			RB116		KY485870	RL614	Zon-	murrayensis		SA	139.08148	-33.66785	3/01/2006	R.Leijs & K.Hogendoorn
ABTC			RB117		KY485871	RL616	Zon-	murrayensis		SA	139.08148	-33.66785	3/01/2006	R.Leijs & K.Hogendoorn
ABTC		32-002595	RB123		KY485768	RL695	Zon-	walkeri	East Alligator Ranger Station	NT	132.95700	-12.41100	7/03/2006	D.A.Young
ABTC		32-002594	RB124		KY485769	RL698	Zon-	walkeri	Robin Falls nr. Adelaide River	NT	131.13780	-13.34967	4/03/2006	D.A.Young
SAM-DNA-Tissue			RB134		KY485910	RL705	Not-	aeruginosa	Litchfield	NT	130.69900	-13.48100	18/02/2006	D.A.Young
SAM-DNA-Tissue			RB135		KY485911	RL697	Not-	aeruginosa	Darwin, Berrimah Farm	NT	130.93300	-12.44400	18/02/2006	D.A.Young
SAM-DNA-Tissue			RB136		KY485734	RL677	Not-	chlorocyanea	Anna Creek Stn Marpoo Dam	SA	135.81341	-29.08262	6/04/2006	R.Leijs
SAM-DNA-Tissue			RB137		KY485735	RL673	Not-	chlorocyanea	Billa Kalina HS	SA	136.18851	-29.91676	1/04/2006	R.Leijs
ABTC			RB140		KY485736	RL667	Not-	chlorocyanea		SA	138.22822	-33.24223	3/01/2006	R.Leijs & K.Hogendoorn
ABTC			RB143		KY485875	RL570	Zon-	murrayensis		SA	138.52302	-32.97012	3/01/2006	R.Leijs & K.Hogendoorn
ABTC			RB144		KY485737	RL572	Not-	chlorocyanea		SA	138.53487	-32.73402	3/01/2006	R.Leijs & K.Hogendoorn
SAM-DNA-Tissue			RB145		KY485738	RL684	Not-	chlorocyanea	Goolwa Sth Bike track	SA	138.77900	-35.51200	2/04/2006	D.A.Young
SAM-DNA-Tissue			RB146		KY485739	RL685	Not-	chlorocyanea	Goolwa Sth Bike track	SA	138.77900	-35.51200	2/04/2006	D.A.Young
ABTC			RB147		KY485740	RL671	Not-	chlorocyanea	4.4 km N of Blackwood Norgate Nursery	VIC	144.27996	-37.44075	25/01/2006	R.Leijs & K.Hogendoorn
ABTC			RB148		KY485912	RL526	Not-	aeruginosa	Newell Beach	QLD	145.40000	-16.41667	27/10/2003	W. Forno
ABTC			RB149		KY485913	RL531	Not-	aeruginosa	8K SE Mareeba	QLD	145.41667	-16.98333	24/04/2003	R. Storey
AM	K.	290889	RB162		KY485796		Zon-	asserta	Beecroft	NSW	151.06667	-33.73333	2/11/2005	M. Batley
AM	K.	290890	RB163		KY485770		Zon-	asserta	Beecroft	NSW	151.06667	-33.73333	22/11/2005	M. Batley
AM	K.	290878	RB167		KY485798		Zon-	asserta	55 km N of Windsor	NSW	150.66667	-33.23333	22/02/2006	M. Batley
AM	K.	290910	RB169		KY485799		Zon-	asserta	Mt Canobolas	NSW	148.98333	-33.33333	19/01/2006	M. Batley
AM	K.	290905	RB170		KY485780		Zon-	cingulata	Landsdowne	NSW	152.53333	-31.80000	27/01/2006	M. Batley
SAM-DNA-Tissue			RB172		KY485781	MB21	Zon-	cingulata	Toolara SF	QLD	152.80000	-26.01667	22/01/2006	M. Batley
SAM-DNA-Tissue			RB175		KY485883	MB24	Zon-	murrayensis	Pilbara	WA	117.90000	-22.35000	30/09/2004	M. Batley
AM	K.	290940	RB176		KY485744		Zon-	murrayensis	Pilbara	WA	117.90000	-22.35000	30/09/2004	M. Batley
AM	K.	290900	RB185		KY485800		Zon-	asserta	Dunedoo 15 km NW	NSW	149.31667	-31.91667	9/01/2007	M. Batley
AM	K.	290901	RB186		KY485801		Zon-	asserta	Dunedoo 15 km NW	NSW	149.31667	-31.91667	9/01/2007	M. Batley
AM	K.	290899	RB188		KY485914		Not-	aeruginosa	Duaringa	QLD	149.66667	-23.71667	12/01/2007	M. Batley
SAM-DNA-Tissue			RB189		KY485884	MB38	Zon-	pulchra	Rockhampton	QLD	150.48333	-23.40000	12/01/2007	M. Batley
SAM-DNA-Tissue			RB199		KY485783	MB48	Zon-	cingulata	Victoria Point	QLD	153.30000	-27.58333	16/01/2007	M. Batley
SAM-DNA-Tissue			RB204		KY485784	MB53	Zon-	cingulata	Victoria Point	QLD	153.30000	-27.56667	16/01/2007	M. Batley
AM	K.	290897	RB205		KY485802		Zon-	asserta	Casino 5 km N	NSW	153.03333	-28.81667	17/01/2007	M. Batley
AM	K.	290902	RB212	KY485659	KY485848		Zon-	pulchra	Highvale	QLD	152.86667	-27.38333	26/01/2007	M. Batley
AM	K.	290913	RB213		KY485803		Zon-	asserta	Old Koreelah	QLD	152.41667	-28.40000	26/01/2007	M. Batley
AM	K.	290983	RB214		KY485804		Zon-	asserta	Tenterfield 11 km NW	NSW	151.93830	-28.97750	24/01/2007	M. Batley
SAM-DNA-Tissue			RB215		KY485805	MB64	Zon-	asserta	Tenterfield, 15 km N	NSW	151.93333	-28.98333	24/01/2007	M. Batley
AM	K.	290907	RB216		KY485806		Zon-	asserta	Liston	NSW	152.03333	-28.65000	24/01/2007	M. Batley
AM	K.	290981	RB217		KY485807		Zon-	asserta	MountTomah	NSW	150.41667	-33.53333	10/01/2007	M. Batley
ABTC			RB219		KY485786	RL510	Zon-	cingulata	Southwest rd, Beechmont 4211	QLD	153.20000	-28.10000	8/04/2003	W. Forno
ABTC		32-002615	RB221		KY485825	RL542	Zon-	adelaidae	8K SE Mareeba	QLD	145.41667	-16.98333	14/04/2003	R. Storey
ABTC			RB222		KY485887	RL725	Zon-	murrayensis	Bragfield Lavender Farm near Wellington	SA	139.40884	-35.30909	2/01/2007	R.Leijs & K.Hogendoorn
ABTC			RB223		KY485808	RL729	Zon-	asserta	Lavender Farm Shepherds Flat	VIC	144.10872	-37.27407	4/01/2007	R.Leijs & K.Hogendoorn
ABTC			RB224		KY485809	RL732	Zon-	asserta	Berry Farm Campaspe River near Woodend	VIC	144.47917	-37.33420	4/01/2007	R.Leijs & K.Hogendoorn
ABTC			RB225		KY485810	RL734	Zon-	asserta	Healesville	VIC	145.51303	-37.67476	5/01/2007	R.Leijs & K.Hogendoorn
ABTC			RB226		KY485811	RL737	Zon-	asserta	Torrumbarry Weir	VIC	144.46455	-35.94369	6/01/2007	R.Leijs & K.Hogendoorn
ABTC			RB227		KY485812	RL738	Zon-	asserta	Torrumbarry Weir	VIC	144.46455	-35.94369	6/01/2007	R.Leijs & K.Hogendoorn
ABTC			RB228		KY485888	RL740	Zon-	murrayensis	Gunbower	VIC	144.34512	-35.90349	6/01/2007	R.Leijs & K.Hogendoorn
ABTC			RB229		KY485813	RL741	Zon-	asserta	Gunbower	VIC	144.34512	-35.90349	6/01/2007	R.Leijs & K.Hogendoorn
ABTC			RB231		KY485814	RL745	Zon-	asserta	Gunbower	VIC	144.34512	-35.90349	6/01/2007	R.Leijs & K.Hogendoorn
ABTC			RB233		KY485890	RL753	Zon-	murrayensis	Hatah	VIC	142.33509	-34.70578	7/01/2007	R.Leijs & K.Hogendoorn
ABTC			RB237		KY485893	RL759	Zon-	murrayensis	Adelaide Botanical Gardens	SA	138.60896	-34.60896	11/01/2007	R.Leijs & K.Hogendoorn
ABTC			RB238		KY485894	RL760	Zon-	murrayensis	Adelaide Botanical Gardens	SA	138.60896	-34.60896	11/01/2007	R.Leijs & K.Hogendoorn
ABTC			RB246		KY485745	RL723	Not-	chlorocyanea	Bragfield Lavender Farm near Wellington	SA	139.40884	-35.30909	2/01/2007	R.Leijs & K.Hogendoorn
ABTC			RB247		KY485746	RL724	Not-	chlorocyanea	Bragfield Lavender Farm near Wellington	SA	139.40884	-35.30909	2/01/2007	R.Leijs & K.Hogendoorn
ABTC			RB248		KY485747	RL726	Not-	chlorocyanea	TowerHill Lavender Farm	VIC	142.34384	-38.31534	3/01/2007	R.Leijs & K.Hogendoorn
ABTC			RB249		KY485748	RL728	Not-	chlorocyanea	Lavender Farm Shepherds Flat	VIC	144.10872	-37.27407	4/01/2007	R.Leijs & K.Hogendoorn
ABTC			RB250		KY485749	RL731	Not-	chlorocyanea	Berry Farm Campaspe River near Woodend	VIC	144.47917	-37.33420	4/01/2007	R.Leijs & K.Hogendoorn
ABTC			RB251		KY485750	RL733	Not-	chlorocyanea	Healesville	VIC	145.51303	-37.67476	5/01/2007	R.Leijs & K.Hogendoorn
ABTC			RB253		KY485752	RL736	Not-	chlorocyanea	Torrumbarry Weir	VIC	144.46455	-35.94369	6/01/2007	R.Leijs & K.Hogendoorn
ABTC			RB255		KY485753	RL748	Not-	chlorocyanea	Kerang	VIC	143.91470	-35.72849	6/01/2007	R.Leijs & K.Hogendoorn
ABTC			RB256		KY485754	RL751	Not-	chlorocyanea	Boundary Bend	VIC	143.14996	-34.71584	7/01/2007	R.Leijs & K.Hogendoorn
ABTC			RB257		KY485755	RL752	Not-	chlorocyanea	Robinvale	VIC	142.77147	-34.58751	7/01/2007	R.Leijs & K.Hogendoorn
SAM-DNA-Tissue		32-002617	RB265		KY485826	RL501	Zon-	adelaidae	Berimah Researh Farm Orchard	NT	130.91667	-12.43333	14/05/2003	G.R. Brown & H Wallace
ABTC			RB267		KY485771	RL689	Zon-	walkeri	Darwin, Berrimah CSIRO	NT	130.92000	-12.40867	7/05/2006	D.A.Young
ABTC			RB268		KY485895	RL691	Zon-	murrayensis	Darwin, Berrimah CSIRO	NT	130.92000	-12.40867	9/05/2006	D.A.Young

SAM-DNA-Tissue		32-002613	RB269		KY485828	RL693	Zon-	adelaidae	Darwin, Berrimah CSIRO	NT	130.92000	-12.40867	9/05/2006	D.A.Young
ABTC			RB270		KY485896	RL694	Zon-	murrayensis	Darwin, Berrimah CSIRO	NT	130.92000	-12.40867	9/05/2006	D.A.Young
SAM-DNA-Tissue		32-002614	RB271		KY485829	RL703	Zon-	adelaidae	Darwin, Berrimah Farm	NT	130.93300	-12.44400	19/02/2006	D.A.Young
ABTC			RB272		KY485772	RL710	Zon-	walkeri	Darwin, East Point	NT	130.83000	-12.41300	19/02/2006	D.A.Young
ABTC		32-002592	RB273		KY485897	RL770	Zon-	murrayensis	Musgrave	QLD	143.50382	-14.78398	27/06/2007	R. Leijs & M. Batley
ABTC		32-002585	RB274-5		KY485898	RL773	Zon-	murrayensis	Musgrave	QLD	143.50382	-14.78398	27/06/2007	R. Leijs & M. Batley
ABTC		32-002575	RB276		KY485815	RL776	Zon-	asserta	S of Coen	QLD	143.19888	-14.04284	27/06/2007	R. Leijs & M. Batley
SAM-DNA-Tissue		32-002586	RB279		KY485900	RL782	Zon-	murrayensis	Coen	QLD	143.20022	-13.94415	27/06/2007	R. Leijs & M. Batley
SAM-DNA-Tissue		32-002584	RB280		KY485901	RL783	Zon-	murrayensis	Coen	QLD	143.20022	-13.94415	27/06/2007	R. Leijs & M. Batley
ABTC		32-002588	RB283		KY485902	RL787	Zon-	murrayensis	Archer River X-ing	QLD	142.94516	-13.43489	27/06/2007	R. Leijs & M. Batley
ABTC		32-002570	RB284		KY485818	RL788	Zon-	asserta	Archer River X-ing	QLD	142.94516	-13.43489	27/06/2007	R. Leijs & M. Batley
SAM-DNA-Tissue		32-002574	RB291		KY485819	RL803	Zon-	asserta	N of Bamaga	QLD	142.42316	-10.84117	28/06/2007	R. Leijs & M. Batley
ABTC		32-002583	RB297		KY485903	RL827	Zon-	murrayensis	Pascoe River X-ing	QLD	143.00979	-12.88309	1/07/2007	R. Leijs & M. Batley
SAM-DNA-Tissue		32-002589	RB298		KY485904	RL829	Zon-	murrayensis	Pascoe River X-ing	QLD	143.00979	-12.88309	1/07/2007	R. Leijs & M. Batley
SAM-DNA-Tissue		32-002590	RB299		KY485876	RL830	Zon-	murrayensis	Pascoe River X-ing	QLD	143.00979	-12.88309	1/07/2007	R. Leijs & M. Batley
ABTC		32-002573	RB300		KY485821	RL834	Zon-	asserta	Iron Range	QLD	143.23521	-12.74302	1/07/2007	R. Leijs & M. Batley
SAM-DNA-Tissue		32-002591	RB306		KY485905	RL853	Zon-	murrayensis	Wenlock River X-ing	QLD	142.94161	-13.09570	2/07/2007	R. Leijs & M. Batley
SAM-DNA-Tissue		32-002624	RB308		KY485759	RL857	Zon-	viridicingulata	Cooktown	QLD	145.24130	-15.48977	3/07/2007	R. Leijs & M. Batley
SAM-DNA-Tissue		32-002625	RB309		KY485760	RL859	Zon-	viridicingulata	Cooktown	QLD	145.24130	-15.48977	3/07/2007	R. Leijs & M. Batley
SAM-DNA-Tissue		32-002623	RB312		KY485909	RL867	Zon-	indistincta	Millstream Falls	QLD	145.45882	-17.64266	4/07/2007	R. Leijs & M. Batley
AM	K.	290969	RB314		KY485832		Zon-	adelaidae	Mataranka 40 km N		132.80278	-14.71611	9/05/2008	M. Batley
AM	K.	290966	RB317		KY485834		Zon-	adelaidae	Katherine 180 km SW	NT	131.28389	-15.54083	10/05/2008	M. Batley
AM	K.	290962	RB319		KY485830		Zon-	adelaidae	Katherine 180 km SW	NT	131.16528	-15.60778	12/05/2008	M. Batley
AM			RB320		KY485839		Zon-	adelaidae	Barkly Homestead 40 km W		135.51583	-19.49083	14/05/2008	M. Batley
AM	K.	290961	RB322		KY485835		Zon-	adelaidae	Barkly Homestead 2 km NW		135.81278	-19.70139	14/05/2008	M. Batley
AM	K.	290959	RB324		KY485838		Zon-	adelaidae	Quamby 56 km N		140.20944	-19.88306	15/05/2008	M. Batley
AM	K.	290955	RB326		KY485837		Zon-	adelaidae	Charters Towers 40 km N		146.02306	-19.81611	17/05/2008	M. Batley
AM			RB333		KY485840		Zon-	adelaidae		NT				M. Batley
ABTC		78814	RB014			RL349	Zon-	murrayensis	Sunny Side, N of Murray Bridge	SA	139.36000	-35.05000	28/12/2003	R.Leijs & K.Hogendoorn
SAM-DNA-Tissue			RB020			RL106	Not-	chlorocyanea	Finke Springs	SA	138.73144	-30.53056	22/01/2005	R.Leijs
AM			RB032				Not-	chlorocyanea	5 km N Wentworth	NSW	141.56000	-34.04000	5/01/2004	M. Batley
AM			RB033				Not-	chlorocyanea	Mt Canobolas	NSW	148.98000	-33.33000	1/01/2004	M. Batley
AM			RB034			RL377	Zon-	asserta	Pennant Hills	NSW	151.06667	-33.73333	17/12/2003	M. Batley
ABTC			RB066		KY485860	RL479	Zon-	murrayensis	Orroroo ex nest	SA	138.52000	-32.98000	1/08/2005	R.Leijs & K.Hogendoorn
ABTC		32-002610	RB079			RL488	Zon-	pulchra	Northbridge	NSW	151.21667	-33.80000	27/02/2003	M.Bell
ABTC			RB102			RL548	Zon-	cingulata	Victoria Point	QLD	153.31667	-27.58333	13/04/2003	J. Klumpp
ABTC			RB104			RL550	Zon-	pulchra	Victoria Point	QLD	153.31667	-27.58333	13/04/2003	J. Klumpp
ABTC			RB108			RL573	Not-	chlorocyanea		SA	138.59848	-32.63877	3/01/2006	R.Leijs & K.Hogendoorn
ABTC			RB110			RL576	Zon-	murrayensis		SA	138.69324	-34.06590	3/01/2006	R.Leijs & K.Hogendoorn
ABTC			RB112			RL595	Zon-	murrayensis		SA	138.53936	-32.98443	3/01/2006	R.Leijs & K.Hogendoorn
ABTC		32-002582	RB113			RL598	Zon-	murrayensis		SA	139.00634	-33.63083	3/01/2006	R.Leijs & K.Hogendoorn
ABTC			RB115			RL606	Zon-	murrayensis		SA	138.51794	-32.92272	3/01/2006	R.Leijs & K.Hogendoorn
ABTC			RB118			RL625	Zon-	murrayensis		SA	138.55427	-32.83349	3/01/2006	R.Leijs & K.Hogendoorn
ABTC			RB119			RL631	Zon-	murrayensis		SA	138.61706	-32.73105	3/01/2006	R.Leijs & K.Hogendoorn
ABTC		32-002596	RB126			RL713	Zon-	walkeri	Darwin, East Point	NT	130.83000	-12.41300	28/02/2006	D.A.Young
ABTC		32-002593	RB127			RL714	Zon-	walkeri	Darwin, East Point	NT	130.83000	-12.41300	28/02/2006	D.A.Young
SAM-DNA-Tissue			RB138			RL683	Not-	chlorocyanea	Botanical Garden Pt. Augusta	SA	137.74431	-32.46532	8/04/2006	R.Leijs
ABTC			RB139			RL666	Not-	chlorocyanea		SA	138.22822	-33.24223	3/01/2006	R.Leijs & K.Hogendoorn
ABTC			RB141			RL568	Not-	chlorocyanea		SA	138.51989	-35.44167	3/01/2006	R.Leijs & K.Hogendoorn
ABTC			RB142			RL653	Not-	chlorocyanea		SA	138.51989	-35.44167	3/01/2006	R.Leijs & K.Hogendoorn
AM	K.	290891	RB164		KY485797		Zon-	asserta	Beecroft	NSW	151.06667	-33.73333	3/01/2006	M. Batley
AM	K.	290892	RB165				Zon-	asserta	Beecroft	NSW	151.06667	-33.73333	8/02/2006	M. Batley
AM	K.	290879	RB168				Zon-	asserta	55 km N of Windsor	NSW	150.66667	-33.23333	22/02/2006	M. Batley
SAM-DNA-Tissue			RB176			MB25	Zon-	murrayensis	Pilbara	WA	117.90000	-22.35000	30/09/2004	M. Batley
AM	K.	290941	RB178				Not-	chlorocyanea	Pilbara	WA	117.70000	-22.30000	13/04/2005	M. Batley
SAM-DNA-Tissue			RB178			MB27	Not-	chlorocyanea	Pilbara	WA	117.70000	-22.30000	13/04/2005	M. Batley
AM	K.	290942	RB179				Not-	chlorocyanea	Pilbara	WA	117.68333	-22.48333	30/09/2004	M. Batley
SAM-DNA-Tissue			RB179			MB28	Not-	chlorocyanea	Pilbara	WA	117.68333	-22.48333	30/09/2004	M. Batley
SAM-DNA-Tissue			RB180			MB29	Not-	chlorocyanea	Pilbara	WA	117.70000	-22.30000	18/11/2004	M. Batley
SAM-DNA-Tissue			RB181			MB30	Not-	chlorocyanea	Pilbara	WA	117.70000	-22.30000	30/09/2005	M. Batley
AM	K.	290944	RB182				Not-	chlorocyanea	Pilbara	WA	117.80000	-22.45000	30/09/2004	M. Batley
SAM-DNA-Tissue			RB182			MB31	Not-	chlorocyanea	Pilbara	WA	117.80000	-22.45000	30/09/2004	M. Batley
SAM-DNA-Tissue			RB183			MB32	Not-	chlorocyanea	Pilbara	WA	118.53333	-22.88333	1/10/2004	M. Batley
ABTC			RB184			RL514	Zon-	asserta	Victoria Point	QLD	153.31667	-27.58333	16/04/2003	J. Klumpp
AM	K.	290909	RB187				Not-	chlorocyanea	Moree 18 km SE	NSW	149.96667	-29.56667	10/01/2007	M. Batley
AM	K.	290895	RB197				Zon-	pulchra	Bribie Is	QLD	153.14889	-27.04944	15 Jan 2007	M. Batley
M+SAM-DNA-Tissue	K.	290920	RB200			MB49	Zon-	pulchra	Victoria Point	QLD	153.30000	-27.58333	16/01/2007	M. Batley
AM	K.	290924	RB201				Zon-	cingulata	Victoria Point	QLD	153.31667	-27.58333	16/01/2007	M. Batley
SAM-DNA-Tissue			RB201			MB50	Zon-	cingulata	Victoria Point	QLD	153.31667	-27.58333	16/01/2007	M. Batley
SAM-DNA-Tissue			RB203			MB52	Zon-	cingulata	Victoria Point	QLD	153.30000	-27.56667	16/01/2007	M. Batley
ABTC			RB218		KY485849	RL493	Zon-	pulchra	Oakdale	NSW	150.50000	-34.06667	23/02/2002	M.Eden
ABTC			RB220			RL541	Zon-	adelaidae	8K SE Mareeba	QLD	145.41667	-16.98333	14/04/2003	R. Storey
ABTC			RB236			RL758	Not-	chlorocyanea	Adelaide Botanical Gardens	SA	138.60896	-34.60896	11/01/2007	R.Leijs & K.Hogendoorn
ABTC			RB252		KY485751	RL735	Not-	chlorocyanea	Torrumbarry Weir	VIC	144.46455	-35.94369	6/01/2007	R.Leijs & K.Hogendoorn
ABTC			RB254			RL747	Not-	chlorocyanea	Koondrook	VIC	144.12634	-35.65201	6/01/2007	R.Leijs & K.Hogendoorn
ABTC		32-002616	RB266		KY485827	RL502	Zon-	adelaidae	Berimah Researh Farm Orchard	NT	130.91667	-12.43333	14/05/2003	G.R. Brown & H Wallace
ABTC			RB288			RL797	Zon-	thorogoodi	N of Bamaga	QLD	142.42316	-10.84117	28/06/2007	R. Leijs & M. Batley
ABTC			RB289			RL800	Zon-	thorogoodi	N of Bamaga	QLD	142.42316	-10.84117	28/06/2007	R. Leijs & M. Batley
AM	K.	290971	RB313		KY485831		Zon-	adelaidae	Hi-way Inn 86 km N		133.27194	-15.64917	9/05/2008	M. Batley
AM	K.	290970	RB315		KY485833		Zon-	adelaidae	Mataranka 40 km N		132.80278	-14.71611	9/05/2008	M. Batley
AM	K.	290965	RB316				Zon-	adelaidae	Katherine 180 km SW	NT	131.28389	-15.54083	10/05/2008	M. Batley
SAM-DNA-Tissue			RB316			508-16A	Zon-	adelaidae	Katherine 180 km SW	NT	131.28389	-15.54083	10/05/2008	M. Batley
SAM-DNA-Tissue			RB317			508-16B	Zon-	adelaidae	Katherine 180 km SW	NT	131.28389	-15.54083	10/05/2008	M. Batley
AM	K.	290964	RB318				Zon-	karlba	Litchfield NP	NT	130.69917	-13.20278	11/05/2008	M. Batley
SAM-DNA-Tissue			RB319			508-24A	Zon-	adelaidae	Katherine 180 km SW	NT	131.16528	-15.60778	12/05/2008	M. Batley
AM	K.	290960	RB321				Zon-	adelaidae	Barkly Homestead 2 km NW		135.81278	-19.70139	14/05/2008	M. Batley
AM	K.	290958	RB323		KY485836		Zon-	adelaidae	Quamby 56 km N		140.20944	-19.88306	15/05/2008	M. Batley
AM	K.	290953	RB327				Zon-	asserta	Springsure 17 km SE		148.20111	-24.21639	18/05/2008	M. Batley
SAM-DNA-Tissue			RB327			508-43A	Zon-	asserta	Springsure 17 km SE		148.20111	-24.21639	18/05/2008	M. Batley
AM	K.	290927	RB328				Zon-	adelaidae	N Mataranka		132.08361	-14.75111	5/04/2008	G. Williams & W. Pulawski
AM			RB329				Zon-	murrayensis	Howard Springs Res.		131.05000	-12.45000	25/04/2008	G. Williams & W. Pulawski
AM	K.	290926	RB329				Zon-	walkeri	Howard Springs Reserve, Darwin	NT	131.05000	-12.45000	25/04/2008	K. Williams & W. Pulawski
SAM-DNA-Tissue			RB333				Zon-	"A"		NT				M. Batley
			RB348	KY485623			Asaro poda	bombylans						
			RB359	KY485696			Zon-							
